# Lewis Acid-Catalyzed Divergent (4+3)/(4+2) Annulations of *o*‑Quinone Methides with Bicyclo[1.1.0]butanes Lead to sp^3^‑Rich Oxabicyclic Frameworks

**DOI:** 10.1021/acs.orglett.5c04897

**Published:** 2026-01-09

**Authors:** Soumik Mondal, Manveer Patel, Subhadeep Hazra, Saumen Hajra, Jaideep Saha

**Affiliations:** † Department of Biological and Synthetic Chemistry, Centre of Biomedical Research, Lucknow 226014, India; ‡ Department of Medicinal Chemistry, National Institute of Pharmaceutical Education and Research (NIPER), Mohali 160062, India; § Academy of Scientific and Innovative Research (AcSIR), Ghaziabad 201002, India; ∥ University of Kalyani, Kalyani 741235, India

## Abstract

Benzannulation strategies that attach a planar arene ring to cyclic, saturated, three-dimensional bridged motifs are becoming valuable for drug discovery. Herein, a Lewis acid-catalyzed [4+3] annulation of *in situ*-generated *o*-QMs with bicyclo[1.1.0]­butanes is developed. Reaction modulation further leveraged a telescoped [4+2] annulation of *o*-QMs with the *in situ*-formed cyclobutanes, leading to diversified sp^3^-rich oxabicyclic architectures. Overall, this study expands the scope for a new bioisosteric space for medicinal chemistry.

## Graphical abstract

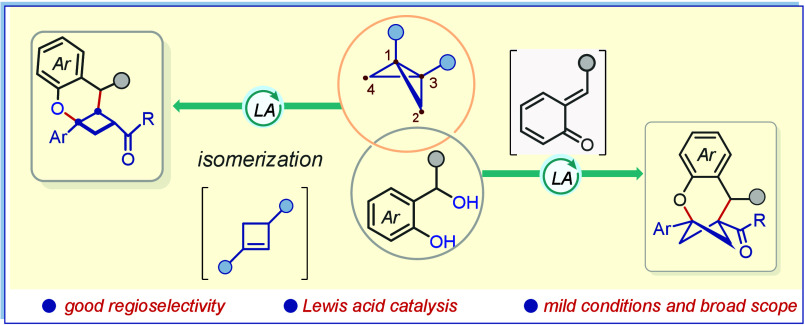

The development of efficient annulation reactions remains pivotal to synthetic chemistry, as cyclic scaffolds are pervasive in natural products and bioactive molecules.^1^ Their ring structures significantly influence molecular characteristics like lipophilicity, three-dimensional conformation, and rigidity, which are key factors in determining their chemical reactivity and biological activity.^2^ Studies suggest that enhancing the sp^3^ character of molecules, often termed “escape from flatland”,^3^ has driven a surge in the application of saturated bicyclic scaffolds in medicinal chemistry over the past decade, largely due to their potential as bioisosteres for phenyl or pyridinyl groups.^4^ Replacing aromatic rings with these conformationally rigid structures increases the proportion of sp^3^-hybridized carbon atoms in a molecule, a strategy that is increasingly being recognized as a powerful tool for optimizing the physicochemical and pharmacokinetic properties of drug candidates.^5^ Importantly, bicyclic scaffolds generated from BCBs through addition, rearrangement, and insertion reactions are inherently rich in sp^3^-hybridized carbons, making them attractive building blocks for drug design.^6^ Recent advances have enabled the synthesis of heteroatom-substituted bicyclo[2.1.1]­hexanes^7^ and bicyclo[3.1.1]­heptanes,^8^ predominantly through strain-release functionalization of bicyclo[1.1.0]­butanes (BCBs). These architectures embody saturated, conformationally restricted, sp^3^-rich frameworks, thereby serving as privileged bioisosteres that broaden the accessible three-dimensional chemical space relevant to medicinal chemistry. Notably, heterobicyclo[4.1.1]­octanes (BCOs) and fused 2-oxabicyclo[4.2.0]­octane, though valuable, have been far less explored than their all-carbon analogues.^9^ Some advances in this context have been made with the use of quinones and preformed *o*-QM,^10^ which underwent metal Lewis acid-catalyzed [3+2] and [4+2] annulations with BCB, leveraging the formation of bicyclo[4.1.1]­octane scaffolds.^9g,9h^ Importantly, the anticipated [4+3] annulation product was not achieved from the reaction between the BCB and the preformed *o*-QM; instead, an isomerized structure was achieved under the employed metal-catalyzed condition. In line with our continuing interest in engaging strained rings with *in situ*-generated *o*-QMs,^11^ we questioned whether employing the BCB with the former under a different reaction condition could allow access to the elusive oxabicyclo[4.1.1]­octane scaffolds. It was also anticipated that a successful development would expand the synthetic repertoire around this interesting scaffold. We herein disclose a modular strategy to access both a distinct type of oxabicyclo[4.1.1]­octane scaffolds and 2-oxabicyclo[4.2.0]­octanes under Yb-catalyzed reaction conditions (Scheme 1C).

**Scheme 1:**
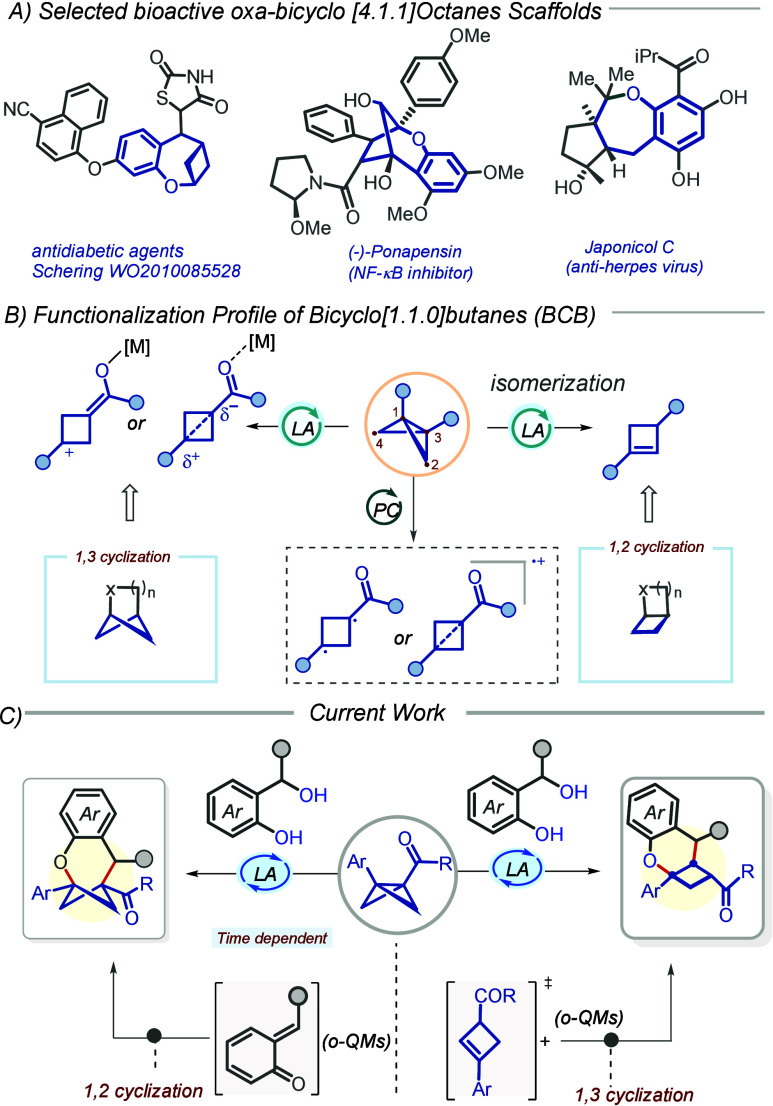
(A) Various Reactivities of BCBs, (B) Prior Work, and (C) the Current Work

At the outset, we screened various precursor compounds^12^ that could transform into an *o*-QM *in situ* and simultaneously engage in the desired [4+3] cycloaddition with BCB. Notably, an orthogonal activation to generate the *o*-QM from its precursor, without altering BCB or the cycloaddition step, was needed. We also envisaged a dual activation of the two interlinked steps. For this purpose, 2-(hydroxy­(phenyl)­methyl)­phenol (**1a**) was identified as the most suitable precursor to *o*-QM, which delivered compound **3** with BCB **2a** in the presence of 5% Yb­(OTf)_3_ (Table 1, entry 1). Use of a 7:3 THF/HFIP solvent mixture worked optimally for this reaction (entry 1). Importantly, other Lewis acids such as Bi­(OTf)_3_, Ga­(OTf)_3_, Sc­(OTf)_3_, and AgOTf performed less effectively under the identical reaction conditions (entries 2–5, respectively).

**Table 1:**
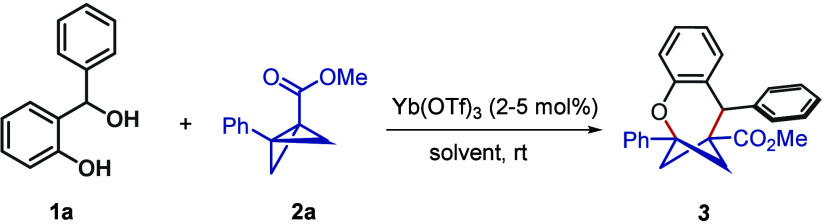
Optimization Studies^a^

entry	catalyst	solvent	yield (%)^b^
1	Yb(OTf)_3_	7:3 THF/HFIP	74
2	Bi(OTf)_3_	7:3 THF/HFIP	25
3	Ga(OTf)_3_	7:3 THF/HFIP	48
4	AgOTf	7:3 THF/HFIP	40
5	Sc(OTf)_3_	7:3 THF/HFIP	35
6^c^	Yb(OTf)_3_	7:3 THF/HFIP	45
7^d^	Yb(OTf)_3_	7:3 THF/HFIP	30
8	–	7:3 THF/HFIP	–
9	Yb(OTf)_3_	7:3 DCM/HFIP	15
10	Yb(OTf)_3_	7:3 toluene/HFIP	35
11	Yb(OTf)_3_	only THF	30
12	Yb(OTf)_3_	only HFIP	35
13	Yb(OTf)_3_	1:1 THF/HFIP	40
14^e^	Yb(OTf)_3_	7:3 THF/HFIP	33
15^f^	Yb(OTf)_3_	7:3 THF/HFIP	20
16^g^	Yb(OTf)_3_	7:3 THF/HFIP	–

a Reaction conditions: **1a** (1.5 equiv), **2a** (1.0 equiv), catalyst (5.0 mol %), rt, 0.1 M solvent.

b Isolated yield.

c With 10 mol % catalyst.

d With 2.0 mol % catalyst.

e With 0.2 M solvent.

f With 0.5 M solvent.

g With 1.0 M solvent.

Changing the catalyst loading from 5 mol % to 2 or 10 mol % appeared to result in a detrimental outcome (entry 6 or 7, respectively). No reaction proceeded in the absence of a catalyst (entry 8). We also screened other solvents in combination with HFIP such as DCM and toluene (entries 9 and 10, respectively) or acetonitrile (not shown), which rendered less conversion compared to that of THF. Importantly, neither THF nor HFIP alone was a suitable choice for the solvent compared to their combination (entries 11 and 12). A 1:1 mixture was also less effective (entry 13), underscoring the importance of an optimized ratio between these two polar aprotic and protic solvents. An increase in the solvent concentration was largely detrimental to the transformation (entries 14–16).

Having established the optimal reaction conditions, we first proceeded to investigate a diverse set of aromatic ring-substituted 2-(hydroxy­(phenyl)­methyl) phenols as precursors to generate *o*-quinone methides *in situ* (top panel of Scheme 2). Aromatic residues containing electron-donating, neutral, and electron-withdrawing substituents at the benzylic position of phenol underwent smooth conversion. For instance, *para* substitution with alkyl groups (isopropyl/*tert*-butyl), phenyl, -OPh, -OCF_3_, and -F afforded the desired bridged bicycles in moderate to good yields and a high degree of regioselectivity (**4–9**). Those with various *meta* substitutions were also readily accommodated (**10–12**). The X-ray crystal structure of **12** unambiguously confirms the structure of the bicyclic product, and the same could be explained for the other entries based on reactive similarity. Entries **13–15** represent examples of a heterocycle (2-thiophene), a π-extended (1-naphthyl) moiety, and an alkyl-containing *o*-QM, respectively, which were also well tolerated under the reaction conditions. We also performed some variation on the phenolic aromatic ring, and those were also competent (**16** and **17**).

**Scheme 2:**
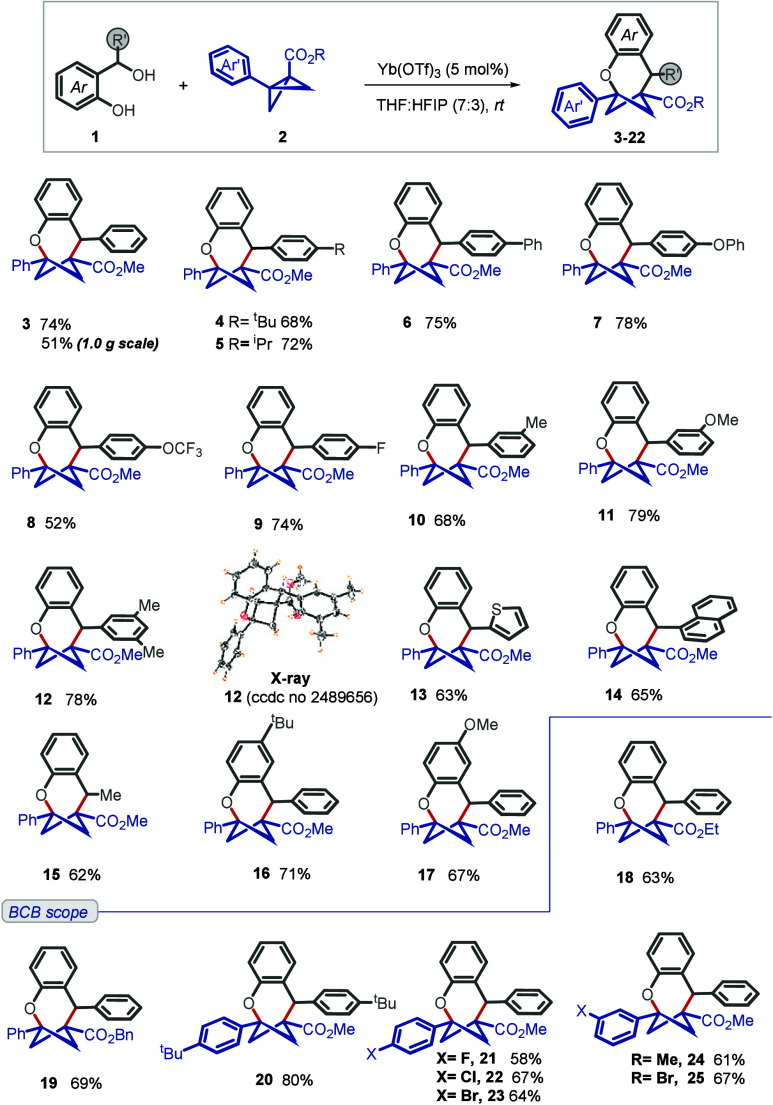
Scope for the Synthesis of Oxabicyclo[4.1.1]­octanes^a^

a Reaction conditions: **1** (0.15 mmol, 1.5 equiv), **2** (0.1 mmol, 1.0 equiv), catalyst (5.0 mol %), rt, 0.1 M solvent.

Variation of BCB was also performed. Variation of the ester residue (**18** and **19**) and substitution on the aromatic ring of BCB could be accommodated without a significant difference in reactivity (**20–25**). A 1.0 g scale reaction was also performed, which delivered compound **3** in 51% isolated yield.

Lewis acid-promoted isomerization of BCB to cyclobutene has been reported in the literature (Scheme 1B).^13^ We questioned whether a telescopic reaction could be developed that involves two *in situ*-generated intermediates under the same Lewis acid activation, i.e., an *in situ*-generated cyclobutene intermediate (**1′** (Scheme 3)) from the BCB and the *o*-QM intermediate from its 2-(hydroxy­(phenyl)­methyl)­phenol precursor. Then, a productive union of these two species via [4+2] cycloaddition could render 2-oxabicyclo[4.2.0]­octanes. Gratifyingly, this reaction course could be accessed under similar conditions, albeit under a telescopic setup and first developed using tolyl-substituted BCB (**2j**) and *o*-QM precursor **2a** as model substrates. Importantly, the reaction proceeded with a high degree of regioselectivity for cycloaddition, placing the two aryl residues adjacent to the cyclobutene ring in the relative *anti* disposition. The benzylic proton of **26** at 4.26 ppm showed a doublet (6.6 Hz), which confirmed the regioselectivity. The scope of the reaction was evaluated employing compound **2** having a diverse range of aryl residues at the benzylic position, including an extended π-system and heteroaryl; all successfully delivered the desired fused cyclobutene scaffold in moderate to good yields (**26–40**). It should be noted that performing the reaction with the isolated cyclobutene from BCB (**2j″**) instead of telescoped formation resulted in an only marginal increase (62%) in the yield.

**Scheme 3:**
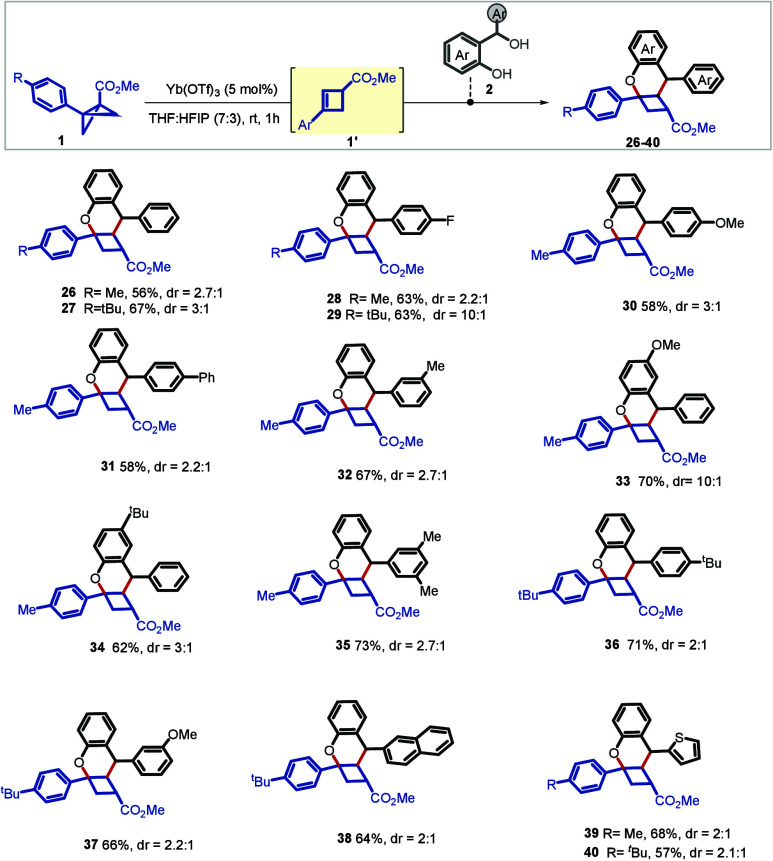
Scope for Access to 2-Oxabicyclo[4.2.0]­octanes^a^

a Reaction conditions: **1** (0.15 mmol, 1.5 equiv), **2** (0.1 mmol, 1.0 equiv), catalyst (5.0 mol %), rt, 4 h, 0.1 M solvent. After 1 h, the formation of intermediate **1′** was confirmed by TLC; compound **2** was then added to afford the final product.

We next performed some follow-up transformations on the selected bridged and fused bicyclic products from Schemes 2 and 3. For instance, reduction of the ester group in **3** with LiAlH_4_ furnished primary alcohol compound **41** (Scheme 4a, top panel). Oxidation of the former with PCC then afforded aldehyde **42**. Similarly, reduction of the ester of compound **26** delivered primary alcohol **43** in good yield (bottom panel). We also explored the precursor to aza-*o*-QM (**44** and **46**) under identical reaction conditions to determine the feasibility of obtaining analogous aza-bicyclic compounds (Scheme 4b). However, the process appears to be not compatible or efficient to deliver the desired products (**45/47**). Based on the literature precedents and our observations, a plausible mechanism leading to two types of bicyclic products obtained in the study is presented in Scheme 4c.^9,13^ We speculate that HFIP can act synergistically with the Lewis acid by stabilizing polarized transition states and reactive intermediates (e.g., *o*-QMs and BCB-derived species) through strong hydrogen bonding, leading to enhanced reaction rates. Once the BCB converts into the enolate (**II**), it undergoes nucleophilic addition onto *o*-QM forming **III**, which then undergoes a ring closure to **3** through phenolic oxygen being added to the benzylic position. A fused cyclobutene derivative is formed in a similar manner, except in this case, the cyclobutene as the nucleophile would have added to *o*-QM (**IV**) and the final ring closure leads to **26**.

**Scheme 4:**
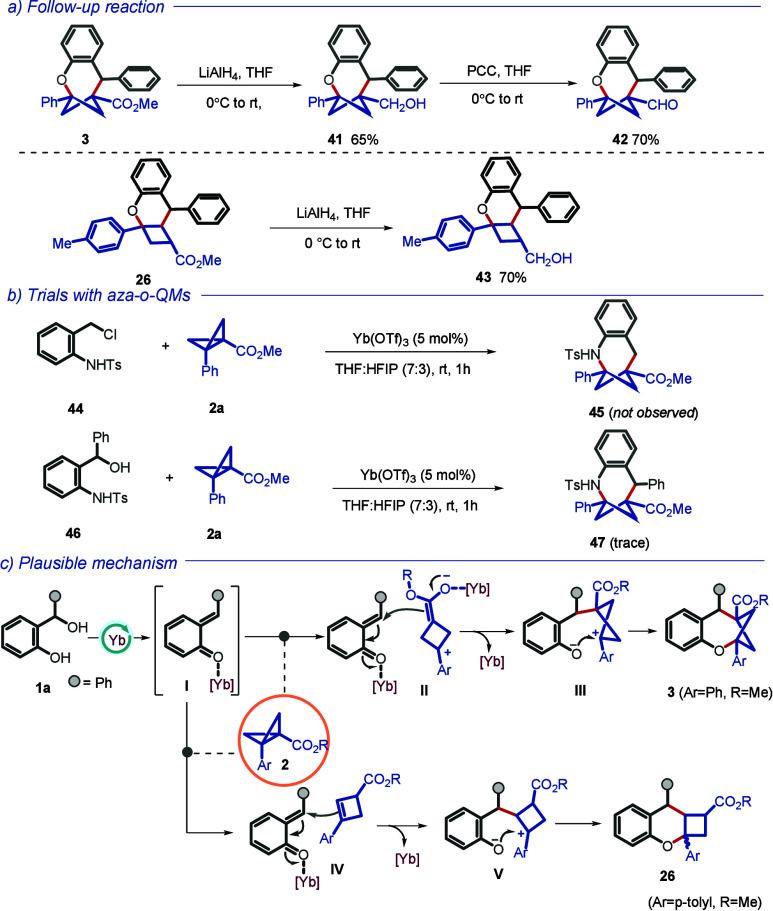
(a) Follow-up Reactions, (b) Trials with Aza-*o*-QM, and (c) a Plausible Mechanism

In summary, the current study showcases a Yb-catalyzed approach for the construction of two different types of oxabicyclo[4.1.1]­octanes involving *in situ*-generated *o*-QMs and BCBs. In one type of reaction, a direct [4+3] cycloaddition led to oxabicyclo[4.1.1]­octanes under the optimized conditions. In the other case, a telescoped transformation has been developed that capitalizes on the *in situ* formation of cyclobutene from BCB. This process involves a formal [4+2] cycloaddition between the former moiety and *o*-QMs under similar reaction conditions, leading to 2-oxabicyclo[4.2.0]­octanes with a high degree of regioselectivity. The choice of precursors for *o*-QM turned out to be a critical factor in the successful development of the process.

## Supplementary Material



## Data Availability

The data underlying this study are available in the published article and its Supporting Information.

## Notes

General experimental methods, experimental data, and copies of ^1^H and ^13^C NMR spectra of the products (PDF)

## ∇

J.S.: Molecular Design and Synthesis Unit, Rutgers, The State University of New Jersey, Piscataway, NJ 08854
