# Supplementary material for: Lewis Acid-Catalyzed Divergent (4+3)/(4+2) Annulations of o‑Quinone Methides with Bicyclo[1.1.0]butanes Lead to sp3‑Rich Oxabicyclic Frameworks
Source: Org Lett. 2026 Jan 9;28(3):954–9. doi: 10.1021/acs.orglett.5c04897 (PMC12836357; doi:10.1021/acs.orglett.5c04897)
Supplement: Supplementary file 1 [file ol5c04897_si_001.pdf]

Supporting Information

Lewis Acid–Catalyzed Divergent (4+3)/(4+2) Annulations of ortho-Quinone Methides with Bicyclo[1.1.0]butanes Lead to sp³-Rich Oxa-Bicyclic Frameworks

Soumik Mondal,[a,c]ǂ Manveer Patel,[a]ǂ Subhadeep Hazra,[b,d] Saumen Hajra* [a,c] and Jaideep Saha*[b,†]

[a]Department of Biological and Synthetic Chemistry, Centre of Biomedical Research, Lucknow-226014, India. [b]Department of Medicinal Chemistry, National Institute of Pharmaceutical Education and Research (NIPER), Mohali-160062, India. [†]Molecular Design and Synthesis Unit, Rutgers, The State University of New Jersey, Piscataway, New Jersey 08854, United States. [c]Academy of Scientific and Innovative Research (AcSIR), Ghaziabad 201002, India. [d]University of Kalyani, 741235, India

*Email: jdsaha2000@gmail.com; js3954@rutgers.edu

Table of content

Entry	Description	Page
**1**	General Information……………………………………………….	3
**2**	Preparation of Starting Materials…………………………………...	4-7
**4**	Reaction optimization……………………………………………..	8
**5**	General Procedure for the HFIP-mediated (4+3) Annulation of BCBs with diols…………………………………………………..	9
**6**	Procedure for the 1.0 gram Scale Reaction for the synthesis of **3**…...	10
**7**	Preparation and Characterization of compound (**3-25**)……………	11-23
**8**	General Procedure for the HFIP-mediated (4+2) Annulation of BCBs with diols……………………………………………………	24
**9**	Preparation and Characterization of compound (**26-40**)…………………………………………………………………..	25-34
**10**	Product Modifiaction………………………………	35-36
**11**	NMR spectra of new compounds (**3 to 43**) …………………..	37-77
**12**	1D NMR spectra for compound **29** ………………………………	78
**13**	X-Ray Crystallography ………………………………………………	79-80
**14**	References ……………………………………………………………	81

1. General Information

All reagents were purchased from commercial suppliers and used without further purification. Reactions were monitored by thin-layer chromatography using 0.2 mm commercial silica gel plates (silica gel 60, F254, EMD Chemical). Column chromatography was performed using silica gel (100−200 mesh). The vials (Wheaton® Standard Scintillation Vials, 1 dram, 15x45 mm with PTFE lined cap attached) were purchased from Borosil and dried in an oven overnight before use. Anilines, pentafluorophenol, and aldehydes were purchased from Sigma-Aldrich, TCI, or Alfa Aesar. Highresolution mass spectra (HRMS) were recorded on a mass spectrometer using electrospray ionizationtime of-flight (ESITOF) reflectron experiments. All reactions were run in flame- or oven-dried glassware with dry solvents unless otherwise stated. All ^{1}H-NMR were recorded on 400 MHz and 500 MHz spectrometers (Bruker) and ^{13}C-NMR were recorded on 101 MHz and 126 MHz using $CDCl_3$ solvent, the chemical shifts are reported as parts per million (ppm) referenced to solvent residual peak; $CDCl_3$ (^{1}H= 7.26 ppm, ^{13}C= 77.16 ppm). Coupling constants are reported in Hertz (Hz). Data for ^{1}H NMR spectra are reported as follows: chemical shift (ppm, referenced to proton; s = singlet, d = doublet, t = triplet, q = quartet, dd = doublet of doublets, td = triplet of doublets, m = multiplet). All the coupling constants are measured in Hz. All the products were characterized through ^{1}H NMR, ^{13}C NMR.

2. Starting Materials

2.1 List of diols used in this study.

The diols (1a – 1s) were prepared according to previously reported procedures.[1] 1n was commercially available and used without purification.

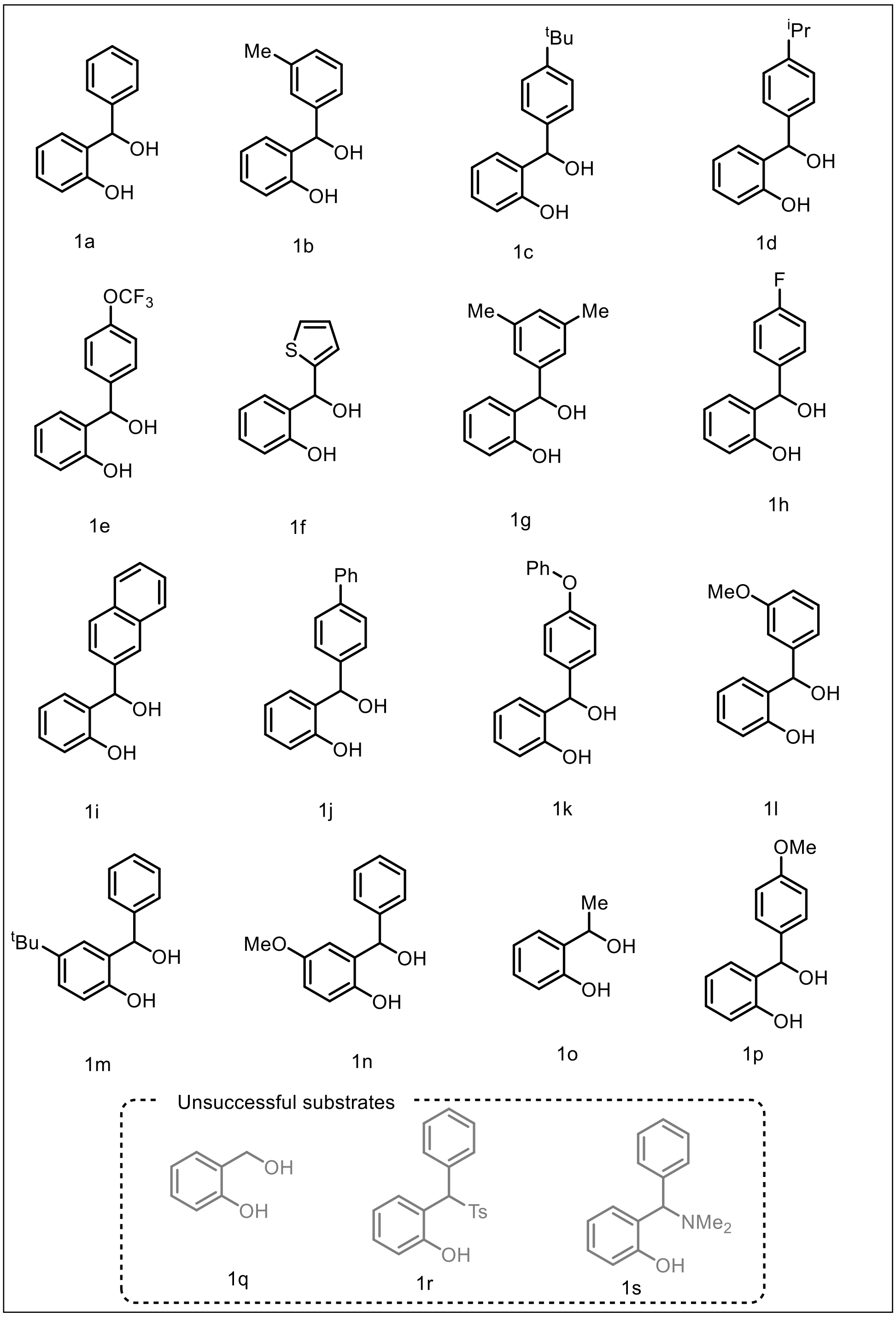

Figure S1. Scope of diols used in the study.

2.2 Preparation of BCBs

All bicyclo[1.1.0]butanes are prepared according to the known literature procedures and were synthesized according to the Scheme given below. The spectral data of the synthesized BCBs matched with the reported values in the literature.[2]

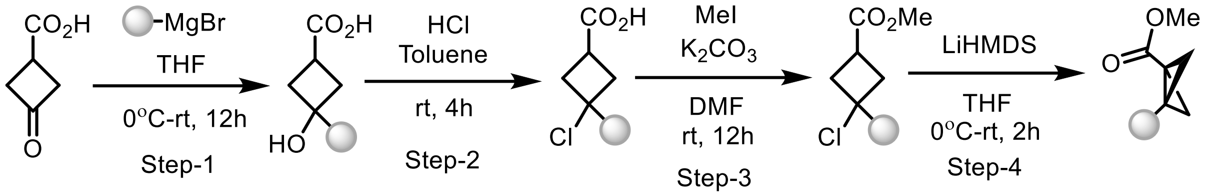

Step 1: A flame-dried 2-neck round-bottom flask charged with 3-oxocyclobutane carboxylic acid (10.0 g, 87.6 mmol, 1.0 equiv) was purged with nitrogen and dissolved in anhydrous THF (0.2 M). The reaction mixture was cooled to 0 °C, followed by a slow dropwise addition of Grignard reagent (219.1 mmol, 2.5 equiv) via a syringe through the septum. The reaction was stirred for 12 hours at ambient temperature. Upon completion, the reaction was quenched with water at 0 °C and acidified using concentrated HCl. The aqueous phase was extracted with diethyl ether (3×), and the combined organic extracts were dried over anhydrous $MgSO_4$, filtered, and concentrated under reduced pressure to yield the crude 3-hydroxy-3-arylcyclobutane carboxylic acid, which was used directly in the next step without further purification.

Step 2: The crude 3-hydroxy-3-arylcyclobutane carboxylic acid (81.1 mmol, 1.0 equiv) was dissolved in toluene (0.5 M), followed by the addition of concentrated HCl (97.3 mL, 1.2 equiv). The reaction mixture was stirred at room temperature for 4 hours. Upon completion, the aqueous phase was separated and extracted with toluene. The combined organic layers were washed successively with water and brine, dried over $MgSO_4$, filtered, and concentrated under vacuum to furnish the crude 3-chloro-3-arylcyclobutane carboxylic acid, which was used in the subsequent step without purification.

Step 3: In a nitrogen atmosphere, a solution of crude 3-chloro-3-arylcyclobutane carboxylic acid (103.8 mmol, 1.0 equiv.) in DMF (0.9 M) was treated with K_2CO_3 (28.7 g, 207.6 mmol, 2.0 equiv.) and methyl iodide (9.69 mL, 155.7 mmol, 1.5 equiv.) added dropwise. The mixture was stirred at room temperature for 12 hours. The reaction was

quenched with water and extracted with ethyl acetate. The organic phase was dried over $MgSO_4$, filtered, and concentrated in vacuo to afford crude methyl 3-chloro-3-arylcyclobutane-1-carboxylate, which was carried forward without further purification.

Step 4: Methyl 3-chloro-3-phenylcyclobutane-1-carboxylate (37.5 mmol, 1.0 equiv) was dissolved in anhydrous THF (0.35 M) in a nitrogen atmosphere and cooled to 0 °C. Lithium hexamethyldisilazide (LiHMDS, 1.0 M in THF, 45.0 mL, 45.0 mmol, 1.2 equiv) was added dropwise. After complete addition, the reaction mixture was gradually warmed to room temperature and stirred for an additional 2 hours. The reaction was quenched with saturated ammonium chloride solution, and the product was extracted with ethyl acetate. The combined organic layers were washed with brine, dried over Na_2SO_4, filtered, and concentrated. Purification by flash column chromatography (petroleum ether/EtOAc = 97:3) furnished the desired 3-phenylbicyclo[1.1.0]butane-1-carboxylate derivatives.

List of BCBs prepared in the study

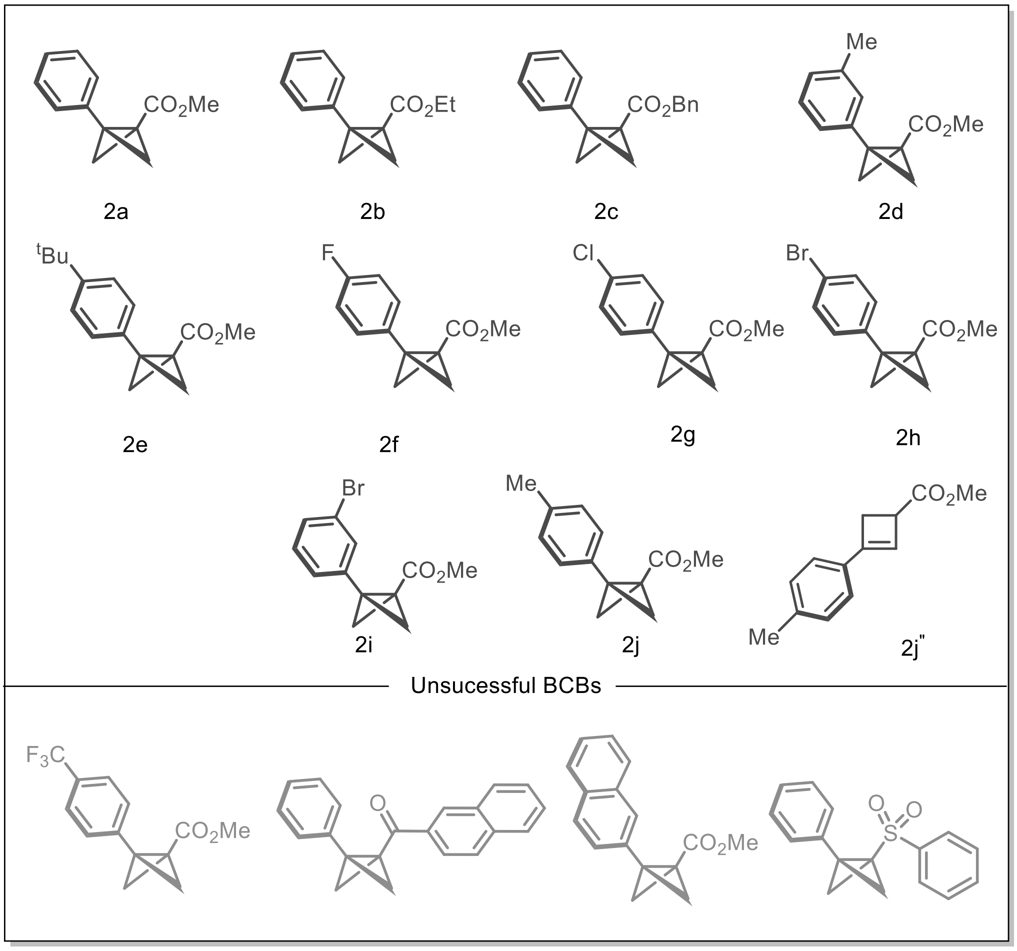

Figure S2. Scope of BCBs used in the study.

3. General Procedure for Optimization of the Reaction Conditions

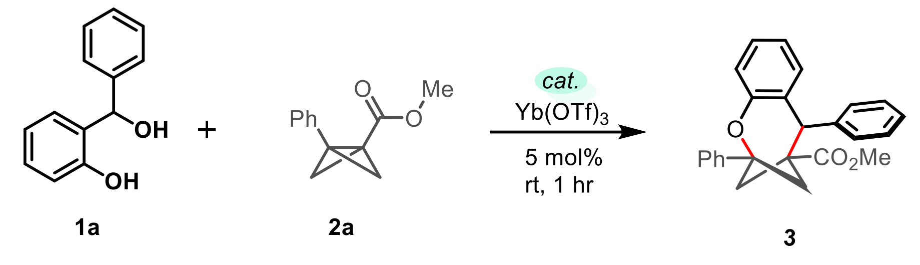

Table S3: Optimization Studies

Entries	Catalyst	Solvent	Yield(%)[b]
1[a]	$Yb(OTf)_3$	THF:HFIP (7:3)	74
2[a]	$Bi(OTf)_3$	THF:HFIP (7:3)	25
3[a]	$Ga(OTf)_3$	THF:HFIP (7:3)	48
4[a]	Ag(OTf)	THF:HFIP (7:3)	40
5[a]	Sc(OTf)3	THF:HFIP (7:3)	35
6[a]	$Yb(OTf)_3$	DCM:HFIP (7:3)	15
7[a]	$Yb(OTf)_3$	Toluene:HFIP(7:3)	35
8[c]	$Yb(OTf)_3$	THF:HFIP (7:3)	45
9[d]	$Yb(OTf)_3$	THF:HFIP (7:3)	30
10[a]	$Yb(OTf)_3$	Only THF	30
11[a]	$Yb(OTf)_3$	Only HFIP	35
12[a]	$Yb(OTf)_3$	THF:HFIP(1:1)	40
13[a]	-	THF:HFIP(7:3)	-
14[e]	$Yb(OTf)_3$	THF:HFIP(7:3)	33
15[f]	$Yb(OTf)_3$	THF:HFIP(7:3)	20

Reaction condition: a= 1a (1.5 equiv.), 2a (1.0 equiv.), cat. (5.0 mol%), rt, 1 h, solvent [0.1M]. b=isolated yield, c= cat. (10.0 mol%), d= cat. (2.0 mol%), e= solvent [0.2 M], f= solvent [0.5 M].

4. General Procedure for the HFIP-mediated (4+3) Annulation of BCBs with diols

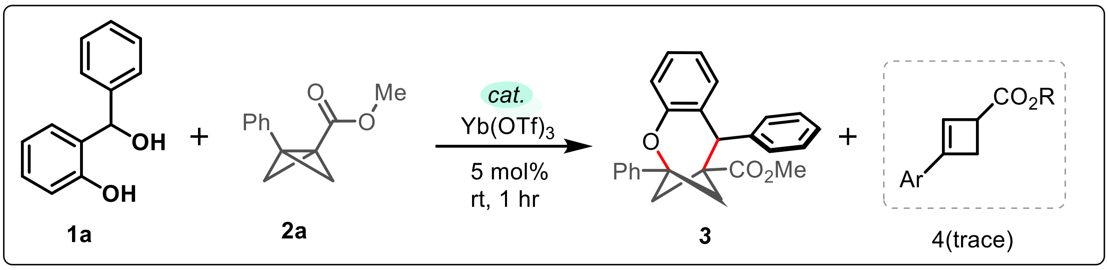

General Procedure (I):

An oven-dried screw-capped test tube equipped with a magnetic stir bar was charged with Diol 1a (0.15 mmol, 1.0 equiv) and bicyclo[1.1.0]butane derivative 2a (0.10 mmol, 1.0 equiv) under a nitrogen atmosphere. Anhydrous THF (THF 0.7 mL) was added to dissolve the reactants, followed by the addition of hexafluoroisopropanol (HFIP, 0.3 mL) at rt, followed by the addition of $Yb(OTf)_3$ (0.005 mmol). The reaction mixture was stirred for 1 hour, Upon completion, the reaction mixture was concentrated under reduced pressure. The resulting crude residue was pre-adsorbed onto silica gel and purified by flash column chromatography (petroleum ether/ethyl acetate as eluent) to afford product 3 in good to excellent yields.

^{1}H NMR of crude reaction mixture

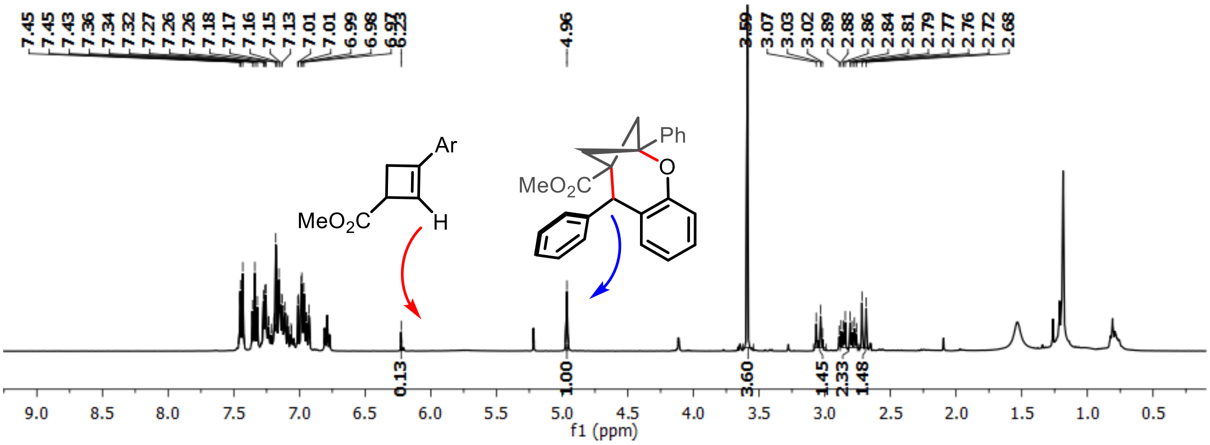

5. Procedure for the 1.0 gram Scale Reaction for the synthesis of 3

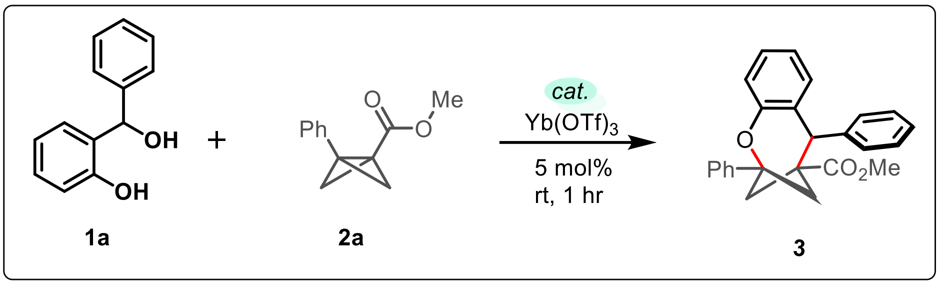

An oven-dried screw-capped test tube equipped with a magnetic stir bar was charged with 2-(hydroxy(phenyl)methyl)phenol 1a (1.3 g, 6.4mmol, 1.2 equiv.) and methyl 3-phenylbicyclo[1.1.0]butane-1-carboxylate 2a (1.0 g, 5.3 mmol, 1.0 equiv.) under a nitrogen atmosphere. Anhydrous THF (THF 36.0 mL) was added to dissolve the reactants, followed by the addition of hexafluoroisopropanol (HFIP 17.0 ml) at rt, followed by the addition of $Yb(OTf)_3$ (0.164g, 0.265 mmol). The reaction mixture was stirred for 1 hour, Upon completion, the reaction mixture was concentrated under reduced pressure. After 1 h, the solvent was evaporated, and the crude residue was pre-adsorbed on silica gel and purified by flash column chromatography (Pet. ether /EtOAc = 95/5) on silica gel to afford 3a as a semi solid (1.0 g, 51% yield).

6. Experimental Details for the Synthesized Compounds:(3-25)

methyl 2,5-diphenyl-2,3-dihydro-2,4-methanobenzo[b]oxepine-4(5H)-carboxylate (3)

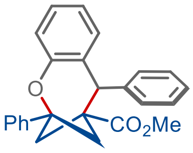

Following the general procedure(I), treatment of diol 1a (0.032 g, 0.15 mmol) and BCB 2a (0.020 g, 0.1 mmol) with $Yb(OTf)_3$ (0.003 g, 0.005 mmol) in THF:HFIP(7:3) (1 mL) at rt for 1hr, delivered compound **3**, which was purified by silica gel column chromatography (EtOAc: Hexane 5:95) to furnish the title compound **3** as semi solid in 74% (0.029g) yield; **^{1}H NMR** (400 MHz, $CDCl_3$) δ 7.54 (d, *J* = 7.4 Hz, 2H), 7.44 (t, *J* = 7.5 Hz, 2H), 7.36 (t, *J* = 7.3 Hz, 1H), 7.29 (d, *J* = 5.4 Hz, 2H), 7.25 – 7.19 (m, 2H), 7.12 – 7.02 (m, 4H), 6.89 (t, *J* = 7.4 Hz, 1H), 5.06 (s, 1H), 3.69 (s, 3H), 3.15 (d, *J* = 13.4 Hz, 1H), 2.97 (dd, *J* = 12.9, 6.1 Hz, 1H), 2.88 (dd, *J* = 13.4, 6.1 Hz, 1H), 2.80 (d, *J* = 12.9 Hz, 1H). **^{13}C {^{1}H} NMR** (100 MHz, CDCl3) δ 175.5, 153.6, 143.8, 142.6, 134.1, 129.1, 128.5,128.4, 128.2, 127.9, 127.1, 125.4, 121.4(2), 80.0, 54.1, 52.2, 45.9, 40.9, 37.6. **HRMS(ESI-TOF)** m/z: $[M+H^+]^+$ $C_{25}H_{23}O_3$ calcd. 371.1647, found. 371.1650.

Methyl-5-(4-(tert-butyl)phenyl)-2-phenyl-2,3-dihydro-2,4-methanobenzo[b]oxepine-4(5H)-carboxylate (4)

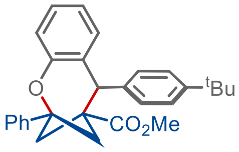

Following the general procedure(I), treatment of diol 1c (0.038 g, 0.15 mmol) and BCB 2a (0.020 g, 0.1 mmol) with $Yb(OTf)_3$ (0.003 g, 0.005 mmol) in THF:HFIP(7:3) (1.0 mL) at rt for 1 hr, delivered compound **4**, which was purified by silica gel column chromatography (EtOAc: Hexane 5:95) to furnish the title compound **4** as semi solid in 68% (0.030g) yield; **^{1}H NMR** (400 MHz, $CDCl_3$) δ 7.55 (d, *J* = 7.4 Hz, 2H), 7.45 (t, *J* = 7.5 Hz, 2H), 7.37 (d, *J* = 7.2 Hz, 1H), 7.29 – 7.25 (m, 2H), 7.21 (t, *J* = 7.6 Hz, 1H), 7.08 (dd, *J* = 12.5, 8.2 Hz, 2H), 6.98 (d, *J* = 8.1 Hz, 2H), 6.90 (t, *J* = 7.3 Hz, 1H), 5.06 (s, 1H), 3.71 (s, 3H), 3.16 (d, *J* = 13.4 Hz, 1H), 2.99-2.86 (m, 2H), 2.79 (d, *J* = 12.9 Hz, 1H), 1.31 (s, 9H). **^{13}C {^{1}H} NMR** (100 MHz, $CDCl_3$) δ 175.5, 153.5, 149.7, 143.9,

139.4, 134.2, 128.6, 128.5, 128.1, 127.9, 125.7, 125.4, 125.3, 121.3(2), 80.1, 53.5, 52.2, 45.8, 40.9, 37.6, 34.5, 31.4. **HRMS(ESI-TOF)** m/z: $[M+H^+]^+$ $C_{29}H_{31}O_3$ calcd. 427.2273, found. 427.2247.

Methyl-5-(4-isopropylphenyl)-2-phenyl-2,3-dihydro-2,4-methanobenzo[b]oxepine-4(5H)-carboxylate (5)

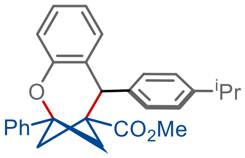

Following the general procedure(I), treatment of diol 1d (0.036 g, 0.15 mmol) and BCB 2a (0.020 g, 0.1 mmol) with $Yb(OTf)_3$ (0.003 g, 0.005 mmol) in THF:HFIP(7:3) (1.0 mL) at rt for 1 hr, delivered compound **5**, which was purified by silica gel column chromatography (EtOAc: Hexane 5:95) to furnish the title compound **5** as semi solid in 72% (0.032g) yield; **^{1}H NMR** (400 MHz, $CDCl_3$) δ 7.54 (d, *J* = 7.4 Hz, 2H), 7.43 (t, *J* = 7.5 Hz, 2H), 7.35 (s, 1H), 7.18 (dd, *J* = 6.7, 5.5 Hz, 1H), 7.14 – 7.05 (m, 4H), 6.98 (d, *J* = 8.1 Hz, 2H), 6.89 (t, *J* = 7.3 Hz, 1H), 5.05 (s, 1H), 3.69 (s, 3H), 3.16 (d, *J* = 13.3 Hz, 1H), 2.96 (dd, *J* = 12.8, 6.1 Hz, 1H), 2.87 (dt, *J* = 13.8, 6.8 Hz, 2H), 2.78 (d, *J* = 12.8 Hz, 1H), 1.24 (d, *J* = 6.9 Hz, 6H). **^{13}C {^{1}H} NMR** (100 MHz, $CDCl_3$) δ 175.5, 153.5, 147.4, 144.0, 139.8, 134.2, 128.9, 128.5, 128.1, 127.8, 126.4, 125.8, 125.4, 121.3 (2), 53.7, 52.1, 45.9, 40.9, 37.7, 33.7, 24.0. **HRMS(ESI-TOF)** m/z: $[M+H^+]^+$ $C_{28}H_{29}O_3$ calcd. 413.2117, found. 413.2101.

Methyl-5-([1,1'-biphenyl]-4-yl)-2-phenyl-2,3-dihydro-2,4-methanobenzo[b]oxepine-4(5H)-carboxylate (6)

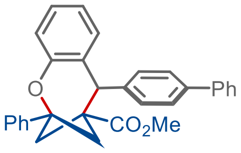

Following the general procedure(I), treatment of diol 1j (0.041 g, 0.15 mmol) and BCB 2a (0.020g, 0.1 mmol) with $Yb(OTf)_3$ (0.003 g, 0.005 mmol) in THF:HFIP(7:3) (1.0 mL) at rt for 1 hr, delivered compound **6**, which was purified by silica gel column chromatography (EtOAc: Hexane 5:95) to furnish the title compound **6** as semi solid

in 75% (0.035g) yield; **^{1}H NMR** (400 MHz, $CDCl_3$) δ 7.57 (t, *J* = 8.0 Hz, 4H), 7.51 (d, *J* = 8.2 Hz, 2H), 7.47-7.42 (m, 4H), 7.38 – 7.33 (m, 2H), 7.23 (t, *J* = 7.0 Hz, 1H), 7.14 (t, *J* = 8.2 Hz, 3H), 7.09 (d, *J* = 7.7 Hz, 1H), 6.92 (t, *J* = 7.4 Hz, 1H), 5.13 (s, 1H), 3.72 (s, 3H), 3.20 (d, *J* = 13.3 Hz, 1H), 3.00 (dd, *J* = 12.9, 6.1 Hz, 1H), 2.92 (dd, *J* = 13.3, 6.1 Hz, 1H), 2.82 (d, *J* = 12.8 Hz, 1H). **^{13}C {^{1}H} NMR** (100 MHz, $CDCl_3$) δ 175.4, 153.6, 143.9, 141.7, 140.8, 139.9, 134.1, 129.5, 128.8, 128.5, 128.3, 127.9, 127.3, 127.1, 125.5, 125.4, 121.5,121.4 80.2, 53.7, 52.2, 45.9, 41.0, 37.7. **HRMS(ESI-TOF)** m/z: $[M+H^+]^+$ $C_{31}H_{27}O_3$ calcd. 447.1960, found. 447.1932.

Methyl-5-(4-phenoxyphenyl)-2-phenyl-2,3-dihydro-2,4-methanobenzo[b]oxepine-4(5H)-carboxylate (7)

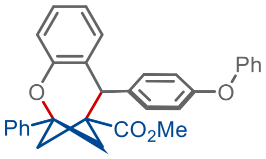

Following the general procedure(I), treatment of diol 1k (0.044 g, 0.15 mmol) and BCB 2a (0.020 g, 0.1 mmol) with $Yb(OTf)_3$ (0.003 g, 0.005 mmol) in THF:HFIP(7:3) (1.0 mL) at rt for 1 hr, delivered compound **7**, which was purified by silica gel column chromatography (EtOAc: Hexane 5:95) to furnish the title compound **7** as semi solid in 78% (0.038g) yield; **^{1}H NMR** (400 MHz, $CDCl_3$) δ 7.54 (d, *J* = 7.4 Hz, 2H), 7.44 (t, *J* = 7.5 Hz, 2H), 7.37-7.32 (m, 3H), 7.21 (t, *J* = 7.2 Hz, 1H), 7.14 – 7.08 (m, 2H), 7.08 – 7.00 (m, 5H), 6.93-6.89 (m, 3H), 5.06 (s, 1H), 3.70 (s, 3H), 3.18 (d, *J* = 13.4 Hz, 1H), 2.98-2.84 (m, 2H), 2.80 (d, *J* = 12.9 Hz, 1H). **^{13}C {^{1}H} NMR** (100 MHz, $CDCl_3$) δ 175.4, 157.0, 156.4, 153.5, 143.8, 137.3, 134.1, 130.3, 129.8, 128.5, 128.3, 127.9, 125.5, 125.4, 123.5, 121.4, 119.2, 118.5, 80.1, 53.4, 52.2, 45.9, 40.9, 37.6. **HRMS(ESI-TOF)** m/z: $[M+H^+]^+$ $C_{31}H_{27}O_4$ calcd. 463.1909, found. 463.1921.

Methyl-2-phenyl-5-(4-(trifluoromethoxy)phenyl)-2,3-dihydro-2,4-methanobenzo[b] oxepine-4(5H)-carboxylate(8)

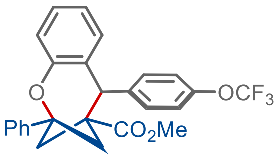

Following the general procedure(I), treatment of diol 1e (0.042 g, 0.15 mmol) and BCB

2a (0.020 g, 0.1 mmol) with $Yb(OTf)_3$ (0.003 g, 0.005 mmol) in THF:HFIP(7:3) (1.0 mL) at rt for 1 hr, delivered compound **8**, which was purified by silica gel column chromatography (EtOAc: Hexane 5:95) to furnish the title compound **8** as semi solid in 52% (0.025g) yield; **^{1}H NMR** (400 MHz, $CDCl_3$) δ 7.53 (d, *J* = 7.3 Hz, 2H), 7.44 (t, *J* = 7.5 Hz, 2H), 7.36 (t, *J* = 7.3 Hz, 1H), 7.24 – 7.20 (m, 1H), 7.10 (d, *J* = 4.9 Hz, 5H), 7.00 (d, *J* = 6.8 Hz, 1H), 6.92-6.88 (m, 1H), 5.09 (s, 1H), 3.69 (s, 3H), 3.17 (d, *J* = 13.5 Hz, 1H), 2.96 (dd, *J* = 13.1, 6.3 Hz, 1H), 2.81 (dd, *J* = 13.2, 4.4 Hz, 2H). **^{13}C {^{1}H} NMR** (100 MHz, $CDCl_3$) δ 175.2, 153.5, 148.3, 143.7, 141.3, 133.9, 130.5, 129.6, 128.6, 128.5, 128.0, 125.4, 125.0, 121.8, 121.6(2), 120.8, 119.3, 80.2, 53.3, 52.3, 45.8, 40.9, 37.6. **HRMS(ESI-TOF)** m/z: $[M+H^+]^+$ $C_{26}H_{22}F_3O_4$ calcd. 455.1470, found. 455.1461.

Methyl-5-(4-fluorophenyl)-2-phenyl-2,3-dihydro-2,4-methanobenzo[b]oxepine-4(5H)-carboxylate (9)

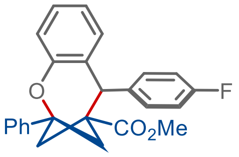

Following the general procedure(I), treatment of diol 1h (0.032 g, 0.15 mmol) and BCB 2a (0.020 g, 0.1 mmol) with $Yb(OTf)_3$ (0.003 g, 0.005 mmol) in THF:HFIP(7:3) (1.0 mL) at rt for 1 hr, delivered compound **9**, which was purified by silica gel column chromatography (EtOAc: Hexane 5:95) to furnish the title compound **9** as semi solid in 74% (0.032g) yield; **^{1}H NMR** (400 MHz, $CDCl_3$) δ 7.53 (d, *J* = 7.3 Hz, 2H), 7.43 (t, *J* = 7.5 Hz, 2H), 7.35 (t, *J* = 7.3 Hz, 1H), 7.22 – 7.17 (m, 1H), 7.09 (d, *J* = 8.2 Hz, 1H), 7.06-7.02 (m, 2H), 7.02 – 6.95 (m, 2H), 6.94 (d, *J* = 8.7 Hz, 1H), 6.90-6.87 (m, 1H), 5.04 (s, 1H), 3.69 (s, 3H), 3.15 (d, *J* = 13.5 Hz, 1H), 2.94 (dd, *J* = 13.0, 6.2 Hz, 1H), 2.86 – 2.76 (m, 2H). **^{13}C {^{1}H} NMR** (100 MHz, $CDCl_3$) δ 175.3, 163.1, 160.7, 153.5, 143.8, 138.4(2), 134.0, 130.62, 128.6, 128.4, 127.9, 125.4, 125.3, 121.7, 121.6, 115.4, 115.2, 80.1, 53.3, 52.3, 45.9, 40.9, 37.5. **HRMS(ESI-TOF)** m/z: $[M+H^+]^+$ $C_{25}H_{22}FO_3$ calcd. 389.1553, found. 389.1542.

Methyl-2-phenyl-5-(m-tolyl)-2,3-dihydro-2,4-methanobenzo[b]oxepine-4(5H)-carboxylate (10)

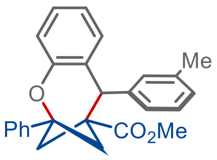

Following the general procedure(I), treatment of diol 1b (0.032 g, 0.15 mmol) and BCB 2a (0.020 g, 0.1 mmol) with $Yb(OTf)_3$ (0.003 g, 0.005 mmol) in THF:HFIP(7:3) (1.0 mL) at rt for 1 hr, delivered compound **10**, which was purified by silica gel column chromatography (EtOAc: Hexane 5:95) to furnish the title compound **10** as semi solid in 68% (0.028 g) yield; **^{1}H NMR** (400 MHz, $CDCl_3$) δ 7.53 (d, J = 7.2 Hz, 2H), 7.43 (t, J = 7.5 Hz, 2H), 7.35 (d, J = 7.2 Hz, 1H), 7.22 – 7.17 (m, 1H), 7.14 (d, J = 7.5 Hz, 1H), 7.09 (d, J = 8.2 Hz, 1H), 7.03 (d, J = 7.3 Hz, 2H), 6.90-6.84 (m, 3H), 5.01 (s, 1H), 3.68 (s, 3H), 3.13 (d, J = 13.3 Hz, 1H), 2.97-2.85 (m, 2H), 2.78 (d, J = 12.8 Hz, 1H), 2.29 (s, 3H). **^{13}C {^{1}H} NMR** (100 MHz, $CDCl_3$) δ 175.5, 153.6, 143.9, 142.5, 137.9, 134.2, 129.8, 128.5, 128.3, 128.2,127.9(2), 126.2, 125.5, 125.4, 121.4, 121.3, 80.1, 54.1, 52.1, 45.9, 40.9, 37.6, 21.6. **HRMS(ESI-TOF)** m/z: $[M+H^+]^+$ $C_{26}H_{25}O_3$ calcd. 385.1804, found. 385.1845.

Methyl-5-(3-methoxyphenyl)-2-phenyl-2,3-dihydro-2,4-methanobenzo[b]oxepine-4(5H)-carboxylate (11)

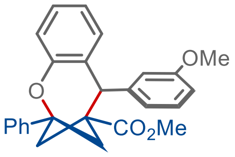

Following the general procedure(I), treatment of diol 1l (0.036 g, 0.15 mmol) and BCB 2a (0.020 g, 0.1 mmol) with $Yb(OTf)_3$ (0.003 g, 0.005 mmol) in THF:HFIP(7:3) (1.0 mL) at rt for 1 hr, delivered compound **11**, which was purified by silica gel column chromatography (EtOAc: Hexane 5:95) to furnish the title compound **11** as semi solid in 79% (0.035g) yield; **^{1}H NMR** (400 MHz, $CDCl_3$) δ 7.37 (d, *J* = 8.4 Hz, 2H), 7.29 (d, *J* = 8.4 Hz, 2H), 7.27 – 7.14 (m, 1H), 7.12-7.06 (m, 2H), 7.00 – 6.93 (m, 2H), 6.80 (t, *J* = 7.4 Hz, 1H), 6.67 (dd, *J* = 8.2, 1.8 Hz, 1H), 6.56 – 6.49 (m, 2H), 4.94 (s, 1H), 3.65 (s, 3H), 3.62 (s, 3H), 3.01 (d, *J* = 13.4 Hz, 1H), 2.84 (dd, *J* = 13.0, 6.0 Hz, 1H), 2.76

(dd, J = 13.4, 6.1 Hz, 1H), 2.63 (d, J = 12.9 Hz, 1H). **^{13}C NMR** (100 MHz, $CDCl_3$) δ 175.3, 159.6, 153.3, 144.0, 142.4, 134.1, 133.7, 129.4, 128.7, 128.3, 126.9, 125.2, 121.6, 121.5, 121.3, 115.3, 112.0, 79.6, 55.2, 53.9, 52.3, 45.7, 41.0, 37.8. **HRMS(ESI-TOF)** m/z: $[M+H^+]^+$ $C_{26}H_{25}O_4$ calcd. 401.1753, found. 401.1742.

Methyl-5-(3,5-dimethylphenyl)-2-phenyl-2,3-dihydro-2,4-methanobenzo[b]oxepine-4(5H)-carboxylate (12)

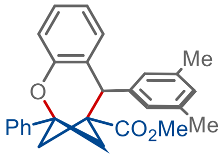

Following the general procedure(I), treatment of diol 1g (0.035 g, 0.15 mmol) and BCB 2a (0.020 g, 0.1 mmol) with $Yb(OTf)_3$ (0.003 g, 0.005 mmol) in THF:HFIP(7:3) (1.0 mL) at rt for 1 hr, delivered compound **12**, which was purified by silica gel column chromatography (EtOAc: Hexane 5:95) to furnish the title compound **12** as semi solid in 78% (0.0.033g) yield; **^{1}H NMR** (400 MHz, $CDCl_3$) δ 7.53 (d, J = 7.3 Hz, 2H), 7.42 (t, J = 7.5 Hz, 2H), 7.36-7.34 (m, 1H), 7.21 – 7.17 (m, 1H), 7.10-7.03 (m, 2H), 6.90-6.86 (m, 2H), 6.65 (s, 2H), 4.97 (s, 1H), 3.68 (s, 3H), 3.12 (d, J = 13.1 Hz, 1H), 2.96-2.86 (m, 2H), 2.77 (d, J = 12.8 Hz, 1H), 2.24 (s, 6H). **^{13}C {^{1}H} NMR** (100 MHz, $CDCl_3$) δ 175.6, 153.5, 144.0, 142.4, 137.8, 134.2, 128.8, 128.5, 128.1, 127.9, 126.9, 125.6, 125.4, 121.4, 125.3, 80.1, 54.1, 52.0, 45.9, 40.9, 37.7, 21.5. **HRMS(ESI-TOF)** m/z: $[M+H^+]^+$ $C_{27}H_{27}O_3$ calcd. 399.1960, found. 399.1948.

Methyl-2-phenyl-5-(thiophen-2-yl)-2,3-dihydro-2,4-methanobenzo[b]oxepine-4(5H)-carboxylate (13)

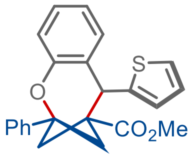

Following the general procedure(I), treatment of diol 1f (0.032 g, 0.15 mmol) and BCB 2a (0.020 g, 0.1 mmol) with $Yb(OTf)_3$ (0.003 g, 0.005 mmol) in THF:HFIP(7:3) (1.0 mL) at rt for 1 hr, delivered compound **13**, which was purified by silica gel column chromatography (EtOAc: Hexane 5:95) to furnish the title compound **13** as semi solid in 63% (0.030g) yield; **^{1}H NMR** (400 MHz, $CDCl_3$) δ 7.58 – 7.54 (m, 2H), 7.45 (t, J =

7.6 Hz, 2H), 7.38 (d, J = 7.1 Hz, 1H), 7.24 – 7.19 (m, 2H), 7.18 (d, J = 5.1 Hz, 1H), 7.09 (d, J = 8.2 Hz, 1H), 6.97 – 6.91 (m, 2H), 6.74 (d, J = 3.0 Hz, 1H), 5.39 (s, 1H), 3.78 (s, 3H), 3.31 (d, J = 12.5 Hz, 1H), 2.97 – 2.87 (m, 2H), 2.81 (d, J = 12.5 Hz, 1H). **^{13}C {^{1}H} NMR** (100 MHz, $CDCl_3$) δ 175.0, 152.5, 145.9, 143.7, 134.1, 128.6, 128.5, 127.9, 126.6, 126.5, 125.4, 124.9, 124.6, 121.5, 121.3, 80.2, 52.5, 49.3, 45.9, 40.4, 37.9. **HRMS(ESI-TOF)** m/z: [M+H$^+$] $^+$ $C_{23}H_{21}O_3S$ calcd. 377.1211, found. 377.1214.

methyl 5-(naphthalen-2-yl)-2-phenyl-2,3-dihydro-2,4-methanobenzo[b]oxepine-4(5H)-carboxylate (14)

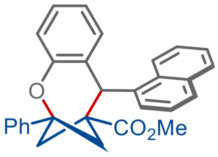

Following the general procedure(I), treatment of diol 1i (0.038 g, 0.15 mmol) and BCB 2a (0.020 g, 0.1 mmol) with $Yb(OTf)_3$ (0.003 g, 0.005 mmol) in THF:HFIP(7:3) (1.0 mL) at rt for 1 hr, delivered compound **14**, which was purified by silica gel column chromatography (EtOAc: Hexane 5:95) to furnish the title compound **14** as semi solid in 65% (0.030g) yield; **^{1}H NMR** (400 MHz, $CDCl_3$) δ 7.83 – 7.73 (m, 3H), 7.56 (d, J = 6.9 Hz, 3H), 7.48-7.42 (m 4H), 7.36 (t, J = 7.3 Hz, 1H), 7.25-7.18 (m, 2H), 7.17 – 7.13 (m, 1H), 7.06 (d, J = 7.1 Hz, 1H), 6.91 – 6.83 (m, 1H), 5.23 (s, 1H), 3.66 (s, 3H), 3.17 (d, J = 13.0 Hz, 1H), 3.05 – 2.95 (m, 2H), 2.85 (d, J = 12.6 Hz, 1H). **^{13}C {^{1}H} NMR** (100 MHz, $CDCl_3$) δ 175.4, 153.6, 143.9, 140.1, 134.2, 133.4, 132.6, 128.6, 128.3, 128.1(2), 128.0, 127.9, 127.7, 127.2, 126.2, 125.9, 125.4(2), 121.5(2), 80.2, 54.2, 52.2, 46.1, 41.1, 37.7. **HRMS(ESI-TOF)** m/z: [M+H$^+$] $^+$ $C_{29}H_{25}O_3$ calcd. 421.1804, found. 421.1795.

Methyl-5-methyl-2-phenyl-2,3-dihydro-2,4-methanobenzo[b]oxepine-4(5H)-carboxylate (15)

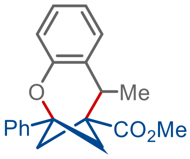

Following the general procedure(I), treatment of diol 1o (0.020 g, 0.15 mmol) and BCB 2a (0.020g, 0.1 mmol) with $Yb(OTf)_3$ (0.003 g, 0.005 mmol) in THF:HFIP(7:3) (1.0 mL)

at rt for 1 hr, delivered compound **15**, which was purified by silica gel column chromatography (EtOAc: Hexane 5:95) to furnish the title compound **15** as semi solid in 62% (0.020g) yield; ^{1}**H NMR** (400 MHz, $CDCl_3$) δ 7.54 (d, *J* = 7.2 Hz, 2H), 7.42 (t, *J* = 7.5 Hz, 2H), 7.35-7.27 (m, 2H), 7.17 (dd, *J* = 11.1, 4.1 Hz, 1H), 7.02-6.96 (m, 2H), 3.80 – 3.73 (m, 4H), 3.30 (d, *J* = 13.2 Hz, 1H), 2.82 – 2.65 (m, 3H), 1.30 (d, *J* = 6.9 Hz, 3H). ^{13}**C {^{1}H} NMR** (100 MHz, $CDCl_3$) δ 176.0, 151.9, 144.1, 132.5, 128.5, 127.8, 127.4, 125.4, 121.4,121.3, 80.0, 52.5, 44.7, 42.0, 40.5, 37.8, 20.0. **HRMS(ESI-TOF)** m/z: $[M+H^+]^+$ $C_{20}H_{21}O_3$ calcd. 309.1467, found. 309.1491.

Methyl-7-(tert-butyl)-2,5-diphenyl-2,3-dihydro-2,4-methanobenzo[b]oxepine-4(5H)-carboxylate (16)

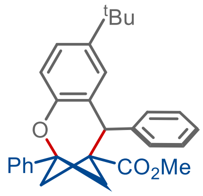

Following the general procedure(I), treatment of diol 1m (0.038 g, 0.15 mmol) and BCB 2a (0.020 g, 0.1 mmol) with $Yb(OTf)_3$ (0.003 g, 0.005 mmol) in THF:HFIP(7:3) (1.0 mL) at rt for 1 hr, delivered compound **16**, which was purified by silica gel column chromatography (EtOAc: Hexane 5:95) to furnish the title compound **16** as semi solid in 71% (0.034g) yield; ^{1}**H NMR** (400 MHz, $CDCl_3$) δ 7.52 – 7.48 (m, 2H), 7.40 (t, *J* = 7.5 Hz, 2H), 7.32 (t, *J* = 7.3 Hz, 1H), 7.25 – 7.18 (m, 4H), 7.05-7.00 (m, 3H), 6.97 (d, *J* = 2.3 Hz, 1H), 5.03 (s, 1H), 3.67 (s, 3H), 3.11 (d, *J* = 13.4 Hz, 1H), 2.95 (dd, *J* = 12.9, 6.2 Hz, 1H), 2.82 (dd, *J* = 13.4, 6.2 Hz, 1H), 2.75 (d, *J* = 12.9 Hz, 1H), 1.17 (s, 9H). ^{13}**C {^{1}H} NMR** (100 MHz, $CDCl_3$) δ 175.6, 151.4, 144.0, 143.9, 142.7, 130.6, 129.1, 128.5, 128.3, 127.8, 127.0, 125.5,125.4, 124.6, 120.8, 79.9, 54.2, 52.1, 45.9, 41.0, 37.5, 34.0, 31.5. **HRMS(ESI-TOF)** m/z: $[M+H^+]^+$ $C_{29}H_{31}O_3$ calcd. 427.2273, found 427.2270.

Methyl-7-methoxy-2,5-diphenyl-2,3-dihydro-2,4-methanobenzo[b]oxepine-4(5H)-carboxylate (17)

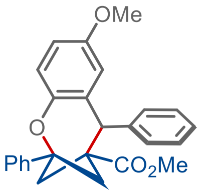

Following the general procedure(I), treatment of diol 1n (0.036 g, 0.15 mmol) and BCB 2a (0.020 g, 0.1 mmol) with $Yb(OTf)_3$ (0.003 g, 0.005 mmol) in THF:HFIP(7:3) (1.0 mL) at rt for 1 hr, delivered compound **17**, which was purified by silica gel column chromatography (EtOAc: Hexane 5:95) to furnish the title compound **17** as semi solid in 67% (0.0.29g) yield; **^{1}H NMR** (400 MHz, $CDCl_3$) δ 7.53 (d, *J* = 7.5 Hz, 2H), 7.43 (t, *J* = 7.4 Hz, 2H), 7.35 (t, *J* = 7.1 Hz, 1H), 7.29-7.25 (m, 3H), 7.09-7.09 (m, 3H), (m, 6.82 (dd, *J* = 8.8, 2.2 Hz, 1H), 6.53 (d, *J* = 1.9 Hz, 1H), 5.03 (s, 1H), 3.70 (s, 3H), 3.66 (s, 3H), 3.14 (d, *J* = 13.4 Hz, 1H), 2.96 (dd, *J* = 12.9, 6.1 Hz, 1H), 2.83 (dd, *J* = 13.3, 6.1 Hz, 1H), 2.77 (d, *J* = 12.9 Hz, 1H). **^{13}C {^{1}H} NMR** (100 MHz, $CDCl_3$) δ 175.5, 153.9, 147.9, 144.0, 142.2, 129.1, 128.5,128.4, 127.8, 127.1, 126.3, 125.4, 122.3, 117.3, 115.2, 79.9, 55.7, 54.2, 52.2, 45.9, 40.8, 37.7. **HRMS(ESI-TOF)** m/z: [M+H$^+$] $C_{26}H_{25}O_4$ calcd. 401.1753, found. 401.1742.

Ethyl 2,5-diphenyl-2,3-dihydro-2,4-methanobenzo[b]oxepine-4(5H)-carboxylate(18)

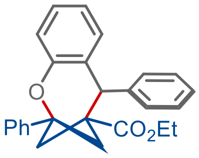

Following the general procedure(I), treatment of diol 1a (0.033g, 0.15 mmol) and BCB 2b (0.021 g, 0.1 mmol) with $Yb(OTf)_3$ (0.003 g, 0.005 mmol) in THF:HFIP(7:3) (1.0 mL) at rt for 1 hr, delivered compound **18**, which was purified by silica gel column chromatography (EtOAc: Hexane 5:95) to furnish the title compound **18** as semi solid in 63% (0.025 g) yield; **^{1}H NMR** (400 MHz, $CDCl_3$) δ 7.55 (d, *J* = 7.4 Hz, 2H), 7.45 (t, *J* = 7.6 Hz, 2H), 7.37 (t, *J* = 7.3 Hz, 1H), 7.31 – 7.26 (m, 2H), 7.24 - 7.20 (m, 2H), 7.11 (d, *J* = 8.1 Hz, 3H), 7.05 (d, *J* = 7.7 Hz, 1H), 6.90 (t, *J* = 7.4 Hz, 1H), 5.07 (s, 1H), 4.15 (q, *J* = 7.1 Hz, 2H), 3.16 (d, *J* = 13.4 Hz, 1H), 2.97 (dd, *J* = 12.9, 6.1 Hz, 1H), 2.90 (dd,

J = 13.4, 6.1 Hz, 1H), 2.80 (d, J = 12.9 Hz, 1H), 1.24 (t, J = 7.1 Hz, 3H). **^{13}C {^{1}H} NMR** (100 MHz, $CDCl_3$) δ 175.0, 153.6, 144.0, 142.6, 134.1, 129.2, 128.6, 128.4, 128.2, 127.9, 127.1, 125.6, 125.4, 121.4, 80.1, 61.2, 54.1, 45.8, 41.0, 37.6, 14.3. **HRMS(ESI-TOF)** m/z: $[M+H^+]^+$ $C_{26}H_{25}O_3$ calcd. 385.1790, found. 385.1804.

Benzyl 2,5-diphenyl-2,3-dihydro-2,4-methanobenzo[b]oxepine-4(5H)-carboxylate (19)

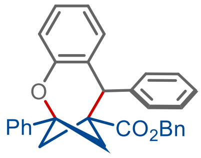

Following the general procedure(I), treatment of diol 1a (0.033 g, 0.15 mmol) and BCB 2c (0.027 g, 0.1 mmol) with $Yb(OTf)_3$ (0.003 g, 0.005 mmol) in THF:HFIP(7:3) (1.0 mL) at rt for 1 hr, delivered compound **19**, which was purified by silica gel column chromatography (EtOAc: Hexane 5:95) to furnish the title compound **19** as semi solid in 69% (0.032g) yield; **^{1}H NMR** (400 MHz, $CDCl_3$) δ 7.53 (d, J = 7.3 Hz, 2H), 7.42 (t, J = 7.5 Hz, 2H), 7.40 – 7.35 (m, 4H), 7.32 – 7.27 (m, 2H), 7.22 – 7.16 (m, 4H), 7.09 (d, J = 7.6 Hz, 1H), 7.02-6.99 (m, 3H), 6.89-6.85 (m, 1H), 5.14 (t, J = 12.0 Hz, 2H), 5.06 (s, 1H), 3.17 (d, J = 13.4 Hz, 1H), 2.97 (dd, J = 12.9, 6.1 Hz, 1H), 2.89 (dd, J = 13.4, 6.1 Hz, 1H), 2.80 (d, J = 12.8 Hz, 1H). **^{13}C {^{1}H} NMR** (100 MHz, $CDCl_3$) δ 174.8, 153.6, 143.9, 142.5, 135.6, 134.1, 129.2, 128.7, 128.6, 128.5, 128.4, 128.2, 127.9, 127.1, 125.5, 125.4, 121.4, 80.1, 67.0, 54.0, 45.9, 41.0, 37.7. **HRMS(ESI-TOF)** m/z: $[M+H^+]^+$ $C_{31}H_{27}O_3$ calcd. 447.1960, found. 447.1944.

Methyl 2,5-bis(4-(tert-butyl)phenyl)-2,3-dihydro-2,4-methanobenzo[b]oxepine-4(5H)-carboxylate (20).

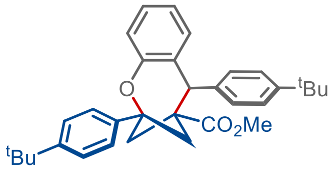

Following the general procedure(I), treatment of diol 1c (0.038 g, 0.15 mmol) and BCB 2e (0.032 g, 0.1 mmol) with $Yb(OTf)_3$ (0.003 g, 0.005 mmol) in THF:HFIP(7:3) (1.0 mL) at rt for 1 hr, delivered compound **20**, which was purified by silica gel column chromatography (EtOAc: Hexane 5:95) to furnish the title compound **20** as semi solid

in 80% (0.039g) yield; **^{1}H NMR** (600 MHz, Chloroform-*d*) δ 7.45 (d, *J* = 1.8 Hz, 4H), 7.26 (t, *J* = 2.5 Hz, 1H), 7.24 (d, *J* = 1.9 Hz, 1H), 7.20 – 7.16 (m, 1H), 7.08 – 7.05 (m, 1H), 7.04 (dd, *J* = 7.9, 1.7 Hz, 1H), 6.98 – 6.95 (m, 2H), 6.89 – 6.84 (m, 1H), 5.02 (s, 1H), 3.67 (s, 3H), 3.17 – 3.10 (m, 1H), 2.93 (dd, *J* = 12.8, 6.2 Hz, 1H), 2.86 (dd, *J* = 13.3, 6.2 Hz, 1H), 2.77 (d, *J* = 12.9 Hz, 1H), 1.36 (s, 9H), 1.29 (s, 9H). **^{13}C {^{1}H} NMR** (150 MHz, Chloroform-*d*) δ 175.6, 153.7, 150.8, 149.7, 141.1, 139.5, 134.2, 128.7, 128.1, 126.1, 125.8, 125.5, 125.2, 121.4, 121.3, 80.0, 53.6, 52.1, 45.9, 40.9, 37.6, 34.7, 34.5, 31.5. **HRMS(ESI-TOF)** m/z: [M+H$^+$] $^+$ $C_{33}H_{39}O_3$ calcd. 483.2894, found. 483.2909.

Methyl 2-(4-fluorophenyl)-5-phenyl-2,3-dihydro-2,4-methanobenzo[b]oxepine-4(5H)-carboxylate (21)

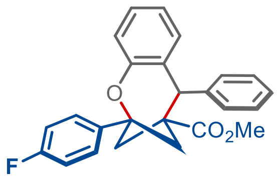

Following the general procedure(I), treatment of diol 1a (0.033 g, 0.15 mmol) and BCB 2f (0.021 g, 0.1 mmol) with $Yb(OTf)_3$ (0.003 g, 0.005 mmol) in THF:HFIP(7:3) (1.0 mL) at rt for 1 hr, delivered compound **21**, which was purified by silica gel column chromatography (EtOAc: Hexane 5:95) to furnish the title compound **21** as semi solid in 58% (0.025g) yield; **^{1}H NMR** (400 MHz, $CDCl_3$) δ 7.55 – 7.48 (m, 2H), 7.31 – 7.20 (m, 5H), 7.16 – 7.11 (m, 2H), 7.08 (d, *J* = 6.8 Hz, 3H), 6.92-6.89 (m, 1H), 5.07 (s, 1H), 3.71 (s, 3H), 3.12 (d, *J* = 13.5 Hz, 1H), 2.97 (dd, *J* = 12.9, 6.1 Hz, 1H), 2.88 (dd, *J* = 13.4, 6.2 Hz, 1H), 2.77 (d, *J* = 12.9 Hz, 1H). **^{13}C {^{1}H} NMR** (100 MHz, $CDCl_3$) δ 175.3, 163.6, 161.2, 153.4, 142.5, 139.9, 134.2, 129.1, 128.5, 128.3, 127.3, 127.2, 125.4, 121.5, 121.3, 115.4, 115.2, 79.7, 54.0, 52.2, 45.8, 41.0, 37.7. **HRMS(ESI-TOF)** m/z: [M+H$^+$] $^+$ $C_{25}H_{22}FO_3$ calcd. 389.1553, found. 389.1558.

methyl 2-(4-chlorophenyl)-5-phenyl-2,3-dihydro-2,4-methanobenzo[b]oxepine-4(5H)-carboxylate (22)

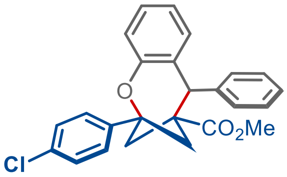

Following the general procedure(I), treatment of diol 1a (0.033g, 0.15 mmol) and BCB 2g (0.022 g, 0.1 mmol) with $Yb(OTf)_3$ (0.003 g, 0.005 mmol) in THF:HFIP(7:3) (1.0 mL) at rt for 1 hr, delivered compound **22**, which was purified by silica gel column chromatography (EtOAc: Hexane 5:95) to furnish the title compound **22** as semi solid in 67% (0.028g) yield; **^{1}H NMR** (400 MHz, $CDCl_3$) δ 7.48 (d, *J* = 8.6 Hz, 2H), 7.40 (d, *J* = 8.5 Hz, 2H), 7.30-7.26 (m, 2H), 7.25 – 7.19 (m, 2H), 7.11 – 7.02 (m, 4H), 6.90 (t, *J* = 7.4 Hz, 1H), 5.07 (s, 1H), 3.71 (s, 3H), 3.11 (d, *J* = 13.4 Hz, 1H), 2.95 (dd, *J* = 12.9, 6.2 Hz, 1H), 2.87 (dd, *J* = 13.4, 6.2 Hz, 1H), 2.76 (d, *J* = 12.9 Hz, 1H). **^{13}C {^{1}H} NMR** (100 MHz, $CDCl_3$) δ 175.3, 153.3, 142.4(2), 134.1, 133.7, 129.1, 128.6, 128.5, 128.3, 127.2, 126.9, 125.4, 121.6, 121.3, 79.6, 54.0, 52.2, 45.8, 41.0, 37.7. **HRMS(ESI-TOF)** m/z: $[M+H^+]^+$ $C_{25}H_{22}ClO_3$ calcd. 405.1257, found. 405.1246.

methyl 2-(4-bromophenyl)-5-phenyl-2,3-dihydro-2,4-methanobenzo[b]oxepine-4(5H)-carboxylate (23)

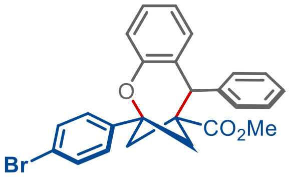

Following the general procedure(I), treatment of diol 1a (0.033 g, 0.15 mmol) and BCB 2h (0.027g, 0.1 mmol) with $Yb(OTf)_3$ (0.003 g, 0.005 mmol) in THF:HFIP(7:3) (1.0 mL) at rt for 1 hr, delivered compound **23**, which was purified by silica gel column chromatography (EtOAc: Hexane 5:95) to furnish the title compound **23** as semi solid in 64% (0.032g) yield; **^{1}H NMR** (400 MHz, $CDCl_3$) δ 7.56 (d, *J* = 8.5 Hz, 2H), 7.42 (d, *J* = 8.5 Hz, 2H), 7.30 – 7.24 (m, 3H), 7.23 – 7.19 (m, 1H), 7.11 – 7.02 (m, 4H), 6.90 (t, *J* = 7.4 Hz, 1H), 5.07 (s, 1H), 3.71 (s, 3H), 3.10 (d, *J* = 13.4 Hz, 1H), 2.95 (dd, *J* = 12.9, 6.1 Hz, 1H), 2.87 (dd, *J* = 13.4, 6.1 Hz, 1H), 2.75 (d, *J* = 12.9 Hz, 1H). **^{13}C {^{1}H} NMR**

(101 MHz, $CDCl_3$) δ 175.2, 153.3, 142.9, 142.4, 134.1, 131.6, 129.1, 128.5, 128.3, 127.3, 127.2, 125.4, 121.9, 121.6, 121.3, 79.6, 54.0, 52.2, 45.8, 40.9, 37.7. **HRMS(ESI-TOF)** m/z: $[M+H^+]^+$ $C_{25}H_{22}BrO_3$ calcd. 449.0752, found. 449.0732.

Methyl 5-phenyl-2-(m-tolyl)-2,3-dihydro-2,4-methanobenzo[b]oxepine-4(5H)-carboxylate (24)

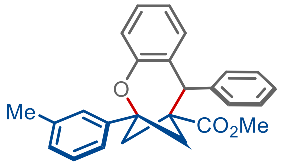

Following the general procedure(I), treatment of diol 1a (0.033 g, 0.15 mmol) and BCB 2d (0.021 g, 0.1 mmol) with $Yb(OTf)_3$ (0.003 g, 0.005 mmol) in THF:HFIP(7:3) (1.0 mL) at rt for 1 hr, delivered compound **24**, which was purified by silica gel column chromatography (EtOAc: Hexane 5:95) to furnish the title compound **24** as semi solid in 61% (0.026 g) yield; **^{1}H NMR** (400 MHz, $CDCl_3$) δ 7.33 (d, J = 5.1 Hz, 3H), 7.28 (d, J = 2.4 Hz, 1H), 7.26 – 7.22 (m, 2H), 7.21-7.17 (m,, 2H), 7.12-7.07 (m, 2H), 7.08 – 7.02 (m, 2H), 6.91-6.87 (m, 1H), 5.06 (s, 1H), 3.69 (s, 3H), 3.14 (d, J = 13.3 Hz, 1H), 2.96 (dd, J = 12.9, 6.1 Hz, 1H), 2.88 (dd, J = 13.4, 6.1 Hz, 1H), 2.80 (d, J = 12.9 Hz, 1H), 2.43 (s, 3H). **^{13}C {^{1}H} NMR** (100 MHz, $CDCl_3$) δ 175.5, 153.6, 143.9, 142.6, 138.2, 134.1, 129.1, 128.6, 128.5, 128.4, 128.2, 127.1, 126.2, 125.5, 122.5, 121.4(2), 80.1, 54.1, 52.2, 45.9, 40.8, 37.6, 21.7. **HRMS(ESI-TOF)** m/z: $[M+H^+]^+$ $C_{26}H_{25}O_3$ calcd. 385.1804, found. 385.1779.

Methyl 2-(3-bromophenyl)-5-phenyl-2,3-dihydro-2,4-methanobenzo[b]oxepine-4(5H)-carboxylate (25)

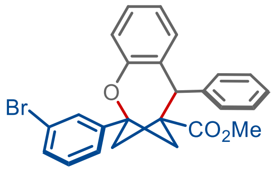

Following the general procedure(I), treatment of diol 1a (0.033 g, 0.15 mmol) and BCB 2i (0.027 g, 0.1 mmol) with $Yb(OTf)_3$ (0.003 g, 0.005 mmol) in THF:HFIP(7:3) (1.0 mL) at rt for 1 hr, delivered compound **25**, which was purified by silica gel column

chromatography (EtOAc: Hexane 5:95) to furnish the title compound **25** as semi solid in 67% (0.032g) yield; **^{1}H NMR** (400 MHz, $CDCl_3$) δ 7.71 (s, 1H), 7.52 – 7.44 (m, 2H), 7.34-7.29 (m, 2H), 7.27-7.21 (m, 3H), 7.12 (d, *J* = 8.2 Hz, 1H), 7.06 (t, *J* = 7.5 Hz, 3H), 6.91 (t, *J* = 7.4 Hz, 1H), 5.07 (s, 1H), 3.72 (s, 3H), 3.11 (d, *J* = 13.4 Hz, 1H), 2.95 (dd, *J* = 12.9, 6.2 Hz, 1H), 2.87 (dd, *J* = 13.5, 6.2 Hz, 1H), 2.77 (d, *J* = 12.9 Hz, 1H). **^{13}C {^{1}H} NMR** (100 MHz, $CDCl_3$) δ 175.2, 153.3, 146.0, 142.4, 134.1, 131.0, 130.2, 129.1, 128., 128.5,128.3, 127.2, 125.4, 124.1, 122.7, 121.7, 121.3, 79.5, 54.0, 52.3, 45.9, 40.9, 37.8. **HRMS(ESI-TOF)** m/z: [M+Na$^+$] $^+$ $C_{25}H_{21}BrNaO_3$ calcd. 471.0537, found. 471.0572.

7. General Procedure for the HFIP-mediated (4+2) Annulation of BCBs with diols

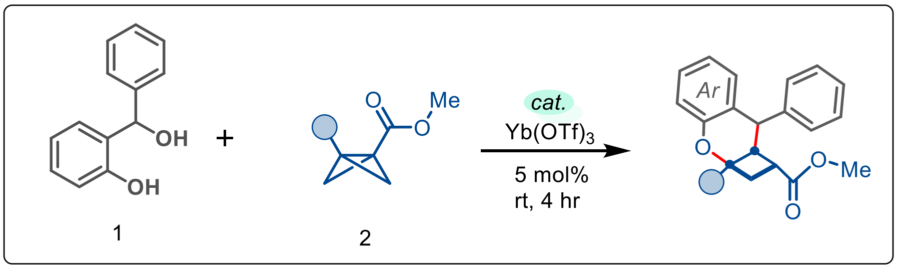

General Procedure (II):

An oven-dried screw-capped test tube equipped with a magnetic stir bar was charged bicyclo[1.1.0]butane derivative **2** (0.10 mmol, 1.0 equiv.) under a nitrogen atmosphere. Anhydrous THF (THF 0.7 mL) was added to dissolve the reactant, followed by the addition of hexafluoroisopropanol (HFIP, 0.3 mL) at rt, followed by the addition of $Yb(OTf)_3$ (0.005 mmol), the reaction mixture was stirred for 1 hour. After that Diol **1** (0.15 mmol, 1.5 equiv.) was added to this rection mixture and stirred for additional 3 hours. Upon completion, the reaction mixture was concentrated under reduced pressure. The resulting crude residue was pre-adsorbed onto silica gel and purified by flash column chromatography (petroleum ether/ethyl acetate as eluent) to afford fused product in good to excellent yields.

7.1 Reaction with isolated cyclobutene *(procedure same as above)*

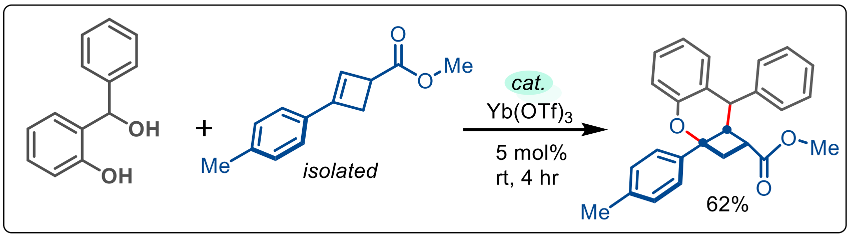

8. Experimental Details for the Synthesized Compounds:(26-40)

Methyl-8-phenyl-2a-(p-tolyl)-2,2a,8,8a-tetrahydro-1H-cyclobuta[b]chromene-1-carboxylate (26)

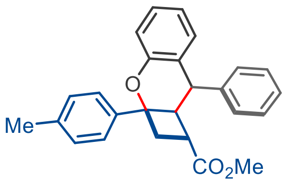

Following the general procedure(II), treatment of BCB 2j (0.021 g, 0.1 mmol) and diol **1a** (0.033 g, 0.15 mmol) with $Yb(OTf)_3$ (0.003 g, 0.005 mmol) in THF:HFIP(7:3) (1.0 mL) at rt for 4 h, delivered compound **26**, which was purified by silica gel column chromatography (EtOAc: Hexane 5:95) to furnish the title compound **26** as semi solid in 56% (0.027g) yield (dr 2.7:1); **^{1}H NMR** (400 MHz, $CDCl_3$) δ 7.46 (d, J = 8.2 Hz, 2H), 7.32 (t, J = 7.2 Hz, 2.12H), 7.29 – 7.22 (m, 3.5H), 7.21 – 7.15 (m, 4.39H), 7.14 – 7.06 (m, 2H), 7.05 – 7.00 (m, 1H), 6.97 (d, J = 7.6 Hz, 1.3H), 6.92 – 6.87 (m, 1H), 4.26 (d, J = 6.6 Hz, 1H), 4.21 (d, J = 1.4 Hz, 0.4H), 3.75 (s, 1H), 3.68 (d, J = 8.3 Hz, 0.4H), 3.63 (dd, J = 9.4, 6.6 Hz, 1H), 3.37 (s, 3H), 3.14 (q, J = 8.9 Hz, 1H), 3.02 (dd, J = 18.2, 8.4 Hz, 0.4H), 2.82 (dd, J = 12.9, 8.4 Hz, 0.4H), 2.74 – 2.68 (m, 0.4H), 2.66 (d, J = 8.8 Hz, 2H), 2.37 (s, 3H), 2.30 (s, 1H). **^{13}C {^{1}H} NMR** (100 MHz, $CDCl_3$) δ 174.6, 154.4, 140.4, 140.0, 137.5, 129.8, 129.3, 128.5, 128.1, 126.9, 125.6, 125.4, 123.4, 121.1, 117.7, 80.5, 51.7, 49.6, 40.1, 38.1, 36.1, 21.2. **HRMS(ESI-TOF)** m/z: $[M+H^+]^+$ $C_{26}H_{25}O_3$ calcd. 385.1804, found. 385.1786.

Methyl-2a-(4-(tert-butyl)phenyl)-8-phenyl-2,2a,8,8a-tetrahydro-1H-cyclobuta[b]chromene-1-carboxylate (27)

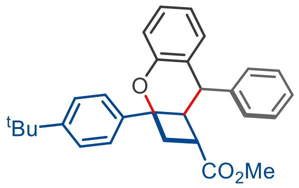

Following the general procedure(II), treatment of BCB 2e (0.025 g, 0.1 mmol) and diol **1a** (0.031 g, 0.15 mmol) with $Yb(OTf)_3$ (0.003 g, 0.005 mmol) in THF:HFIP(7:3) (1.0 mL) at rt for 4 h, delivered compound **27**, which was purified by silica gel column chromatography (EtOAc: Hexane 5:95) to furnish the title compound **27** as semi solid

in 67% (0.031g) yield (dr 3:1); **^{1}H NMR** (400 MHz, $CDCl_3$) δ 7.51-7.47 (m, 2H), 7.43 (d, *J* = 8.5 Hz, 2H), 7.31 (t, *J* = 7.2 Hz, 2H), 7.27 – 7.23 (m, 3H), 7.20 – 7.15 (m, 3.5H), 7.12-7.09 (m, 2H), 7.01 – 6.95 (m, 2H), 6.89 (t, *J* = 7.3 Hz, 1H), 4.26 (d, *J* = 6.5 Hz, 1H), 4.21 (s, 0.4H), 3.74 (s, 1H), 3.69-3.67 (m, 0.4H), 3.63 (dd, *J* = 9.4, 6.6 Hz, 1H), 3.36 (s, 3H), 3.14 (q, *J* = 8.9 Hz, 1H), 3.05 – 2.98 (m, 0.4H), 2.86 – 2.81 (m, 0.4H), 2.72 – 2.68 (m, 0.4H), 2.65 (dd, *J* = 8.7, 3.8 Hz, 2H), 1.34 (s, 9H), 1.28 (s, 3.2H). **^{13}C {^{1}H} NMR** (100 MHz, $CDCl_3$) δ 174.6, 154.5, 150.7, 140.4, 139.9, 129.8, 128.5, 128.3, 128.1, 126.9, 125.5, 125.2, 123.4, 121.1, 117.7, 80., 51.6, 49.6, 40.1, 38.1, 36.1, 34.6, 31.4. **HRMS(ESI-TOF)** m/z: $[M+H^+]^+$ $C_{29}H_{31}O_3$ calcd. 427.2251, found. 427.2273.

Methyl-8-(4-fluorophenyl)-2a-(p-tolyl)-2,2a,8,8a-tetrahydro-1H-cyclobuta[b]chromene-1-carboxylate (28)

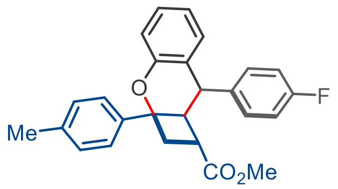

Following the general procedure(II), treatment of BCB 2j (0.021 g, 0.1 mmol) and diol **1h** (0.034 g, 0.15 mmol) with $Yb(OTf)_3$ (0.003 g, 0.005 mmol) in THF:HFIP(7:3) (1.0 mL) at rt for 4 h, delivered compound **28**, which was purified by silica gel column chromatography (EtOAc: Hexane 5:95) to furnish the title compound **28,** as semi solid in 63% (0.028g) yield (dr 2.2:1); **^{1}H NMR** (400 MHz, DMSO-d_6) δ 7.36 (d, *J* = 8.1 Hz, 2H), 7.29 – 7.24 (m, 0.6H), 7.21 – 7.16 (m, 4.9H), 7.12-7.07 (m, 4.5H), 7.02 – 6.96 (m, 3H), 6.90 – 6.85 (m, 1H), 6.82 (d, *J* = 7.3 Hz, 1H), 4.43 (d, *J* = 7.3 Hz, 1H), 4.25 (d, *J* = 2.2 Hz, 0.4H), 3.64 (s, 1.3H), 3.525-3.50 (m, 1.5H), 3.28 (s, 3H), 2.95 – 2.83 (m, 1.4H), 2.68 – 2.52 (m, 2H), 2.41 (dd, *J* = 11.6, 9.2 Hz, 1H), 2.28 (s, 3H), 2.23 (s, 1H). **^{13}C {^{1}H} NMR** (101 MHz, DMSO-d_6) δ 173.5, 153.7, 139.4, 137.2, 129.4, 129.1, 128.8, 128.1, 125.2, 125.1, 121.3, 117.7, 115.2, 115.0, 80.0, 51.4, 47.7, 38.2, 37.0, 35.7, 20.7. **HRMS(ESI-TOF)** m/z: $[M+Na^+]^+$ $C_{26}H_{23}FNaO_3$ calcd. 425.1523, found. 425.1516.

methyl-(4-(tert-butyl)phenyl)-8-(4-fluorophenyl)-2,2a,8,8a-tetrahydro-1H-cyclobuta[b]chromene-1-carboxylate (29)

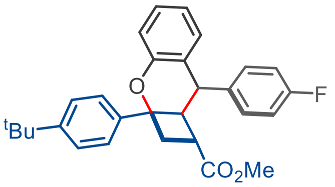

Following the general procedure (II), treatment of BCB 2e (0.025 g, 0.1 mmol) and diol **1h** (0.035 g, 0.15 mmol) with Yb(OTf)$_3$ (0.003 g, 0.005 mmol) in THF:HFIP(7:3) (1.0 mL) at rt for 4 h, delivered compound **29**, which was purified by silica gel column chromatography (EtOAc: Hexane 5:95) to furnish the title compound **29** as semi solid in 63% (0.030g) yield (dr 10:1); **^{1}H NMR** (400 MHz, CDCl$_3$) δ 7.48 – 7.36 (m, 5H), 7.31 (d, *J* = 8.4 Hz, 1H), 7.25-7.20 (m, 1H), 7.16-7.12 (m, 1H), 6.99 (t, *J* = 8.7 Hz, 2H), 6.93 – 6.86 (m, 2H), 4.23 (d, *J* = 6.6 Hz, 1H), 3.58 (dd, *J* = 9.4, 6.6 Hz, 1H), 3.39 (s, 3H), 3.10 – 3.02 (m, 1H), 2.69-2.59 (m, 2H), 1.32 (s, 9H). **^{13}C {^{1}H} NMR** (101 MHz, CDCl$_3$) δ 174.8, 163.2, 160.7, 154.5, 150.8, 139.8, 136.2, 129.6, 128.3, 125.5, 125.4, 125.1, 124.6, 123.7, 123.2, 121.2, 117.9, 115.5, 115.3, 80.4, 51.9, 49.6, 41.5, 39.4, 38.0, 34.7, 32.8, 31.4(2). **HRMS(ESI-TOF)** m/z: [M+Na$^+$] $^+$ $C_{29}H_{29}FNaO_3$ calcd. 467.1998, found. 467.1997.

Methyl-8-(4-methoxyphenyl)-2a-(p-tolyl)-2,2a,8,8a-tetrahydro-1H-cyclobuta[b]chromene-1-carboxylate (30)

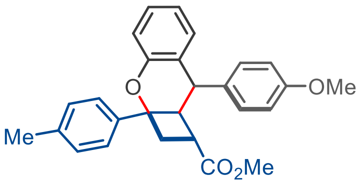

Following the general procedure(II), treatment of BCB 2j (0.021 g, 0.1 mmol) and diol **1p** (0.034 g, 0.15 mmol) with Yb(OTf)$_3$ (0.003 g, 0.005 mmol) in THF:HFIP(7:3) (1.0 mL) at rt for 4 h, delivered compound **30**, which was purified by silica gel column chromatography (EtOAc: Hexane 5:95) to furnish the title compound **30** as semi solid in 58% (0.027g) yield (dr 3:1); **^{1}H NMR** (400 MHz, DMSO-d$_6$) δ 7.36 (d, *J* = 8.1 Hz, 2H), 7.20 (d, *J* = 8.1 Hz, 3.6H), 7.13 – 7.04 (m, 3.5H), 7.01-6.94 (m, 2.3H), 6.91 – 6.87 (m, 2H), 6.85 – 6.81 (m, 2H), 6.71 (d, *J* = 8.7 Hz, 1H), 4.30 (d, *J* = 6.9 Hz, 1H), 4.15 (d, *J* = 2.1 Hz, 0.3H), 3.72 (s, 3H), 3.64 (s, 1H), 3.63 (s, 1H), 3.51-3.48 (m, 1.4H), 3.29

(s, 3H), 2.94-2.84 (m, 1.3H), 2.62 – 2.52 (m, 1.7H), 2.46 – 2.40 (m, 1H), 2.28 (s, 3H), 2.24 (s, 1H). **^{13}C {^{1}H} NMR** (101 MHz, DMSO-d$_6$) δ 153.7, 132.3, 129.25, 128.8, 128.0, 125.2, 121.2, 117.6, 113.8, 80.1, 55.1, 51.5, 48.4, 38.3, 37.1, 35.6, 20.7. **HRMS(ESI-TOF)** m/z: [M+Na$^+$] $^+$ $C_{27}H_{26}NaO_4$ calcd. 437.1729, found. 437.1764.

Methyl-8-([1,1'-biphenyl]-4-yl)-2a-(p-tolyl)-2,2a,8,8a-tetrahydro-1H-cyclobuta[b]chromene-1-carboxylate (31)

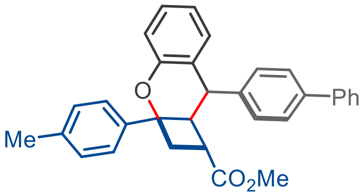

Following the general procedure(II), treatment of BCB 2j (0.021 g, 0.1 mmol) and diol **1j** (0.042 g, 0.15 mmol) with Yb(OTf)$_3$ (0.003 g, 0.005 mmol) in THF:HFIP(7:3) (1.0 mL) at rt for 4 h, delivered compound **31**, which was purified by silica gel column chromatography (EtOAc: Hexane 5:95) to furnish the title compound **31** as semi solid in 58% (0.029g) yield (dr 2.2:1); **^{1}H NMR** (400 MHz, CDCl$_3$) δ 7.61 (d, *J* = 7.3 Hz, 2H), 7.57-7.52 (m, 3H), 7.46 (t, *J* = 7.5 Hz, 4H), 7.42 – 7.38 (m, 2H), 7.37 – 7.32 (m, 1H), 7.32 – 7.27 (m, 1.4H), 7.26 – 7.20 (m, 4.8 H), 7.14 (dd, *J* = 15.6, 8.0 Hz, 2H), 7.10 – 6.98 (m, 3.5H), 6.92 (t, *J* = 7.4 Hz, 1H), 4.31 (d, *J* = 6.6 Hz, 1H), 4.25 (s, 0.5H), 3.76 (s, 1.4H), 3.72 (d, *J* = 8.1 Hz, 0.5H), 3.65 (dd, *J* = 9.4, 6.7 Hz, 1H), 3.34 (s, 3H), 3.17 (q, *J* = 8.9 Hz, 1H), 3.04 (dd, *J* = 18.1, 8.4 Hz, 0.5H), 2.83 (dd, *J* = 12.9, 8.4 Hz, 0.5H), 2.75 – 2.70 (m, 0.5H), 2.67 (dd, *J* = 8.7, 2.0 Hz, 2H), 2.38 (s, 3H), 2.30 (s, 1.4H). **^{13}C {^{1}H} NMR** (100 MHz, CDCl$_3$) δ 174.7, 154.4, 142.6, 140.9, 139.9, 139.6, 137.5, 129.8, 129.3, 128.9, 128.2, 127.2, 127.1, 125.4, 123.3, 121.2, 117.8, 80.5, 51.7, 49.8, 39.7, 38.0, 36.1, 21.2. **HRMS(ESI-TOF)** m/z: [M+H$^+$] $^+$ $C_{32}H_{29}O_3$ calcd. 461.2117, found 461.2104.

Methyl-8-(m-tolyl)-2a-(p-tolyl)-2,2a,8,8a-tetrahydro-1H-cyclobuta[b]chromene-1-carboxylate (32)

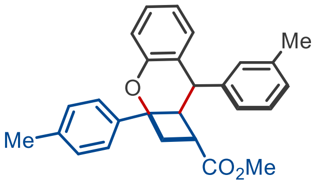

Following the general procedure(II), treatment of BCB **2j** (0.021 g, 0.1 mmol) and diol **1b** (0.036 g, 0.15 mmol) with $Yb(OTf)_3$ (0.003 g, 0.005 mmol) in THF:HFIP(7:3) (1.0 mL) at rt for 4 h, delivered compound **32**, which was purified by silica gel column chromatography (EtOAc: Hexane 5:95) to furnish the title compound **32** as semi solid in 67% (0.030g) yield (dr 2.7:1); ^{1}**H NMR** (400 MHz, DMSO-d_6) δ 7.37 (d, *J* = 8.1 Hz, 2H), 7.24 (d, *J* = 8.0 Hz, 1.2H), 7.21 – 7.16 (m, 4H), 7.10-7.06 (m, 3H), 7.04-7.00 (m, 1.7H), 6.94 (t, *J* = 7.3 Hz, 1.4H), 6.90 – 6.85 (m, 1.7H), 6.82 – 6.77 (m, 1H), 6.69 (s, 0.4H), 4.29 (d, *J* = 6.8 Hz, 1H), 4.13 (s, 0.4H), 3.64 (s, 1H), 3.51 (d, *J* = 9.2 Hz, 1.4H), 3.28 (s, 3H), 2.97 – 2.85 (m, 1.4H), 2.66 – 2.54 (m, 1.8H), 2.45 (d, *J* = 9.3 Hz, 1H), 2.28 (s, 3H), 2.24 (s, 4H), 2.04 (s, 1H). ^{13}**C {^{1}H} NMR** (100 MHz, DMSO-d_6) δ 173.7, 153.8, 140.3, 139.6, 137.5, 137.1, 129.4, 129.1, 128.8, 128.6, 128.4,128.3, 128.0, 127.4, 125.2, 123.8, 121.2, 117.5, 80.1, 51.4, 48.2, 37.2, 35.7, 21.1, 20.7. **HRMS(ESI-TOF)** m/z: [M+H$^+$] $^+$ $C_{27}H_{27}O_3$ calcd. 399.1960, found. 399.1923.

Methyl-6-methoxy-8-phenyl-2a-(p-tolyl)-2,2a,8,8a-tetrahydro-1H-cyclobuta[b]chromene-1-carboxylate (33)

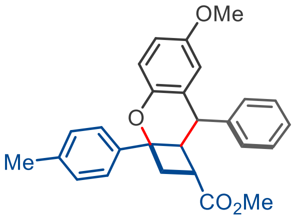

Following the general procedure(II), treatment of BCB **2j** (0.021 g, 0.1 mmol) and diol **1n** (0.034 g, 0.15 mmol) with $Yb(OTf)_3$ (0.003 g, 0.005 mmol) in THF:HFIP(7:3) (1.0 mL) at rt for 4 h, delivered compound **33**, which was purified by silica gel column chromatography (EtOAc: Hexane 5:95) to furnish the title compound **33** as semi solid in 70% (0.030g) yield (dr 10:1); ^{1}**H NMR** (400 MHz, DMSO-d_6) δ 7.36 (d, *J* = 8.1 Hz, 2H), 7.30 (t, *J* = 7.4 Hz, 2H), 7.24 (d, *J* = 7.2 Hz, 1H), 7.19 (d, *J* = 8.0 Hz, 2H), 7.12 (d,

J = 6.6 Hz, 2H), 6.95 (d, *J* = 8.8 Hz, 1H), 6.78 (dd, *J* = 8.8, 2.9 Hz, 1H), 6.33 (d, *J* = 2.6 Hz, 1H), 4.39 (d, *J* = 7.3 Hz, 1H), 3.56 (s, 3H), 3.55 – 3.52 (m, 1H), 3.26 (s, 3H), 2.88 (q, *J* = 8.9 Hz, 1H), 2.57 – 2.51 (m, 1H), 2.37 (dd, *J* = 11.7, 9.2 Hz, 1H), 2.28 (s, 3H). **^{13}C {^{1}H} NMR** (100 MHz, DMSO-d_6) δ 173.7, 153.6, 147.6, 140.4, 139.6, 137.1, 129.0, 128.9, 128.4, 126.7, 125.3, 125.2, 118.5, 114.1, 113.6, 80.0, 55.3, 51.4, 47.6, 37.0, 35.8, 20.7. **HRMS(ESI-TOF)** m/z: [M+H$^+$] $^+$ $C_{27}H_{27}O_4$ calcd. 415.1909, found. 415.1872.

Methyl-6-(tert-butyl)-8-phenyl-2a-(p-tolyl)-2,2a,8,8a-tetrahydro-1H-cyclobuta[b]chromene-1-carboxylate (34)

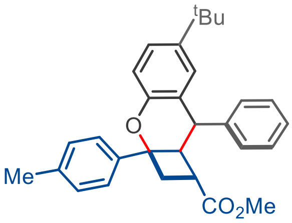

Following the general procedure(II), treatment of BCB **2j** (0.021 g, 0.1 mmol) and diol **1m** (0.038 g, 0.15 mmol) with Yb(OTf)$_3$ (0.003 g, 0.005 mmol) in THF:HFIP(7:3) (1.0 mL) at rt for 4 h, delivered compound **34**, which was purified by silica gel column chromatography (EtOAc: Hexane 5:95) to furnish the title compound **34** as semi solid in 62% (0.029g) yield (dr 3:1); **^{1}H NMR** (400 MHz, $CDCl_3$) δ 7.47 (d, *J* = 8.1 Hz, 2H), 7.34-7.30 (m, 2.2H), 7.28 – 7.24 (m, 2H), 7.22-7.17 (m, 4.2H), 7.15-7.12 (m, 1H), 7.08 – 7.01 (m, 2H), 7.00 (d, *J* = 1.5 Hz, 1H), 4.27 (d, *J* = 6.6 Hz, 1H), 4.22 (s, 0.2H), 3.75 (s, 0.5H), 3.67 (d, *J* = 7.7 Hz, 0.2H), 3.61 (dd, *J* = 9.4, 6.7 Hz, 1H), 3.35 (s, 3H), 3.11 (q, *J* = 8.9 Hz, 1H), 3.00 (dd, *J* = 18.2, 8.3 Hz, 0.2H), 2.81 (dd, *J* = 12.8, 8.4 Hz, 0.2H), 2.70 – 2.66 (m, 0.2H), 2.64 (s, 1H), 2.62 (s, 1H), 2.37 (s, 3H), 2.29 (s, 0.5H), 1.29 (s, 1.6H), 1.22 (s, 9H). **^{13}C {^{1}H} NMR** (100 MHz, $CDCl_3$) δ 174.7, 152.1, 152.0, 143.7, 140.5, 140.2, 137.4, 129.2, 128.4, 126.8, 126.6, 125.4, 125.0, 122.1, 117.0, 80.2, 51.6, 49.8, 40.2, 38.2, 36.0, 34.2, 31.6, 21.2. **HRMS(ESI-TOF)** m/z: [M+H$^+$] $^+$ $C_{30}H_{33}O_3$ calcd. 441.2430, found. 441.2411.

Methyl-8-(3,5-dimethylphenyl)-2a-(p-tolyl)-2,2a,8,8a-tetrahydro-1H-cyclobuta[b]chromene-1-carboxylate (35)

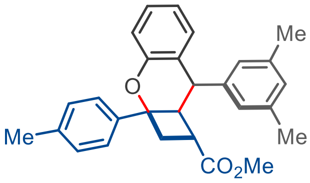

Following the general procedure(II), treatment of BCB **2j** (0.021 g, 0.1 mmol) and diol **1g** (0.034 g, 0.15 mmol) with Yb(OTf)3 (0.003 g, 0.005 mmol) in THF:HFIP(7:3) (1.0 mL) at rt for 4 h, delivered compound **35**, which was purified by silica gel column chromatography (EtOAc: Hexane 5:95) to furnish the title compound **35** as semi solid in 73% (0.033g) yield (dr 2.7:1); **^{1}H NMR** (400 MHz, DMSO-d_6) δ 7.37 (d, *J* = 8.0 Hz, 2H), 7.28 (d, *J* = 8.1 Hz, 1H), 7.23 – 7.16 (m, 3.5H), 7.14 – 7.05 (m, 2.2H), 7.01-6.99 (m, 1H), 6.93 (t, *J* = 7.4 Hz, 0.6H), 6.87-6.85 (m, 2H), 6.80 (d, *J* = 7.5 Hz, 1H), 6.71 (d, *J* = 13.9 Hz, 2H), 6.51 (s, 0.7H), 4.21 (d, *J* = 6.5 Hz, 1H), 4.05 (s, 0.4H), 3.73-3.69 (m, 1.4H), 3.32 (s, 3H), 2.97 – 2.84 (m, 1.5H), 2.68 – 2.52 (m, 2H), 2.48-2.42 (m, 1H), 2.27 (d, *J* = 13.5 Hz, 5H), 2.20 (s, 6.7H), 2.01 (s, 2.6H). **^{13}C {^{1}H} NMR** (100 MHz, DMSO-d_6) δ 173.8, 153.7, 140.1, 139.6, 137.4, 129.4, 129.1, 128.7, 128.2, 128.0, 125.8, 125.2, 124.0, 121.2, 117.5, 80.2, 51.4, 48.5, 44.4, 37.3, 35.8, 21.1, 20.7. **HRMS(ESI-TOF)** m/z: $[M+H^{+}]^{+}$ $C_{28}H_{29}O_3$ calcd. 413.2117, found. 413.2089.

Methyl-2a,8-bis(4-(tert-butyl)phenyl)-2,2a,8,8a-tetrahydro-1H-cyclobuta[b]chromene-1-carboxylate (36)

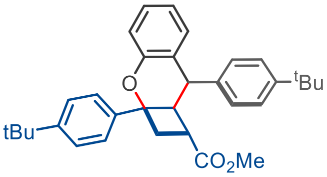

Following the general procedure(II), treatment of BCB **2e** (0.025 g, 0.1 mmol) and diol **1c** (0.038 g, 0.15 mmol) with Yb(OTf)3 (0.003 g, 0.005 mmol) in THF:HFIP(7:3) (1.0 mL) at rt for 4 h, delivered compound **36**, which was purified by silica gel column chromatography (EtOAc: Hexane 5:95) to furnish the title compound **36** as semi solid in 71% (0.037g) yield (dr 2:1); **^{1}H NMR** (400 MHz, $CDCl_3$) δ 7.59 (dd, *J* = 21.3, 8.3 Hz, 1H), 7.46 (d, *J* = 7.4 Hz, 2H), 7.41 (t, *J* = 6.8 Hz, 2H), 7.33 – 7.26 (m, 3H), 7.21 (dd, *J* = 8.6, 4.6 Hz, 2H), 7.15 – 7.07 (m, 5H), 6.97 (dd, *J* = 12.7, 7.9 Hz, 2H), 6.91 – 6.85

(m, 2H), 4.22 (d, J = 6.6 Hz, 1H), 4.19 (s, 1H), 3.76 – 3.68 (m, 1H), 3.66 – 3.56 (m, 2H), 3.32 (s, 3H), 3.12 – 3.06 (m, 1H), 3.01 – 2.78 (m, 2H), 2.65 – 2.60 (m, 2H), 1.35 (s, 5H), 1.32 (d, J = 2.1 Hz, 18H), 1.26 (s, 4H). **^{13}C {^{1}H} NMR** (100 MHz, $CDCl_3$) δ 174.8, 154.5, 150.6, 149.7, 140.0, 137.2, 129.9, 128.0, 127.9, 125.5, 125.3, 125.2, 123.6, 121.0, 117.7, 80.4, 51.6, 49.7, 39.5, 38.0, 36.1, 34.6(2), 31.5, 31.4. **HRMS(ESI-TOF)** m/z: $[M+Na^+]^+$ $C_{33}H_{38}NaO_3$ calcd. 505.2713, found. 505.2688.

methyl-2a-(4-(tert-butyl)phenyl)-8-(3-methoxyphenyl)-2,2a,8,8a-tetrahydro-1H-cyclobuta[b]chromene-1-carboxylate (37)

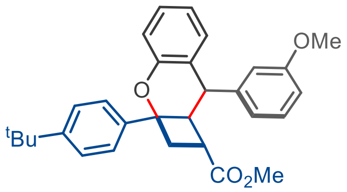

Following the general procedure(II), treatment of BCB **2e** (0.021g, 0.1 mmol) and diol **1n** (0.038 g, 0.15 mmol) with $Yb(OTf)_3$ (0.003 g, 0.005 mmol) in THF:HFIP(7:3) (1.0 mL) at rt for 4 h, delivered compound **37**, which was purified by silica gel column chromatography (EtOAc: Hexane 5:95) to furnish the title compound **37** as semi solid in 66% (0.029g) yield (dr 2.2:1); **^{1}H NMR** (400 MHz, $CDCl_3$) δ 7.48 (d, J = 8.6 Hz, 2H), 7.41 (d, J = 8.6 Hz, 2.4H), 7.28 (s, 2H), 7.21 (t, J = 7.9 Hz, 2.3H), 7.11-7.04 (m, 2.2H), 6.95 (t, J = 5.7 Hz, 1.4H), 6.90 – 6.84 (m, 1H), 6.81 – 6.74 (m, 2H), 6.70 (s, 0.7H), 6.66-6.63 (m, 1H), 6.37 (s, 0.4H), 4.20 (d, J = 6.4 Hz, 1H), 4.11 (s, 0.5H), 3.77 (s, 3H), 3.73 (s, 1.4H), 3.65 – 3.58 (m, 1.4H), 3.42 (s, 1.5H), 3.39 (s, 3H), 3.12 (q, J = 8.9 Hz, 1H), 3.01 (d, J = 9.5 Hz, 0.5H), 2.79 (dd, J = 12.3, 8.5 Hz, 0.6H), 2.68 – 2.59 (m, 2.4H), 1.32 (s, 9H), 1.27 (s, 4.2H). **^{13}C {^{1}H} NMR** (100 MHz, $CDCl_3$) δ 174.71 (s), 159.88 (s), 154.46 (s), 150.7, 142.0, 140.0, 129.8, 129.5, 128.5, 128.1, 125.5, 125.2, 121.2, 120.8, 117.7, 112.7, 80.5, 55.3, 51.7, 49.7, 40.3, 38.2, 36.2, 34.7, 31.5. **HRMS(ESI-TOF)** m/z: $[M+H^+]^+$ $C_{30}H_{33}O_4$ calcd. 457.2379, found. 457.2361.

Methyl-2a-(4-(tert-butyl)phenyl)-8-(naphthalen-2-yl)-2,2a,8,8a-tetrahydro-1H-cyclobuta[b]chromene-1-carboxylate (38)

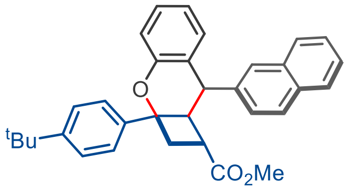

Following the general procedure(II), treatment of BCB **2e** (0.025 g, 0.1 mmol) and diol **1i** (0.038 g, 0.15 mmol) with Yb(OTf)3 (0.003 g, 0.005 mmol) in THF:HFIP(7:3) (1.0 mL) at rt for 4 h, delivered compound **38,** which was purified by silica gel column chromatography (EtOAc: Hexane 5:95) to furnish the title compound **38** as semi solid in 64% (0.032g) yield (dr 2:1); **^{1}H NMR** (400 MHz, $CDCl_3$) δ 7.97 (m, 0.8H), 7.82 – 7.70 (m, 4H), 7.66 – 7.58 (m, 2H), 7.52 – 7.40 (m, 7H), 7.38 – 7.34 (m, 1.4H), 7.32-7.29 (m, 1.7H), 7.24-7.20 (m, 2.5H), 7.17 – 7.09 (m, 3H), 6.96 (dd, J = 11.4, 4.2 Hz, 1.5H), 6.91 – 6.85 (m, 1H), 4.40 (d, J = 6.4 Hz, 1H), 4.30 (s, 0.5H), 3.75 (s, 1.4H), 3.70 – 3.63 (m, 1.6H), 3.19 (q, J = 8.9 Hz, 1H), 3.07 (s, 3H), 2.85 – 2.80 (m, 0.6H), 2.71 – 2.60 (m, 2.6H), 1.34 (d, J = 5.1 Hz, 9H), 1.24 (s, 4.7H). **^{13}C {^{1}H} NMR** (100 MHz, $CDCl_3$) δ 174.6, 154.6, 150.8, 139.9, 137.9, 133.7, 132.7, 129.8, 128.2, 128.1, 127.9, 127.7, 126.9, 126.1, 125.2, 125.1, 121.2, 117.8, 80.6, 51.4, 50.0, 45.6, 40.3, 36.2, 34.7, 31.5. **HRMS(ESI-TOF)** m/z: $[M+H^+]^+$ $C_{33}H_{33}O_3$ calcd. 477.2415, found. 477.2430.

Methyl-8-(thiophen-2-yl)-2a-(p-tolyl)-2,2a,8,8a-tetrahydro-1H-cyclobuta[b]chromene-1-carboxylate (39)

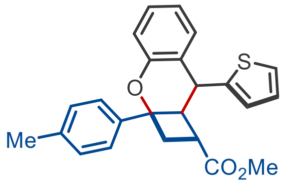

Following the general procedure(II), treatment of BCB **2j** (0.021 g, 0.1 mmol) and diol **1f** (0.030 g, 0.15 mmol) with Yb(OTf)3 (0.003 g, 0.005 mmol) in THF:HFIP(7:3) (1.0 mL) at rt for 4 h, delivered compound **39**, which was purified by silica gel column chromatography (EtOAc: Hexane 5:95) to furnish the title compound **39** as semi solid in 68% (0.030g) yield (dr 2:1); **^{1}H NMR** (400 MHz, DMSO-d_6) δ 7.45 – 7.37 (m, 3H),

7.30 – 7.23 (m, 2H), 7.22-7.19 (m, 2.5H), 7.11 (d, *J* = 8.0 Hz, 1.3H), 7.07 – 6.95 (m, 4.7H), 6.94 – 6.88 (m, 2H), 6.81-6.78 (m, 0.7H), 6.58 (d, *J* = 3.4 Hz, 0.6H), 4.74 (d, *J* = 6.4 Hz, 1H), 4.50 (d, *J* = 2.1 Hz, 0.6H), 3.64 (s, 2H), 3.62 – 3.58 (m, 1H), 3.43 (s, 3H), 2.90 (q, *J* = 8.9 Hz, 1H), 2.84 – 2.77 (m, 0.6H), 2.72-2.66 (m, 0.5H), 2.62 – 2.51 (m, 2.5H), 2.29 (s, 3H), 2.25 (s, 1.7H). **^{13}C {^{1}H} NMR** (100 MHz, DMSO-d_6) δ 173.7, 153.1, 146.4, 142.5, 139.6, 137.2, 126.8, 125.2, 125.1, 121.5, 117.7, 80.1, 51.7, 48.7, 37.1, 35.4, 35.2, 20.7. **HRMS(ESI-TOF)** m/z: [M+H$^+$] $^+$ $C_{24}H_{23}O_3S$ calcd. 391.1368, found. 391.1339.

Methyl-2a-(4-(tert-butyl)phenyl)-8-(thiophen-2-yl)-2,2a,8,8a-tetrahydro-1H-cyclobuta[b]chromene-1-carboxylate (40)

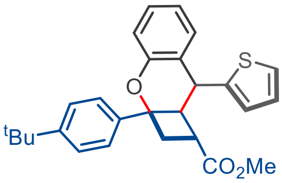

Following the general procedure(II), treatment of BCB **2e** (0.025 g, 0.1 mmol) and diol **1f** (0.030 g, 0.15 mmol) with $Yb(OTf)_3$ (0.003 g, 0.005 mmol) in THF:HFIP(7:3) (1.0 mL) at rt for 4 h, delivered compound **40**, which was purified by silica gel column chromatography (EtOAc: Hexane 5:95) to furnish the title compound **40** as semi solid in 57% (0.027g) yield (dr 2:1.1); **^{1}H NMR** (400 MHz, $CDCl_3$) δ 7.56 – 7.53 (m, 2H), 7.45 (d, *J* = 8.6 Hz, 2H), 7.30-7.26 (m, 5H), 7.24 (s, 2H), 7.09 (dd, *J* = 7.4, 2.3 Hz, 3H), 7.02-7.00 (m, 1H), 6.94 – 6.92 (m, 1H), 6.80-6.78 (m, 0.6H), 6.54 (d, *J* = 3.5 Hz, 0.5H), 4.56 (d, *J* = 5.8 Hz, 1H), 4.44 (d, *J* = 3.2 Hz, 0.6H), 3.77 – 3.74 (m, 2.4H), 3.56 (s, 2H), 3.12 (dd, *J* = 17.8, 8.9 Hz, 1H), 2.96 – 2.89 (m, 1.3H), 2.75 – 2.66 (m, 2.4H), 1.36 (s, 9H), 1.31 (s, 5H). **^{13}C {^{1}H} NMR** (100 MHz, $CDCl_3$) δ 174.8, 153.9, 150.8, 142.7, 140.1, 129.1, 128.4, 126.9, 125.5, 125.2, 125.1,124.4, 121.4, 117.9, 80.5, 51.9, 50.5, 38.2, 36.1, 35.7, 34.7, 31.5. **HRMS(ESI-TOF)** m/z: [M+H$^+$] $^+$ $C_{27}H_{29}O_3S$ calcd. 433.1837, found, 433.1824.

9. Product Modification :

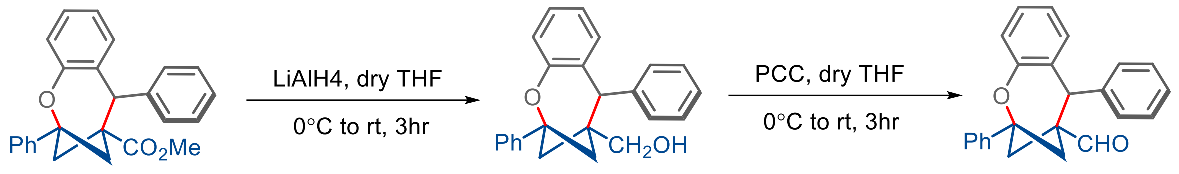

3 **41**, 65% **42**, 70%

9.1 $LiAlH_4$ reduction of 3

To a 25 mL two neck round bottom flask equipped with stir bar, $LiAlH_4$ (0.014 g, 0.35 mmol) was dissolved in dry THF (1.0 mL). The reaction mixture was cooled to 0 °C with an ice bath. Then, the solution of **3** (0.037 g, 0.10 mmol) in dry THF (1.0 mL) was added slowly and reaction mixture was allowed to stir for 3 h at room temperature. After this time, the reaction was cooled to 0 °C and quenched with 10% KOH solution (2.0 mL). The resulting suspension was filtered through celite, washed with ethyl acetate. The combined filtrate was dried, concentrated and purified by flash column chromatography (Pet. ether /EtOAc = 80/20) of the crude reaction mixture afforded compound **41** as a semi solid (0.023 g, 65% yield). **^{1}H NMR** (400 MHz, $CDCl_3$) δ 7.56 (d, *J* = 7.4 Hz, 2H), 7.42 (t, *J* = 7.5 Hz, 2H), 7.37 – 7.27 (m, 3H), 7.24-7.22(m, 1H), 7.18-7.14 (m, 3H), 7.09 (d, *J* = 7.6 Hz, 1H), 7.02 (d, *J* = 7.6 Hz, 1H), 6.83 (t, *J* = 6.9 Hz, 1H), 4.65 (s, 1H), 3.57 (d, *J* = 10.9 Hz, 1H), 3.47 (d, *J* = 10.9 Hz, 1H), 2.74 (dd, *J* = 12.8, 5.9 Hz, 1H), 2.67 (dd, *J* = 12.6, 5.8 Hz, 1H), 2.59 (d, *J* = 12.6 Hz, 1H), 2.32 (d, *J* = 13.0 Hz, 1H). **^{13}C {^{1}H} NMR** (101 MHz, $CDCl_3$) δ 153.8, 144.7, 143.1, 134.4, 129.9, 128.5, 128.3, 127.8, 127.6, 127.5, 126.6, 125.0, 121.3, 121.1, 80.6, 68.4, 53.1, 40.6, 38.6, 37.3. **HRMS(ESI-TOF)** m/z: $[M+H^+]^+$ $C_{24}H_{23}O_2$ calcd. 343.1698, found. 343.1674.

9.2 Oxidation of 41

To a 25 mL two neck round bottom flask equipped with stir bar, 41 (0.034 g, 0.10 mmol) was dissolved in dry THF (1.0 mL). Then, PCC (0.032 g, 1.5 equiv) was added slowly, the reaction mixture was cooled to 0 °C with an ice bath, then reaction mixture was allowed to stir for 2 h at rt. After this time, the reaction solvent was concentrated

and purified by flash column chromatography (Pet. ether /EtOAc = 95/10) of the crude reaction mixture afforded 9-methyl-1,4-diphenyl-1,2,4,9-tetrahydro-3H-1,3-methanocarbazole-3- carbaldehyde **42** as a white semi solid (0.024 g, 70% yield). **^{1}H NMR** (400 MHz, $CDCl_3$) δ 9.80 (s, 1H), 7.54 (d, *J* = 7.3 Hz, 2H), 7.44 (t, *J* = 7.5 Hz, 2H), 7.37 (d, *J* = 7.3 Hz, 1H), 7.30-7.20 (M, 4H), 7.14-7.09 (M, 4H), 6.95 – 6.90 (m, 1H), 5.07 (s, 1H), 3.01 (d, *J* = 12.7 Hz, 1H), 2.79 – 2.70 (m, 2H), 2.65 (d, *J* = 12.5 Hz, 1H). **^{13}C {^{1}H} NMR** (100 MHz, $CDCl_3$) δ 201.7, 153.7 143.6, 141.9, 133.7, 129.4, 128.7, 128.6,128.5, 128.0, 127.2, 126.1, 125.3, 121.8, 121.6, 80.7, 51.6, 49.9, 38.0, 35.5. **HRMS(ESI-TOF)** m/z: $[M+H^+]^+$ $C_{24}H_{21}O_2$ calcd. 341.1542, found. 341.1539.

9.3 $LiAlH_4$ reduction of 26

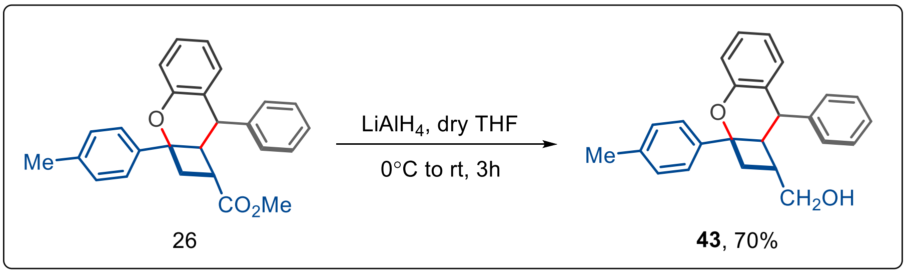

Compound **43** was prepared using the known procedure.[10] To a 25 mL two neck round bottom flask equipped with stir bar, LiAlH4 (0.014 g, 0.35 mmol) was dissolved in dry THF (1.0 mL). The reaction mixture was cooled to 0 °C with an ice bath. Then, the solution of 3 (0.041 g, 0.10 mmol) in dry THF (1.0 mL) was added slowly and reaction mixture was allowed to stir for 3 h at room temperature. After this time, the reaction was cooled to 0 °C and quenched with 10% KOH solution (2.0 mL). The resulting suspension was filtered through celite, washed with ethyl acetate. The combined filtrate was dried, concentrated and purified by flash column chromatography (Pet. ether /EtOAc = 80/20) of the crude reaction mixture afforded **43** as a semi solid (0.030 g, 70% yield) (dr 3:1). **^{1}H NMR** (400 MHz, DMSO-d_6) δ 7.34 (d, *J* = 8.1 Hz, 2H), 7.22 – 7.14 (m, 4H), 7.13 – 7.04 (m, 3H), 7.03 – 6.95 (m, 2H), 6.89 (t, *J* = 6.4 Hz, 3H), 6.84 – 6.80 (m, 1.4H), 6.69 (d, *J* = 8.7 Hz, 0.6H), 4.17 (d, *J* = 6.9 Hz, 1H), 4.03 (d, *J* = 1.9 Hz, 0.3H), 3.74 (s, 3H), 3.65 (s, 1.2H), 3.11 (d, *J* = 5.3 Hz, 0.8H), 3.07 – 3.00 (m, 1H), 2.94 – 2.87 (m, 1H), 2.74-2.70 (M, 1H), 2.27 (s, 2H), 2.24 (s, 0.7H). **^{13}C {^{1}H} NMR** (101 MHz, DMSO-d_6) δ 158.0, 154.1, 140.9, 136.5, 133.4, 129.4, 128.9, 128.6, 125.4, 125.2, 120.7, 117.3, 113.8, 80.4, 62.2, 55.1, 45.3, 36.2, 35.0, 20.7. **HRMS(ESI-TOF)** m/z: $[M+Na^+]^+$ $C_{26}H_{26}NaO_3$ calcd. 409.1750, found. 409.1780.

10. 1H and 13C NMR Spectra of Products (3-43):

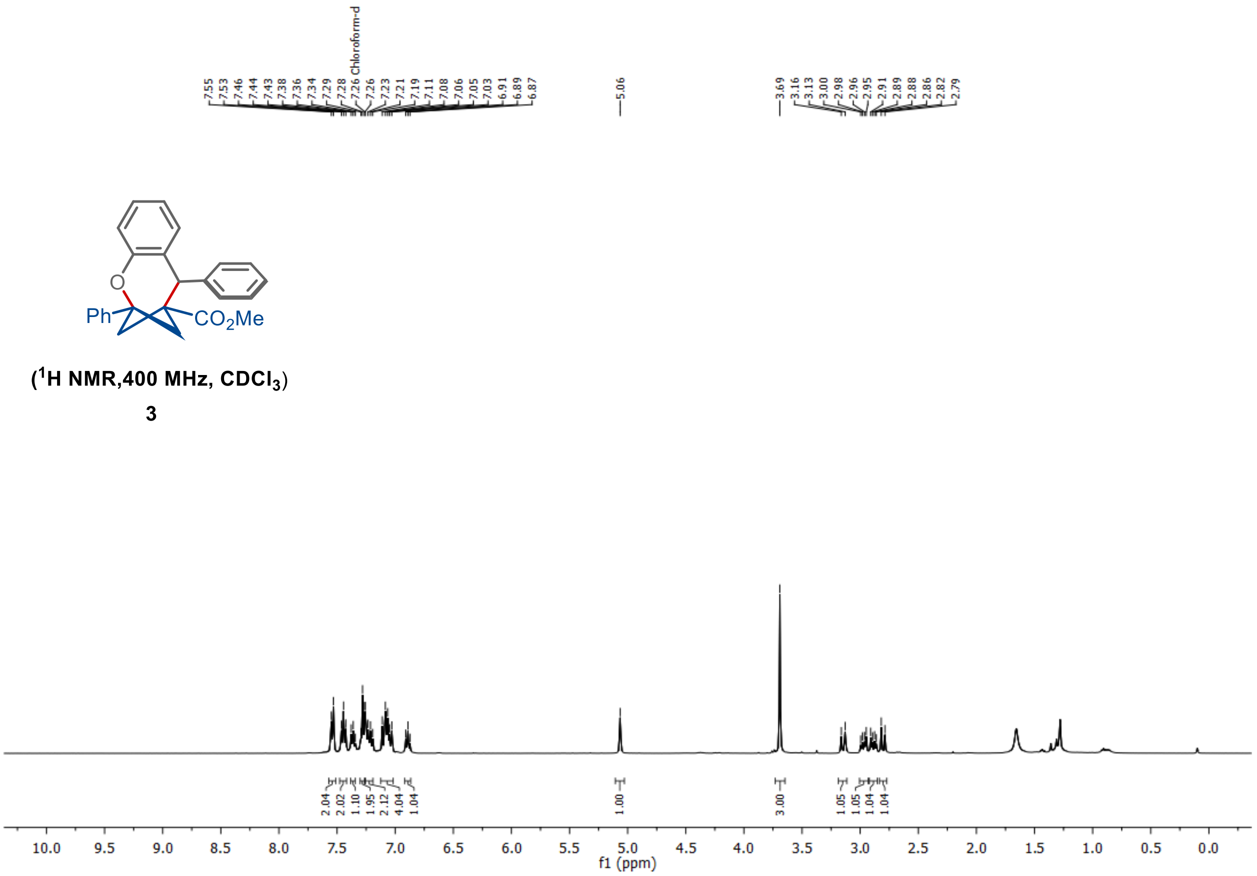

(^{1}H NMR,400 MHz, $CDCl_3$)

3

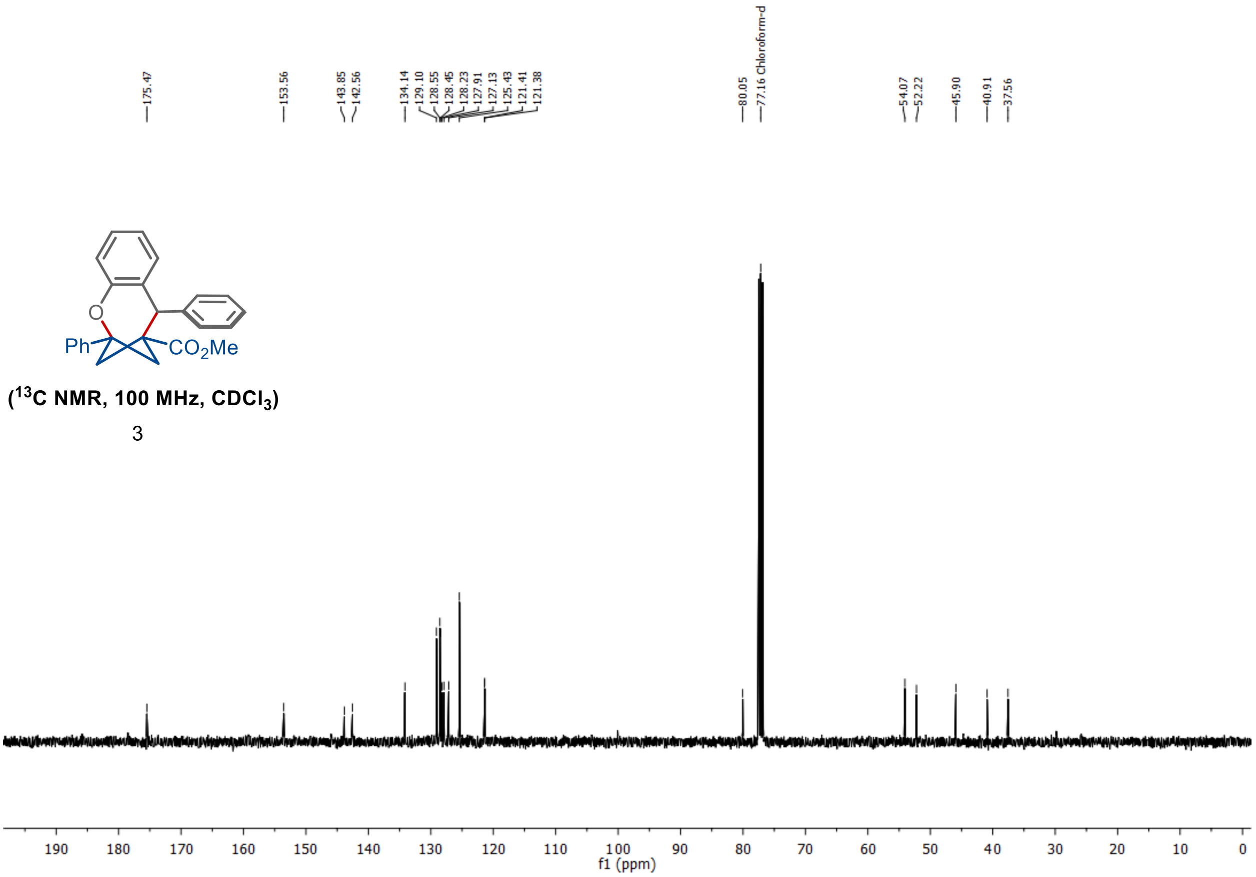

(^{13}C NMR, 100 MHz, $CDCl_3$)

3

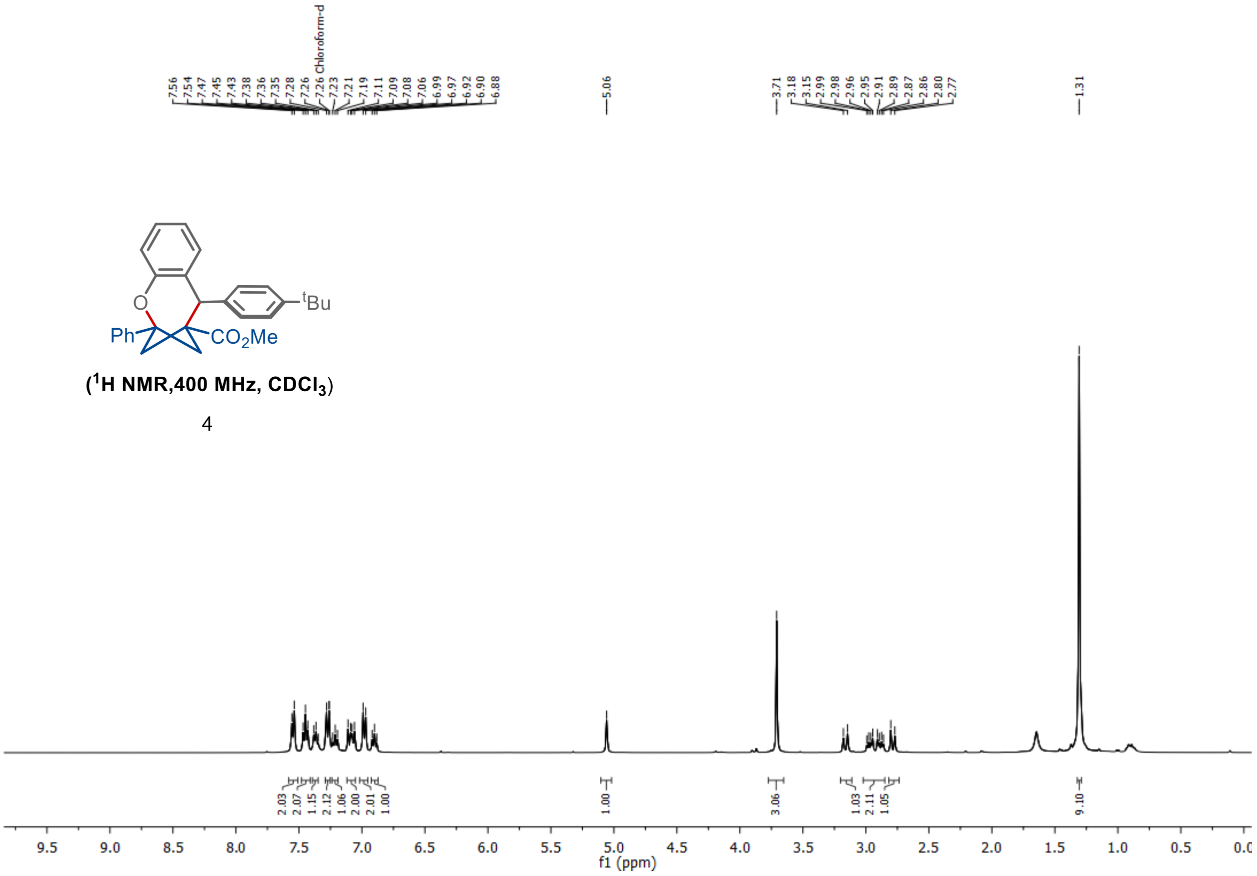

7.56
7.54
7.47
7.45
7.43
7.38
7.36
7.35
7.28
7.26
7.26 Chloroform-d
7.23
7.21
7.19
7.11
7.09
7.08
7.06
6.99
6.97
6.92
6.90
6.88
5.06
3.71
3.18
3.15
2.99
2.98
2.96
2.95
2.91
2.89
2.87
2.86
2.80
2.77
1.31
O
tBu
Ph
CO_2Me
(^{1}H NMR,400 MHz, $CDCl_3$)
4
2.03
2.07
1.15
2.12
1.06
2.00
2.01
1.00
1.00
3.06
1.03
2.11
1.05
9.10
9.5
9.0
8.5
8.0
7.5
7.0
6.5
6.0
5.5
5.0
4.5
4.0
3.5
3.0
2.5
2.0
1.5
1.0
0.5
0.0
f1 (ppm)

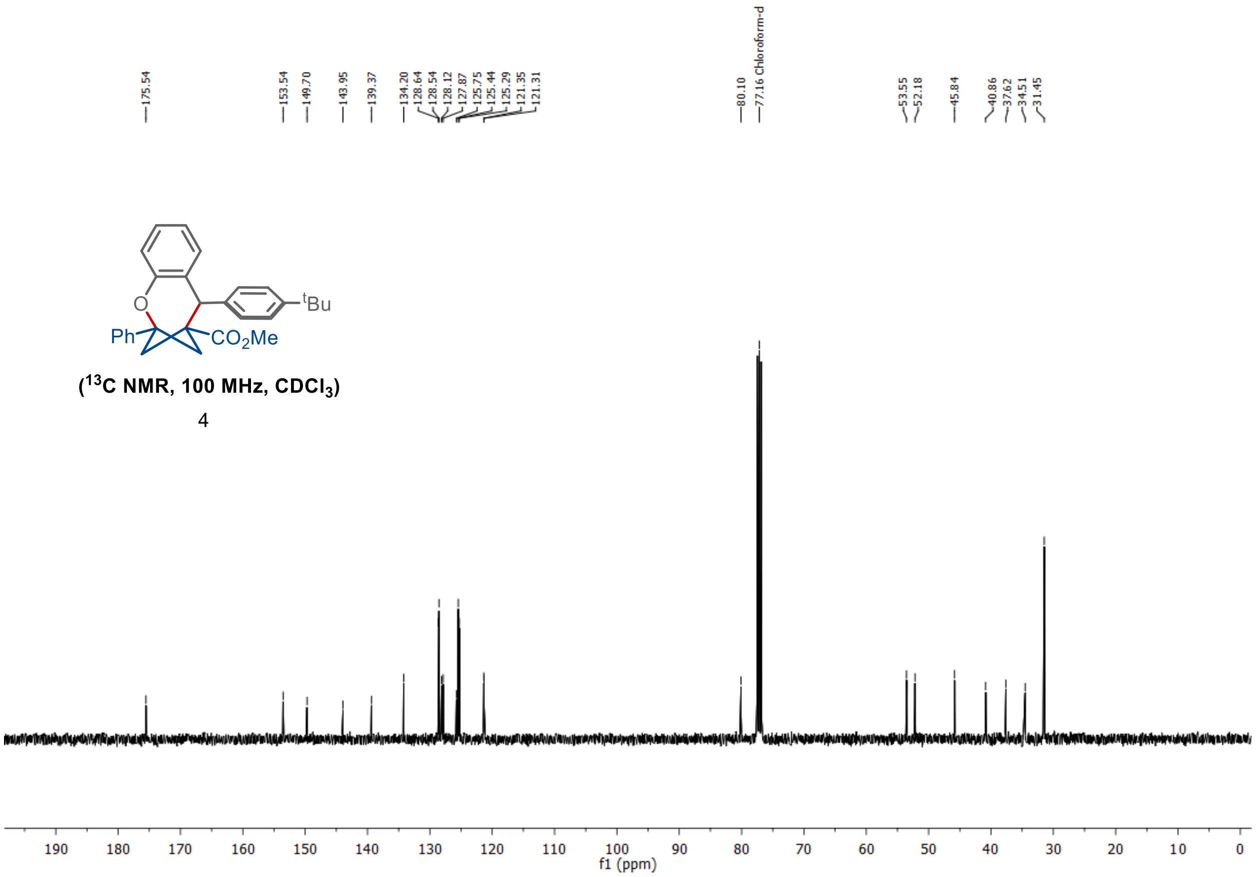

175.54
153.54
149.70
143.95
139.37
134.20
128.64
128.54
128.12
127.87
125.75
125.44
125.29
121.35
121.31
80.10
77.16 Chloroform-d
53.55
52.18
45.84
40.86
37.62
34.51
31.45
O
tBu
Ph
CO_2Me
(^{13}C NMR, 100 MHz, $CDCl_3$)
4
190
180
170
160
150
140
130
120
110
100
90
80
70
60
50
40
30
20
10
0
f1 (ppm)

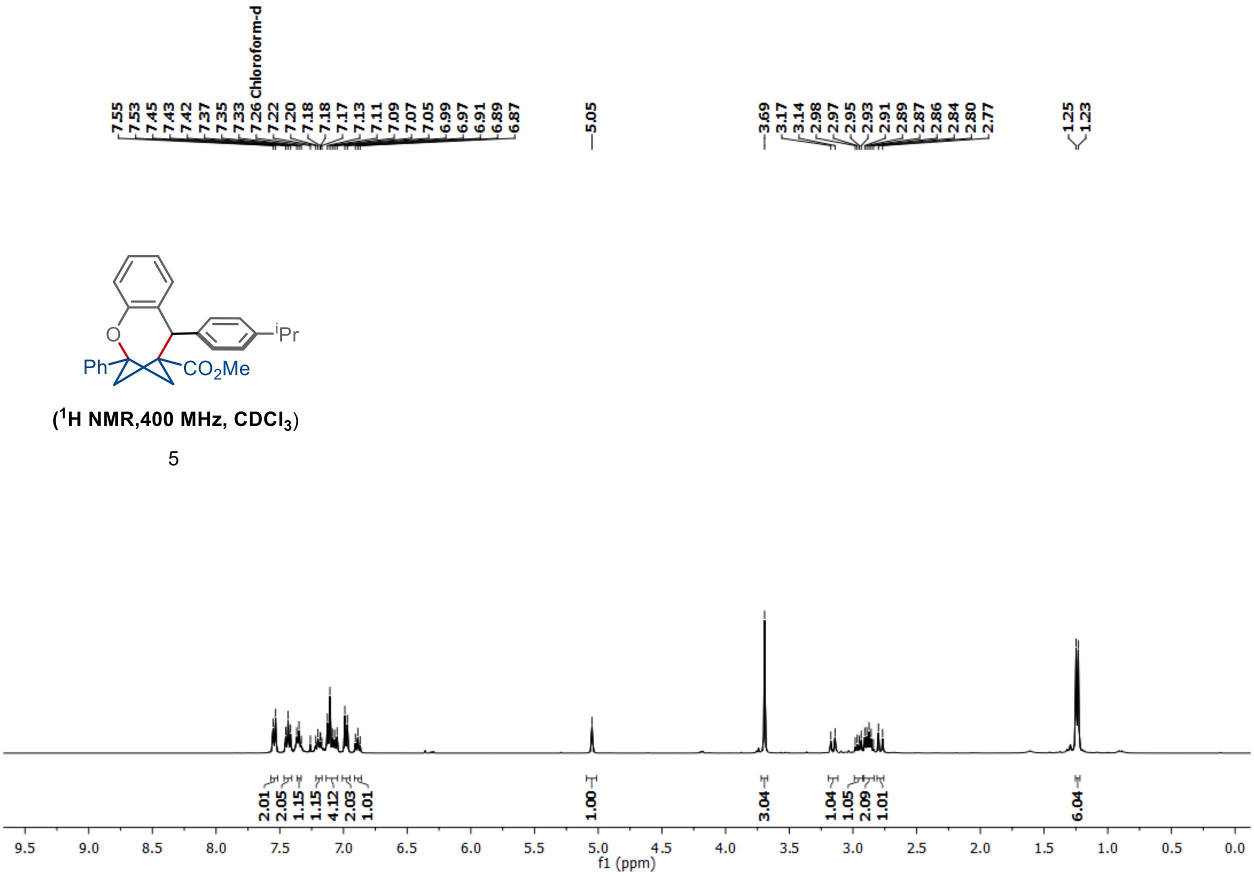

(^{1}H NMR,400 MHz, CDCl$_3$)

5

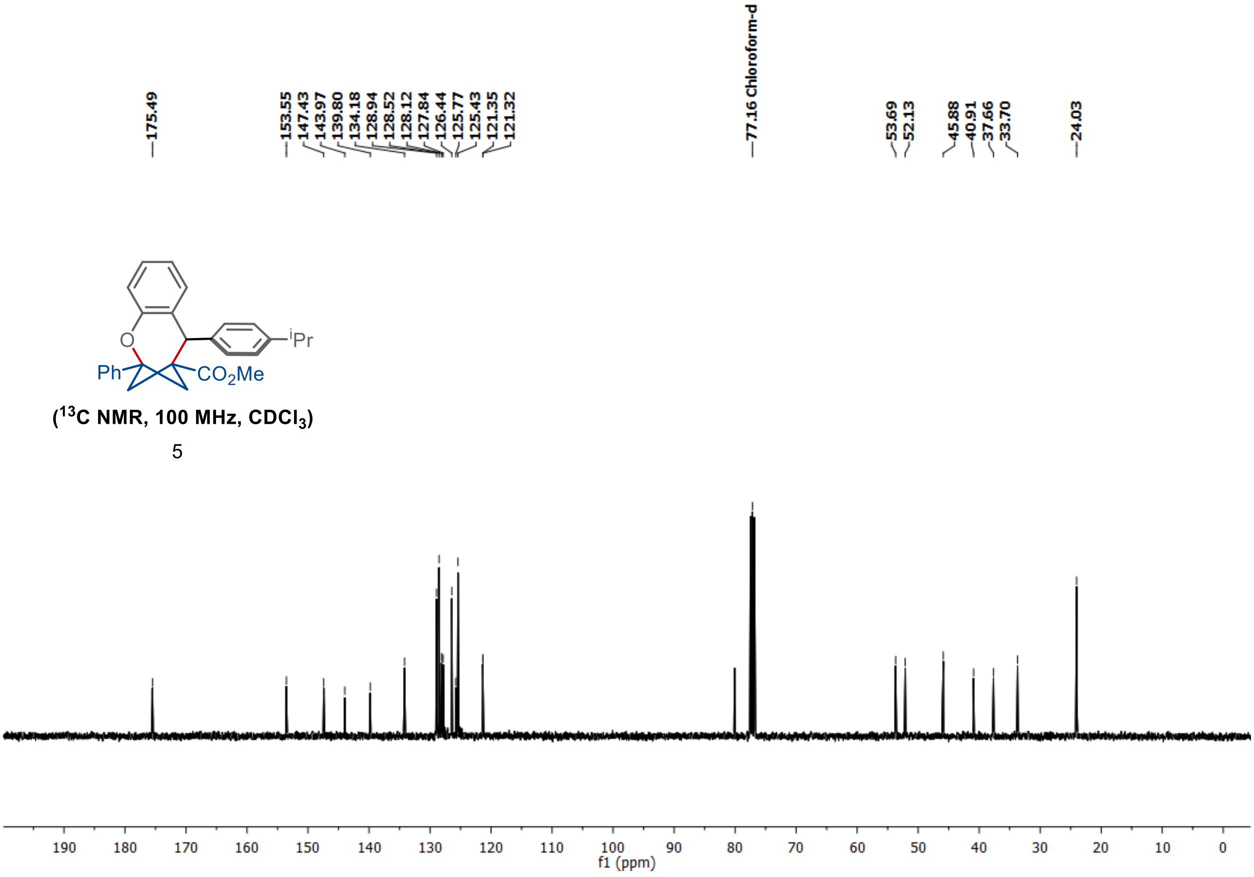

(^{13}C NMR, 100 MHz, CDCl$_3$)

5

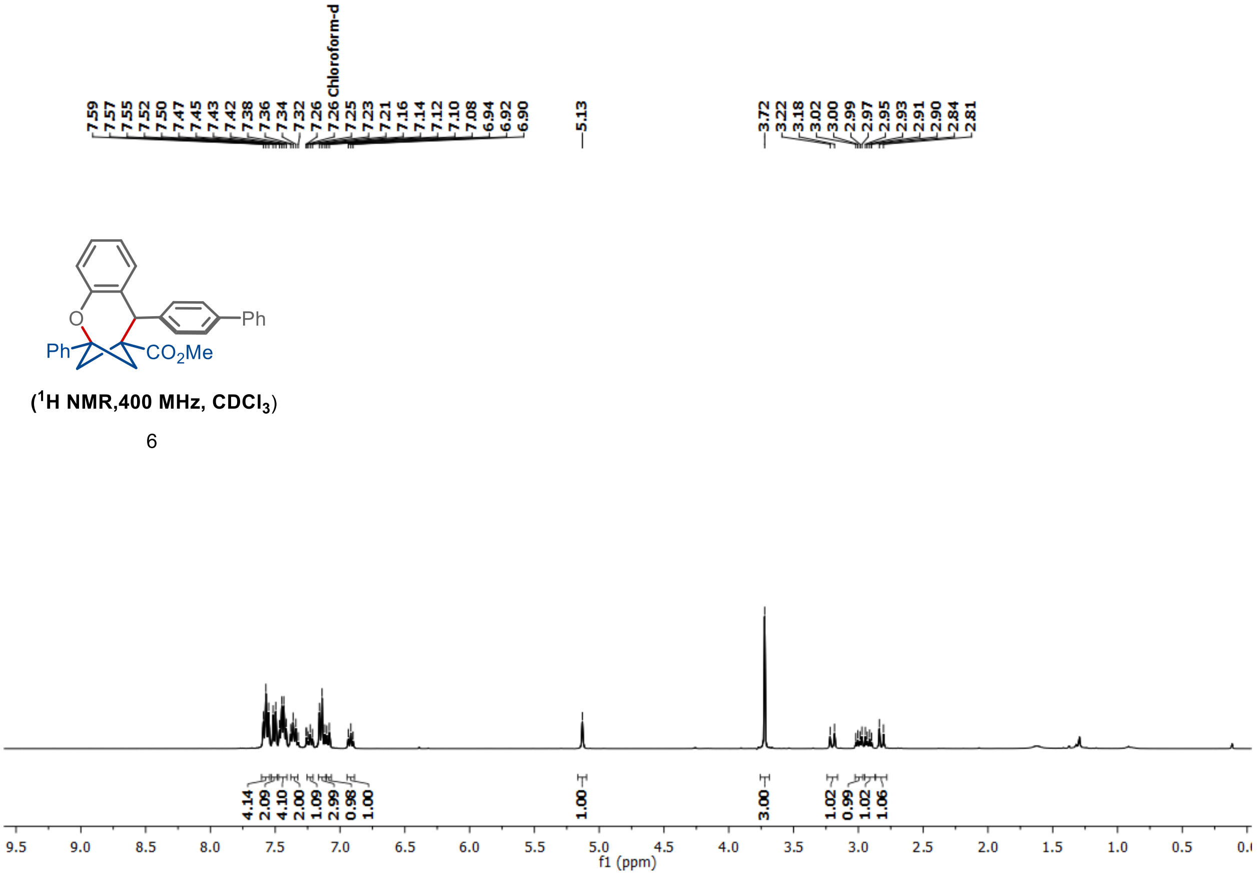
7.59
7.57
7.55
7.52
7.50
7.47
7.45
7.43
7.42
7.38
7.36
7.34
7.32
7.26
7.26 Chloroform-d
7.25
7.23
7.21
7.16
7.14
7.12
7.10
7.08
6.94
6.92
6.90
5.13
3.72
3.22
3.18
3.02
3.00
2.99
2.97
2.95
2.93
2.91
2.90
2.84
2.81
O
Ph
Ph
CO2Me
(1H NMR,400 MHz, CDCl3)
6
4.14
2.09
4.10
2.00
1.09
2.99
0.98
1.00
1.00
3.00
1.02
0.99
1.02
1.06
f1 (ppm)

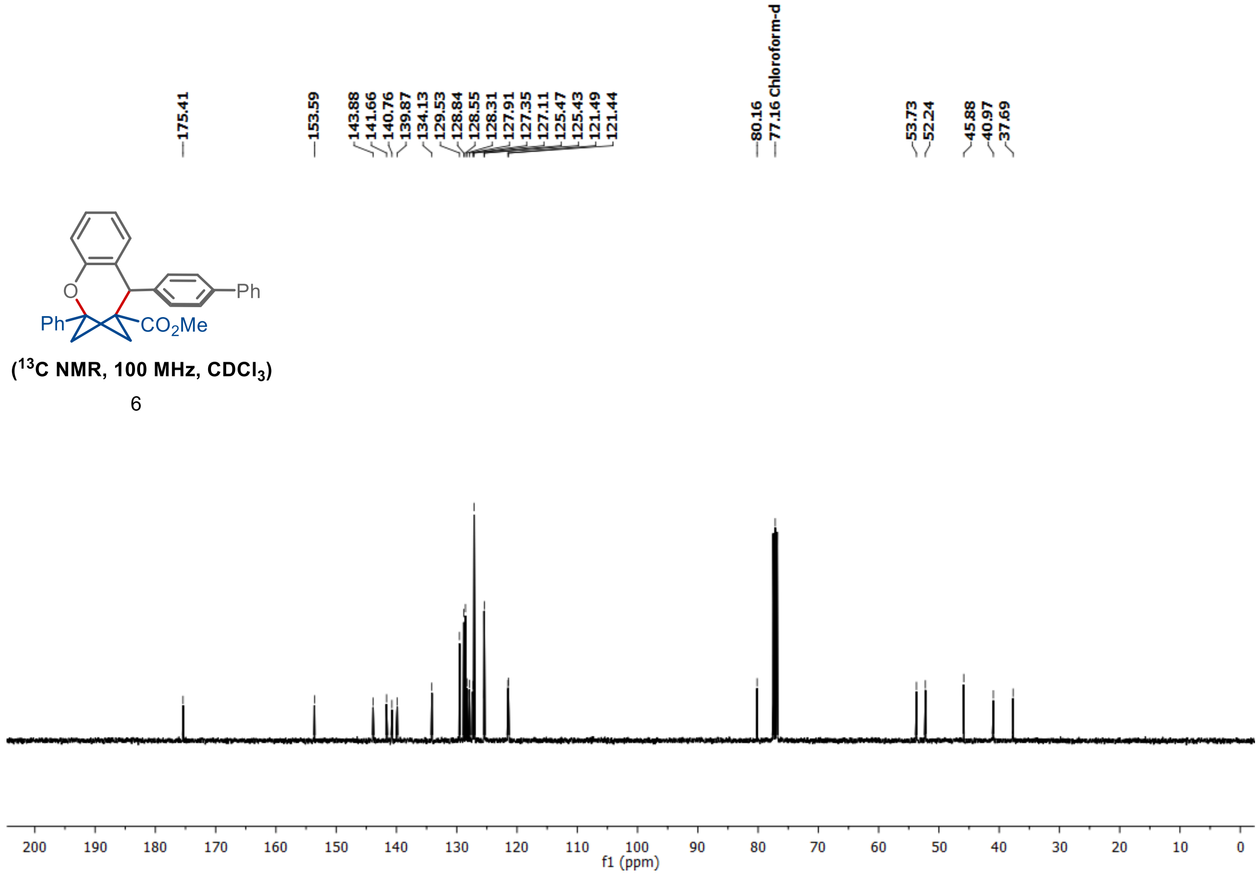
175.41
153.59
143.88
141.66
140.76
139.87
134.13
129.53
128.84
128.55
128.31
127.91
127.35
127.11
125.47
125.43
121.49
121.44
80.16
77.16 Chloroform-d
53.73
52.24
45.88
40.97
37.69
O
Ph
Ph
CO2Me
(13C NMR, 100 MHz, CDCl3)
6
f1 (ppm)

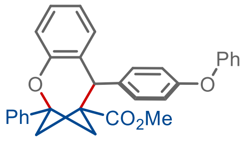

(^{1}H NMR,400 MHz, CDCl$_3$)

7

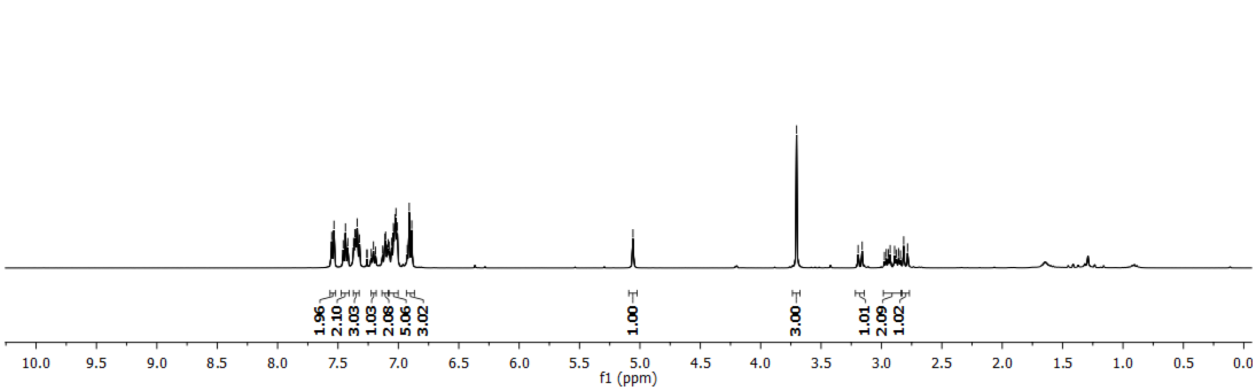

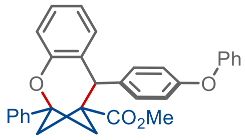

(^{13}C NMR, 100 MHz, CDCl$_3$)

7

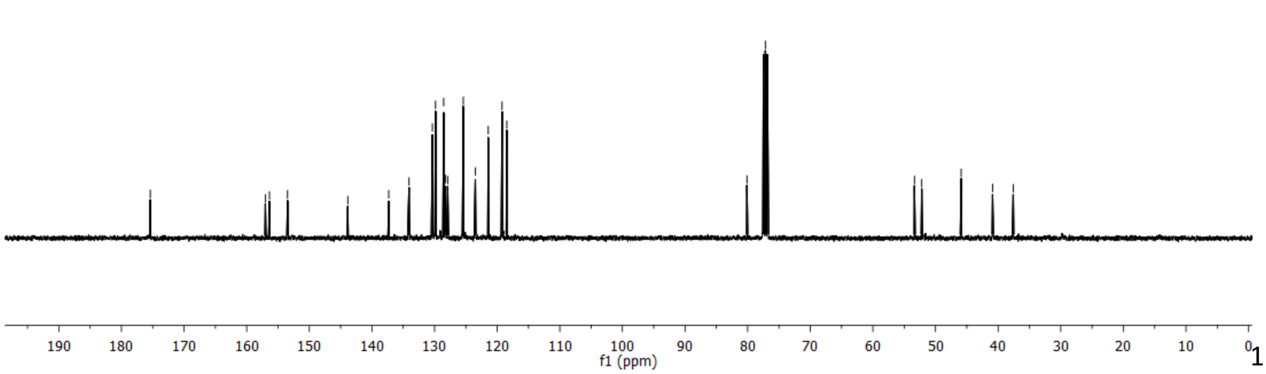

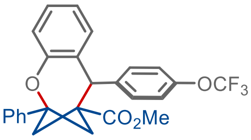

(¹H NMR,400 MHz, CDCl$_3$)

8

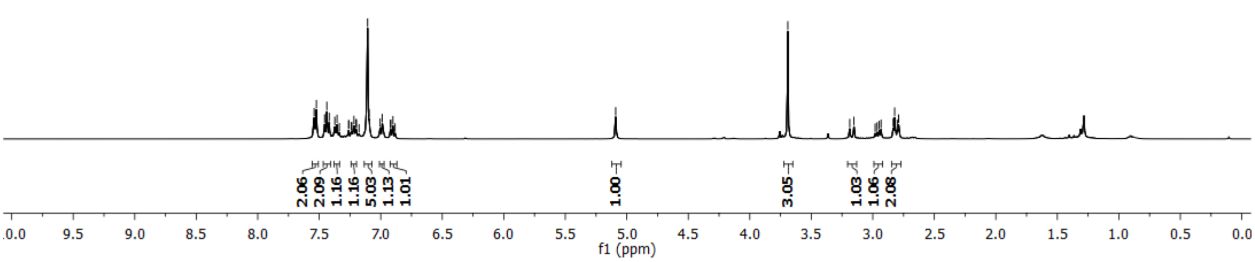

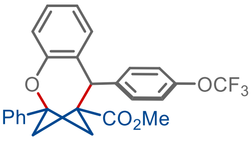

(¹³C NMR, 100 MHz, CDCl$_3$)

8

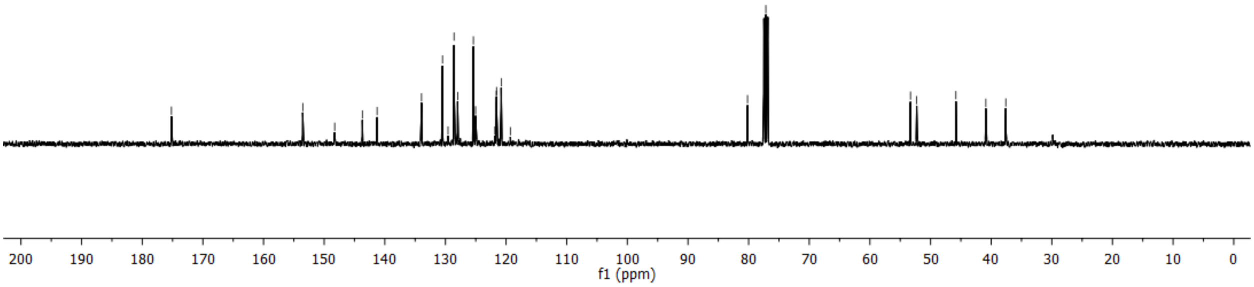

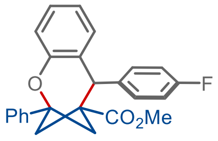

(^{1}H NMR,400 MHz, $CDCl_3$)

9

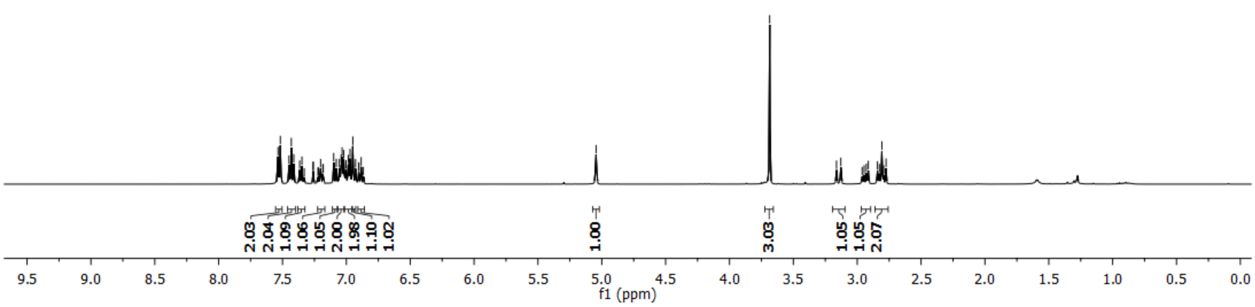

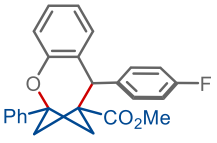

(^{13}C NMR, 100 MHz, $CDCl_3$)

9

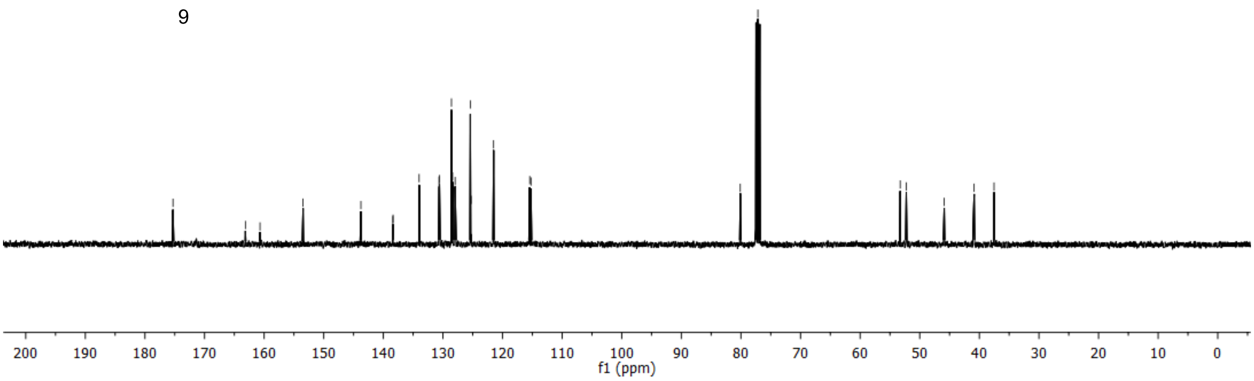

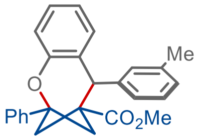

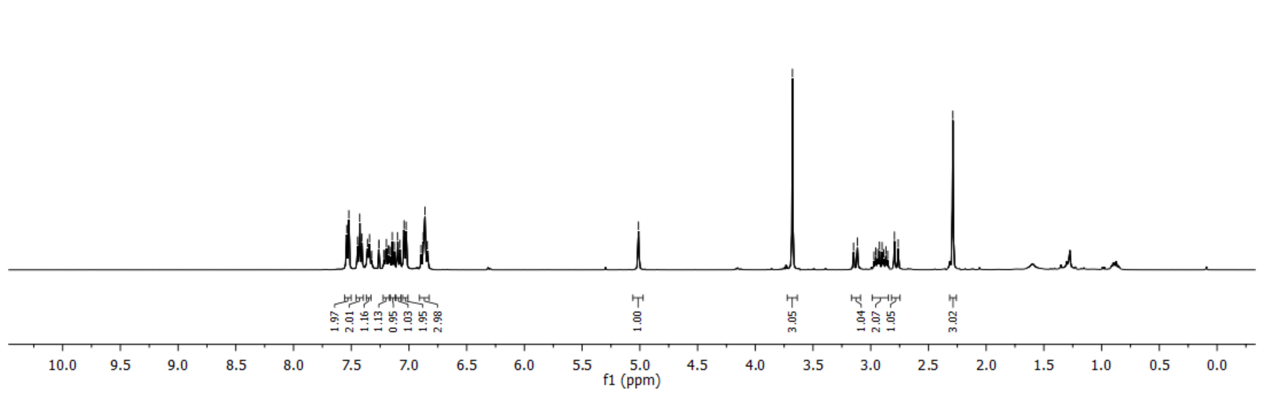

(^{1}H NMR,400 MHz, $CDCl_3$)

10

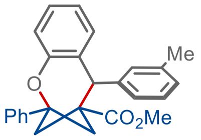

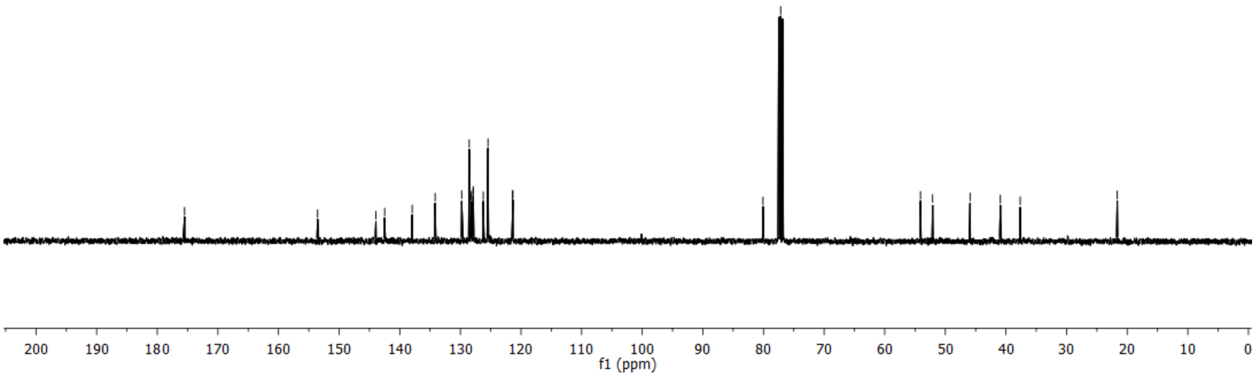

(^{13}C NMR, 100 MHz, $CDCl_3$)

10

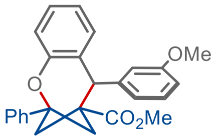

(^{13}C NMR, 100 MHz, $CDCl_3$)

11

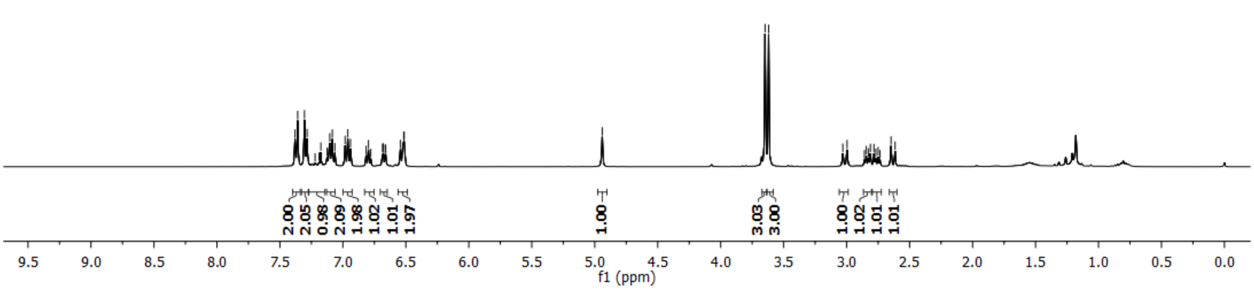

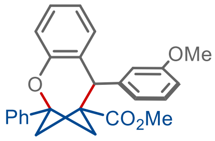

(^{1}H NMR,400 MHz, $CDCl_3$)

11

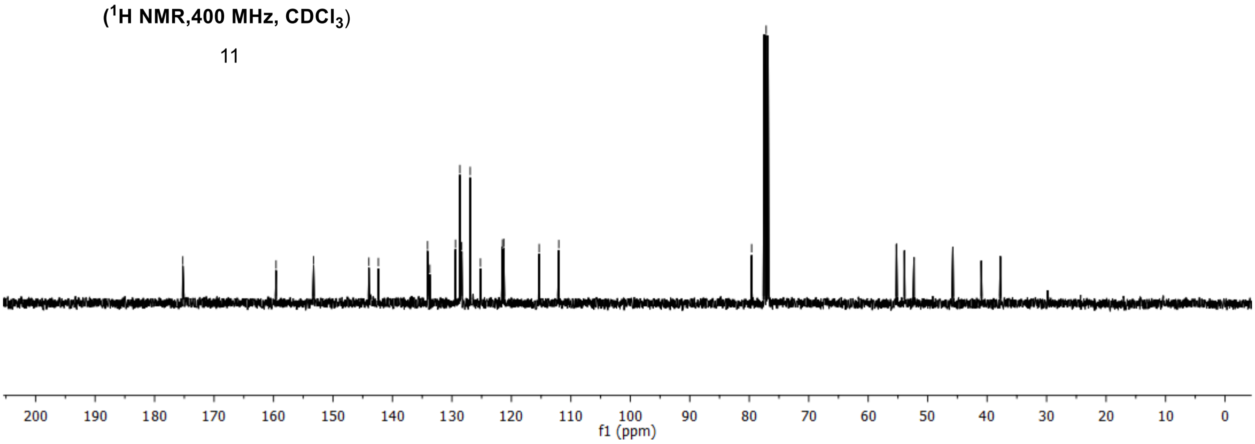

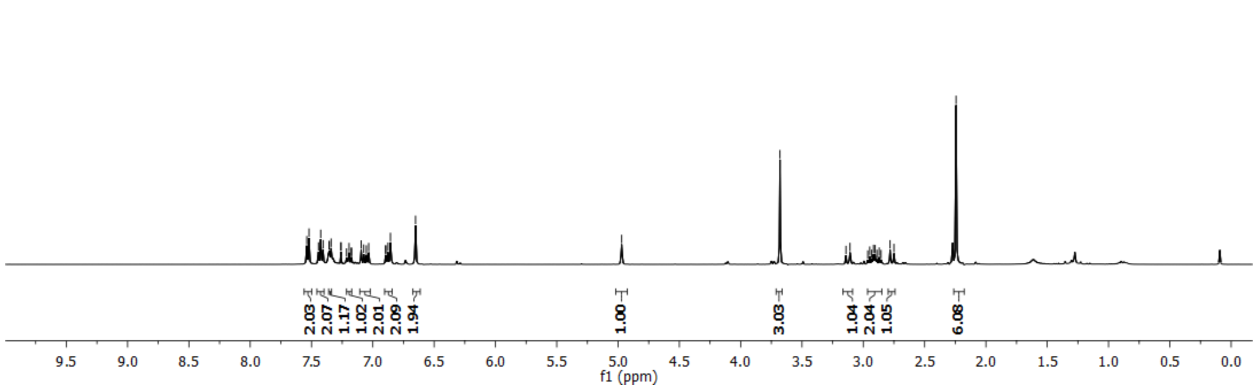

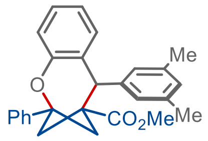

(^{1}H NMR,400 MHz, $CDCl_3$)

12

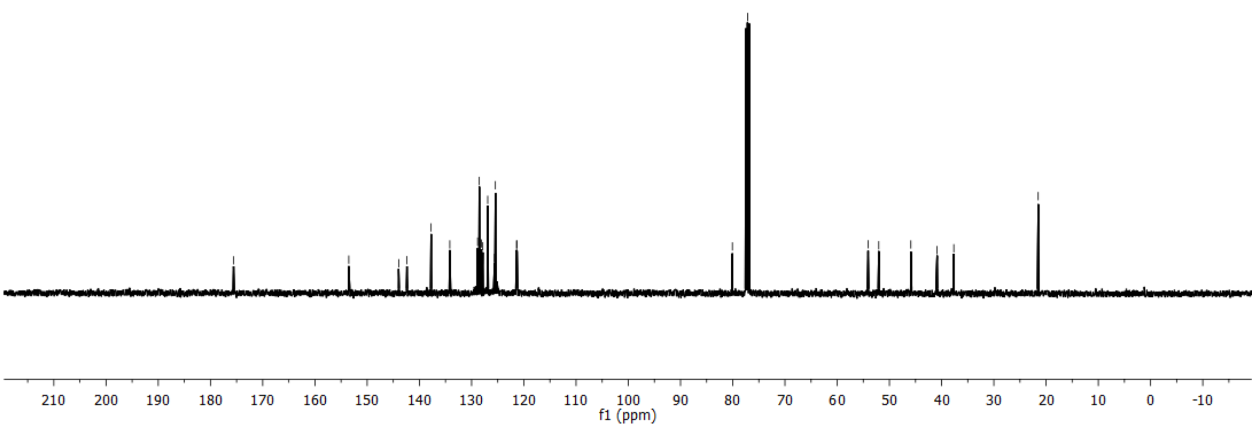

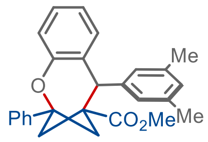

(^{13}C NMR, 100 MHz, $CDCl_3$)

12

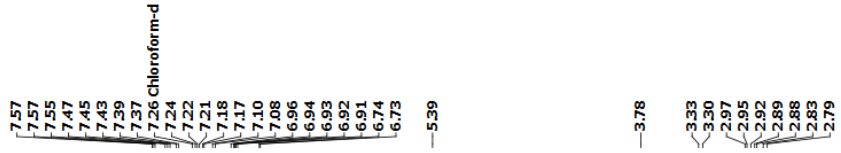

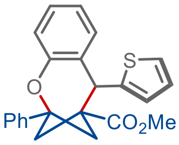

(^{1}H NMR,400 MHz, $CDCl_3$)

13

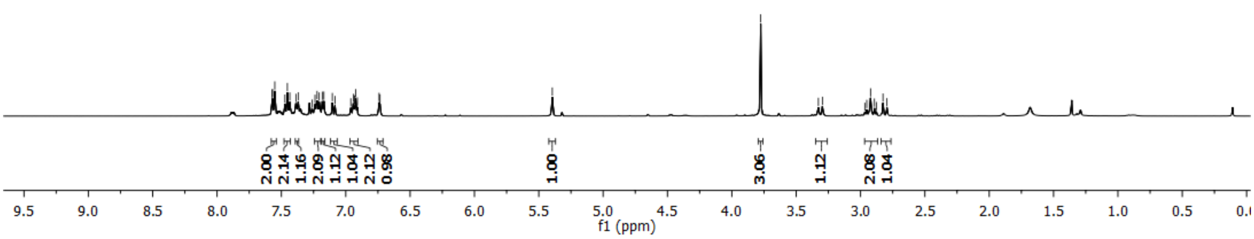

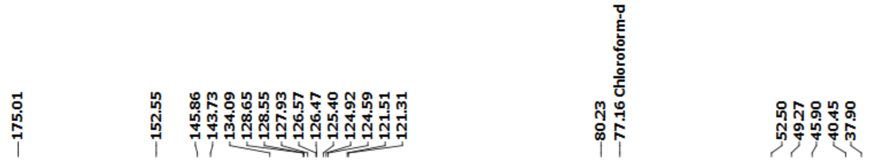

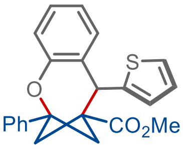

(^{13}C NMR, 100 MHz, $CDCl_3$)

13

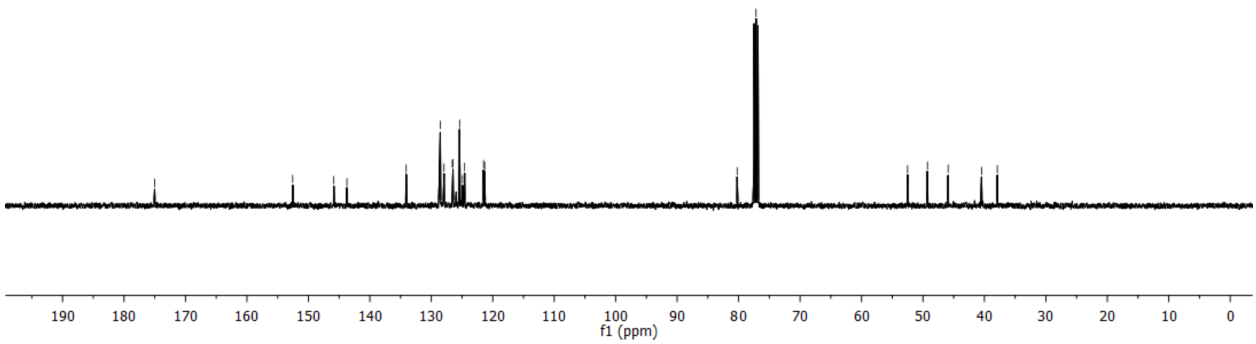

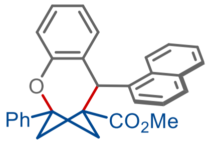

(^{1}H NMR,400 MHz, CDCl$_3$)

14

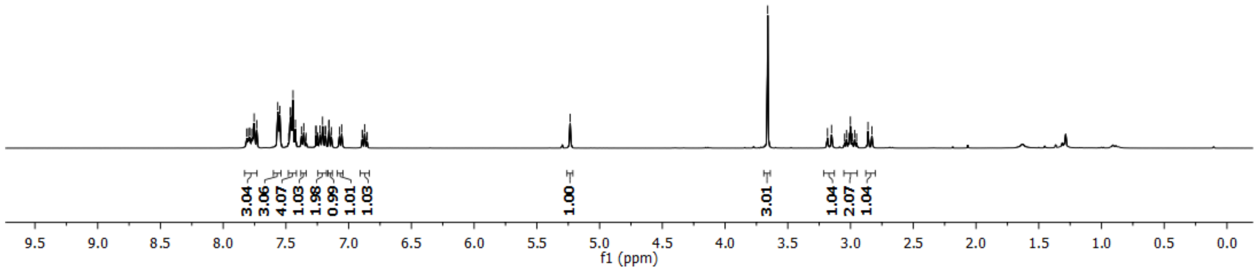

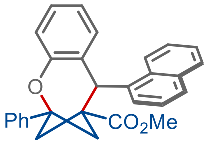

(^{13}C NMR, 100 MHz, CDCl$_3$)

14

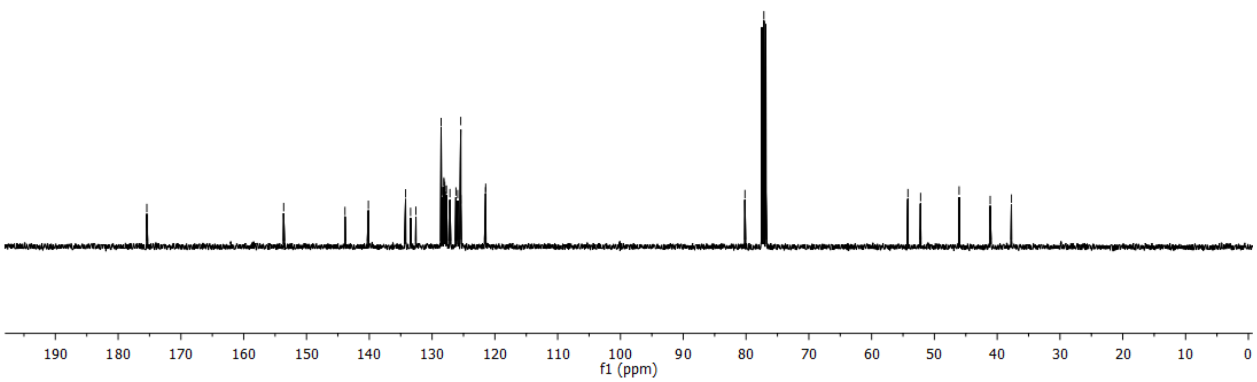

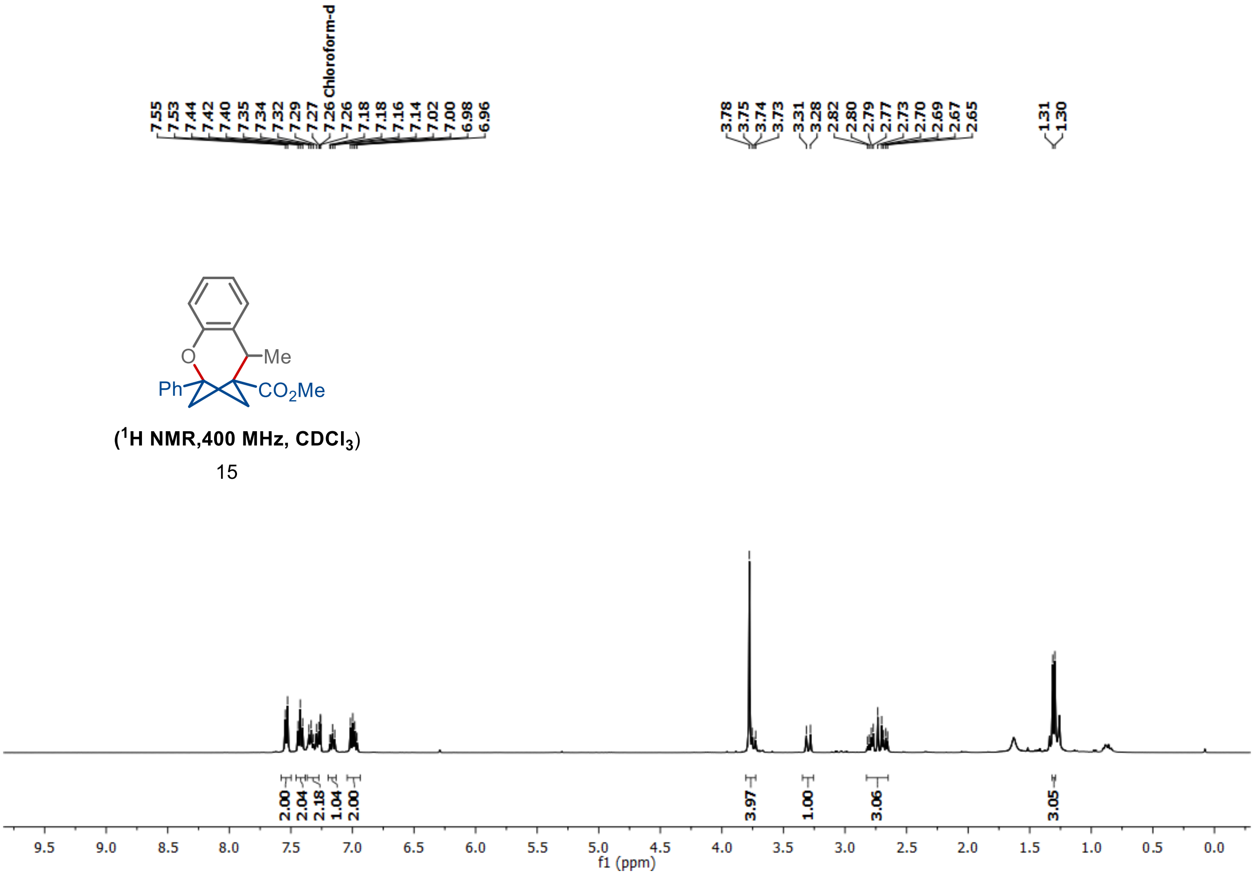
7.55 7.53 7.44 7.42 7.40 7.35 7.34 7.32 7.29 7.27 7.26 Chloroform-d 7.26 7.18 7.18 7.16 7.14 7.02 7.00 6.98 6.96
3.78 3.75 3.74 3.73 3.31 3.28 2.82 2.80 2.79 2.77 2.73 2.70 2.69 2.67 2.65
1.31 1.30
O
Me
Ph
CO2Me
(1H NMR,400 MHz, CDCl3)
15
2.00 2.04 2.18 1.04 2.00 3.97 1.00 3.06 3.05
9.5 9.0 8.5 8.0 7.5 7.0 6.5 6.0 5.5 5.0 4.5 4.0 3.5 3.0 2.5 2.0 1.5 1.0 0.5 0.0
f1 (ppm)

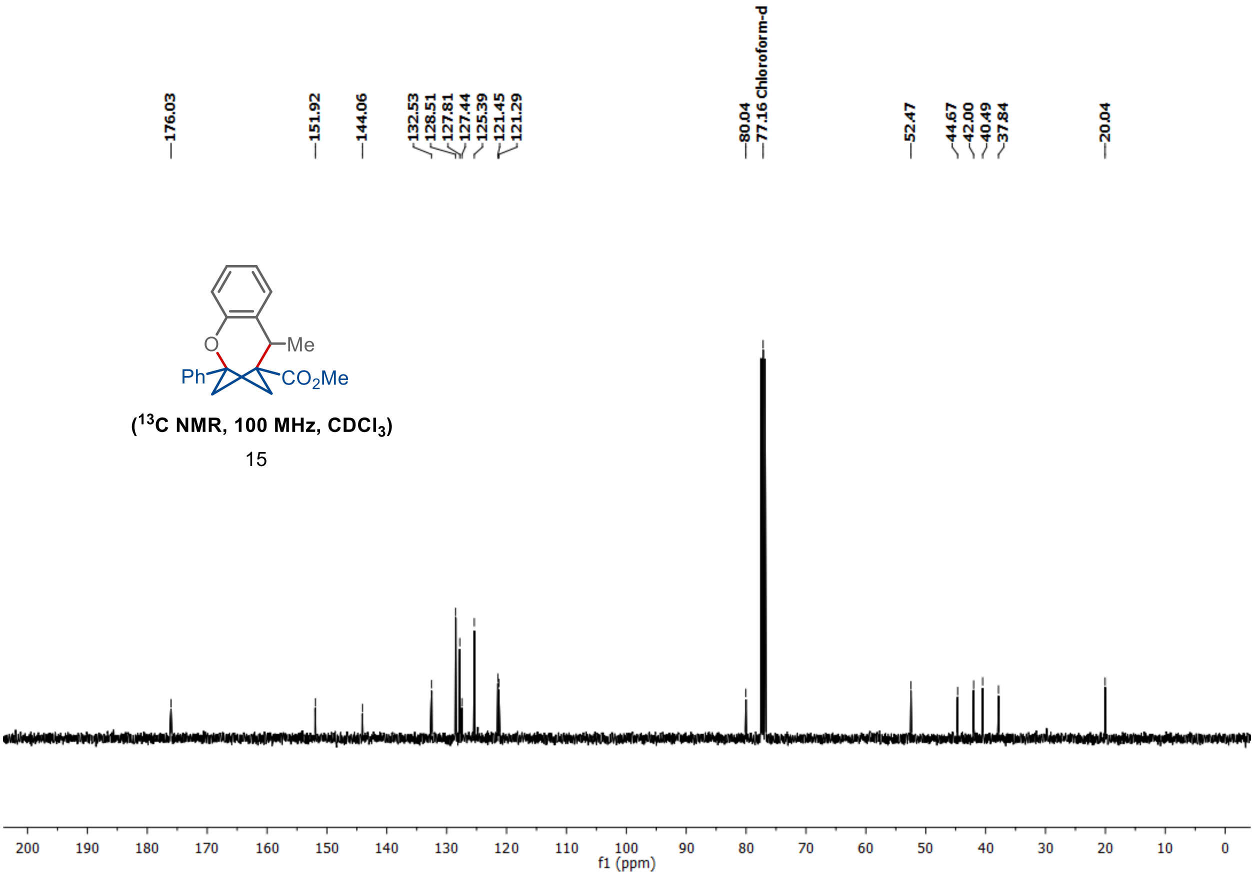
176.03
151.92
144.06
132.53 128.51 127.81 127.44 125.39 121.45 121.29
80.04 77.16 Chloroform-d
52.47
44.67 42.00 40.49 37.84
20.04
O
Me
Ph
CO2Me
(13C NMR, 100 MHz, CDCl3)
15
200 190 180 170 160 150 140 130 120 110 100 90 80 70 60 50 40 30 20 10 0
f1 (ppm)

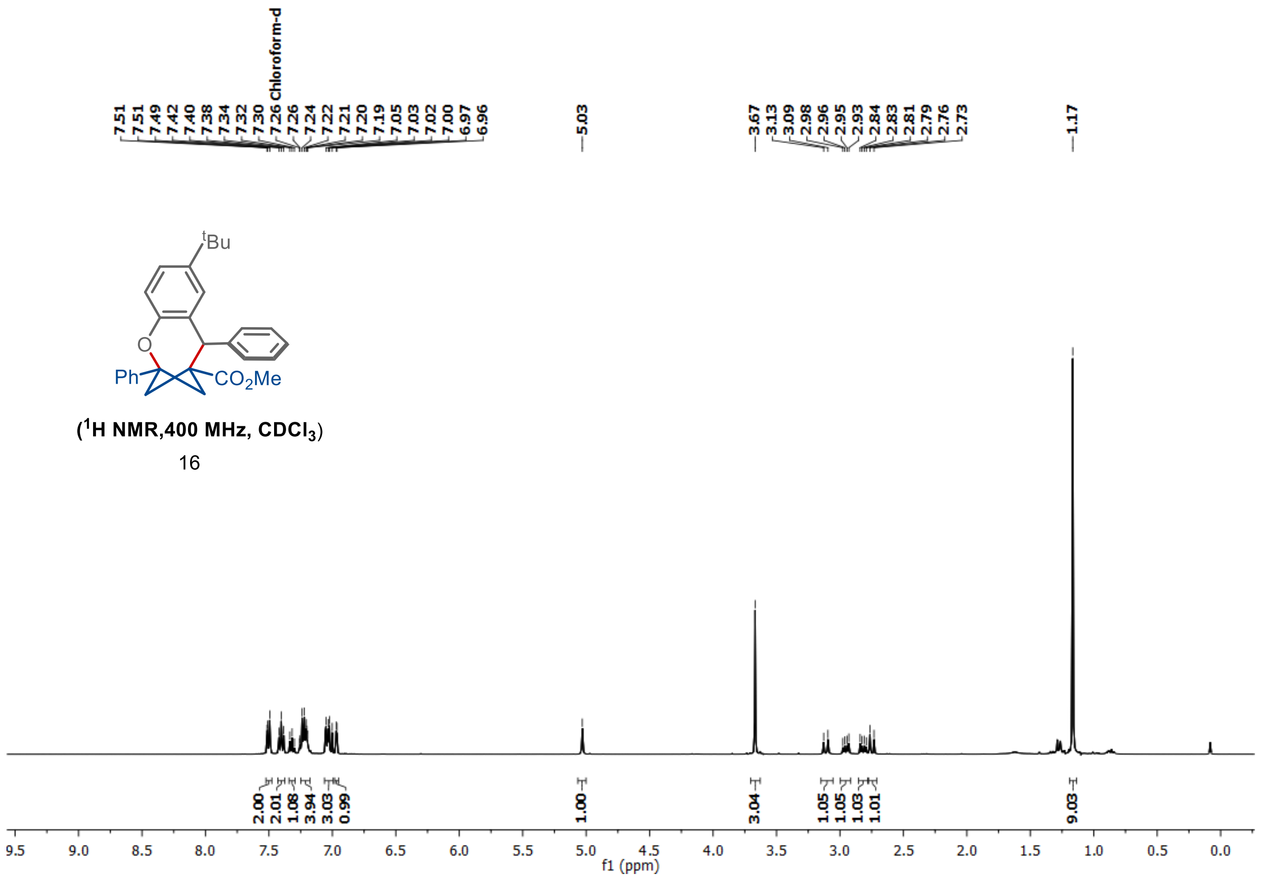

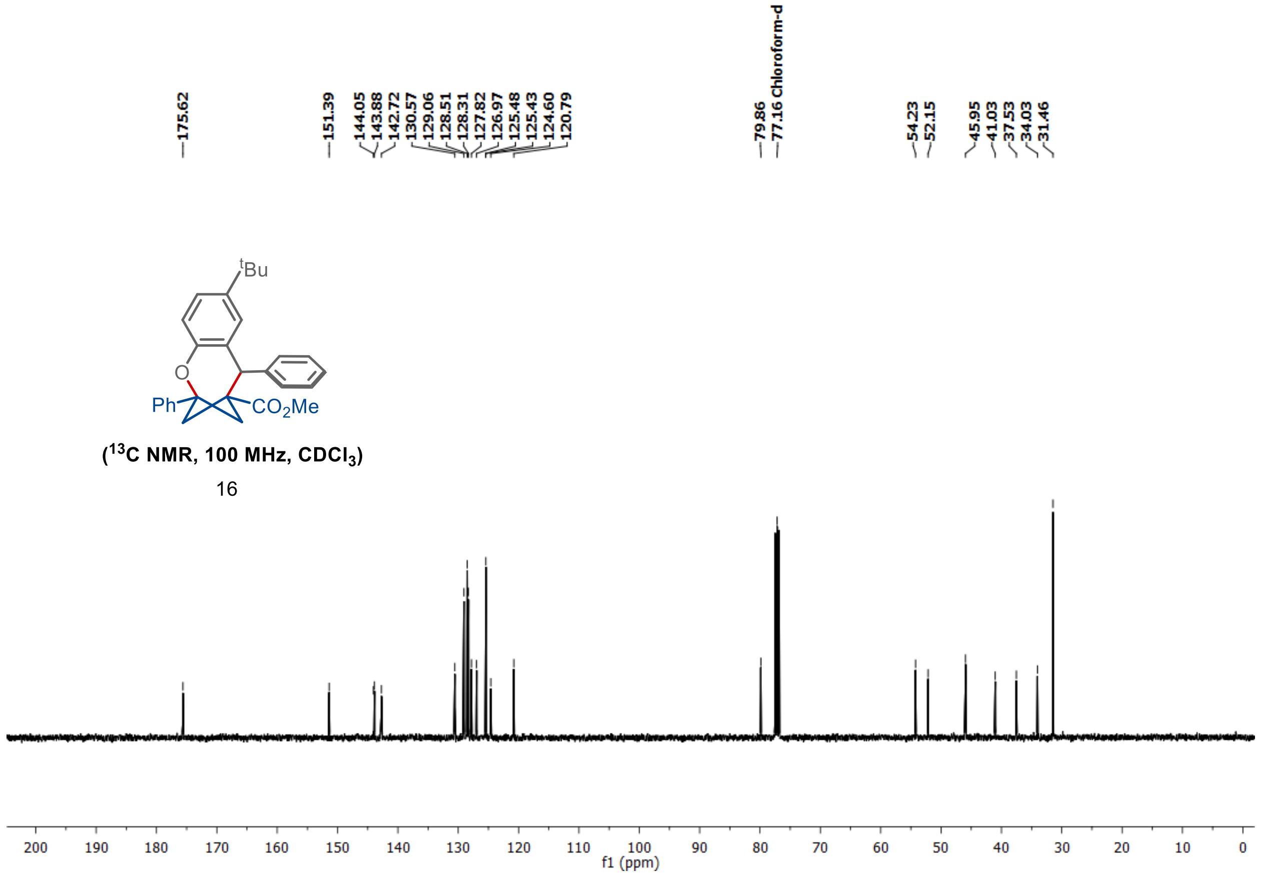

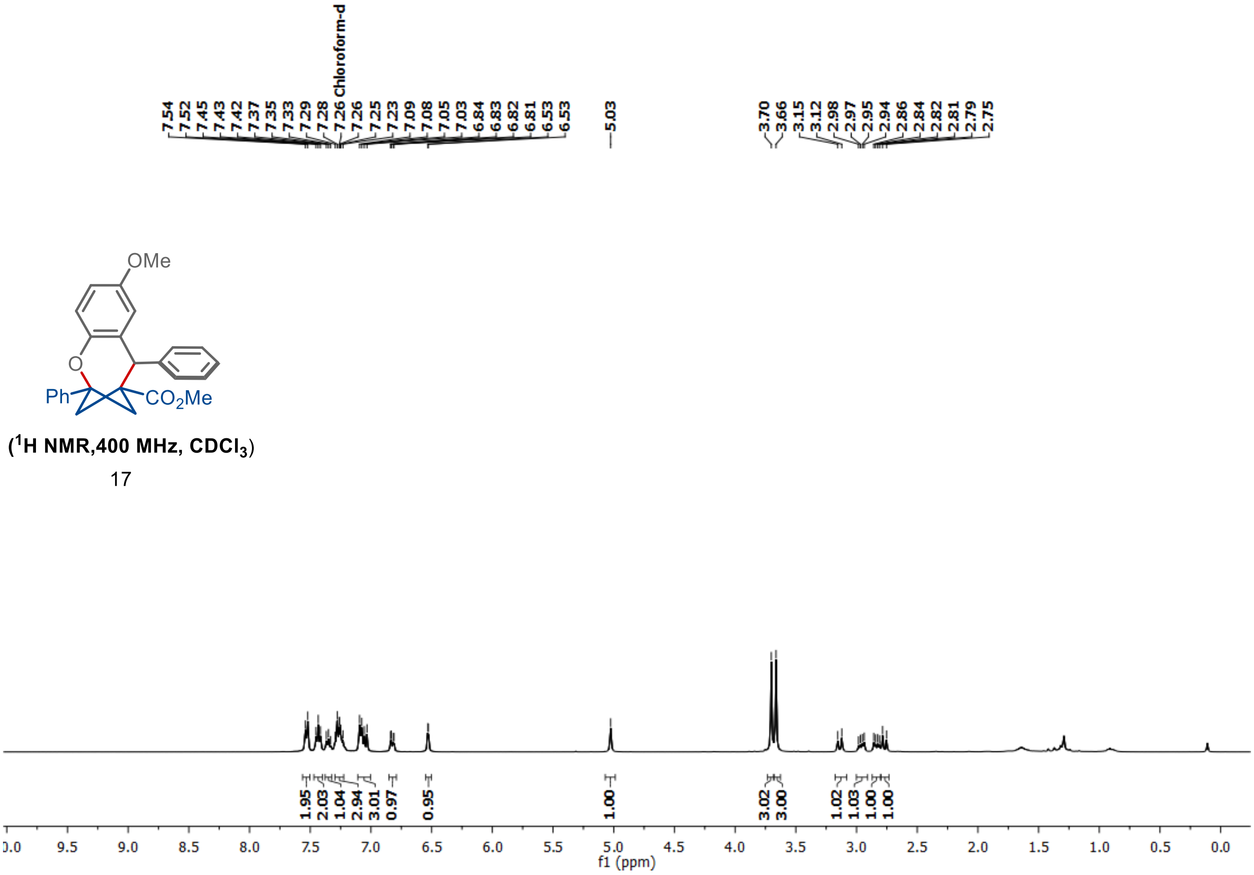

(^{1}H NMR,400 MHz, $CDCl_3$)

17

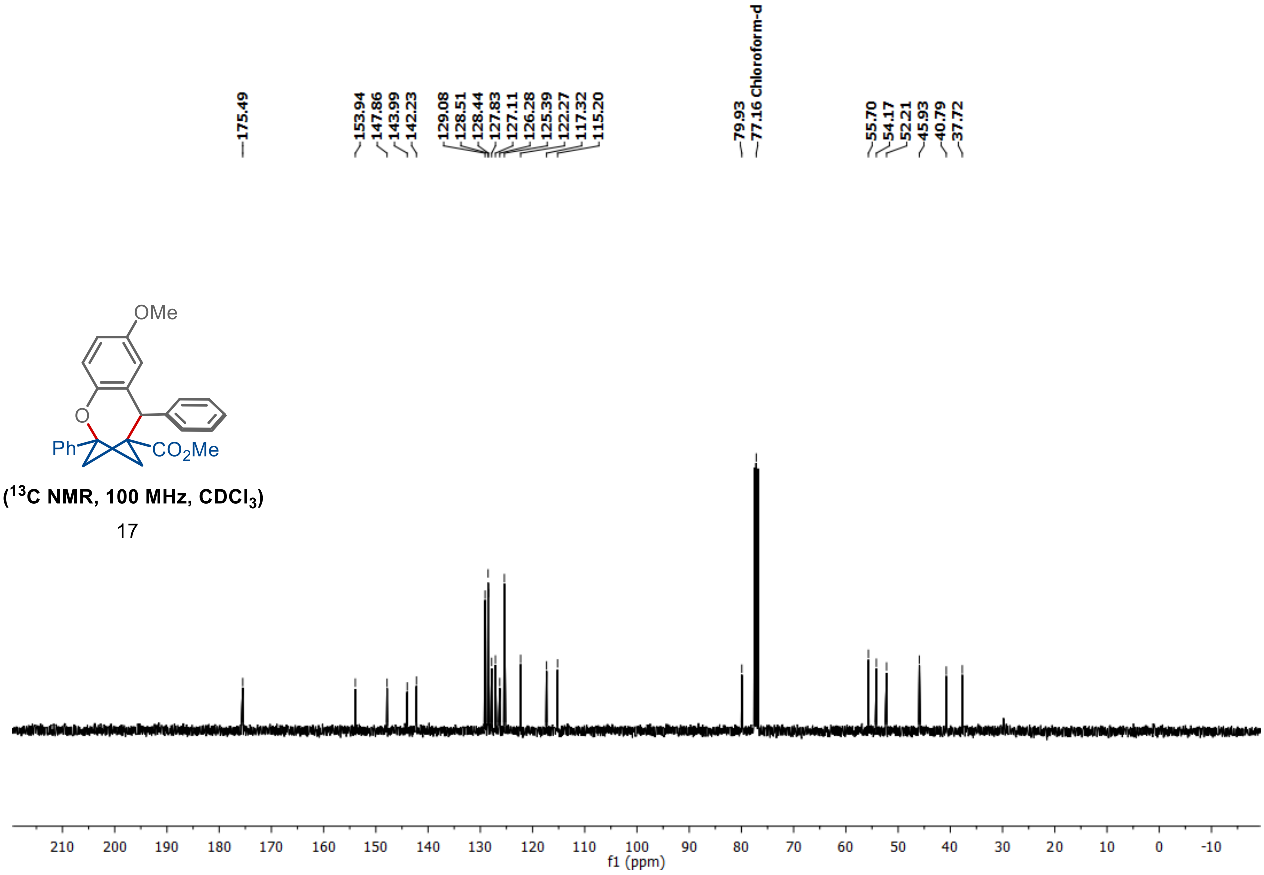

(^{13}C NMR, 100 MHz, $CDCl_3$)

17

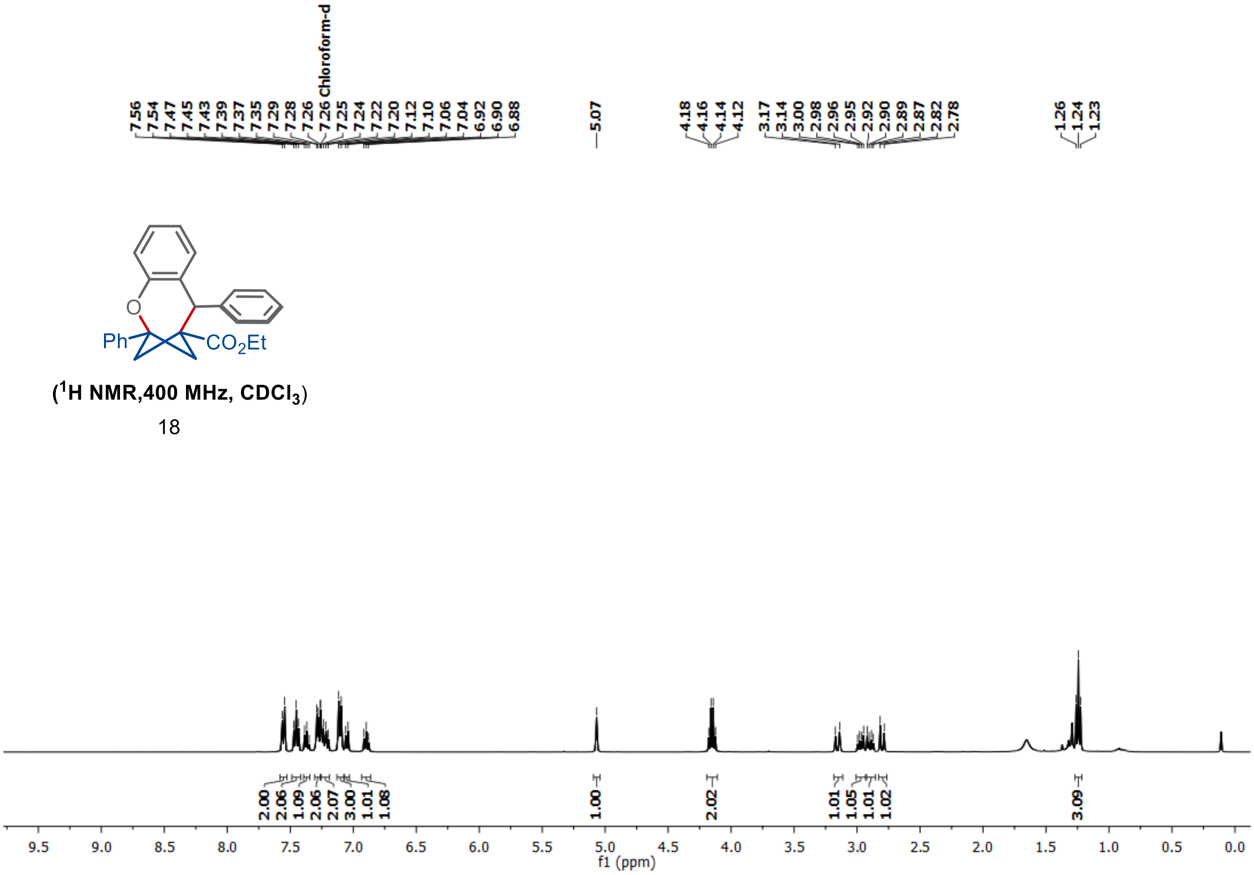

(¹H NMR,400 MHz, CDCl$_3$)

18

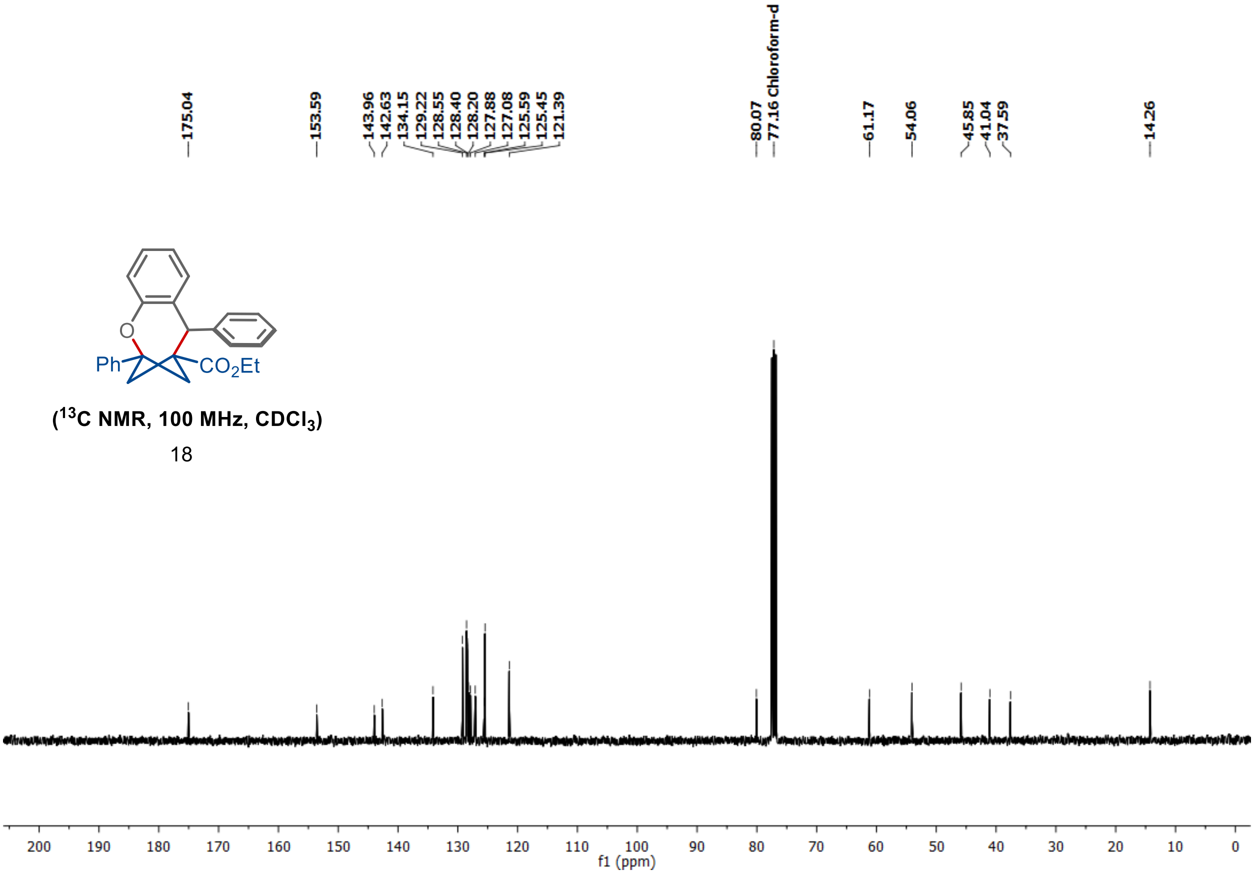

(¹³C NMR, 100 MHz, CDCl$_3$)

18

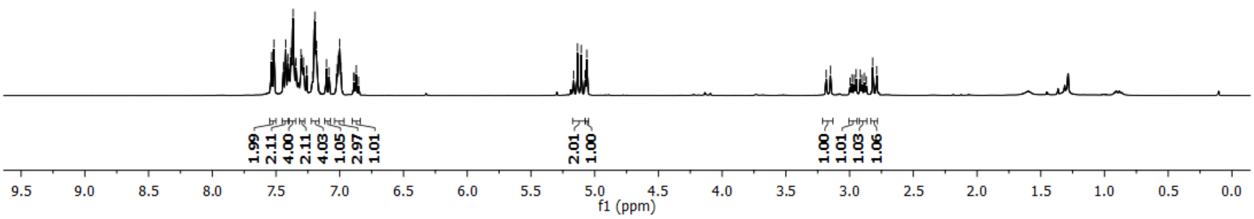

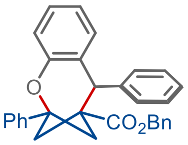

(^{1}H NMR,400 MHz, $CDCl_3$)

19

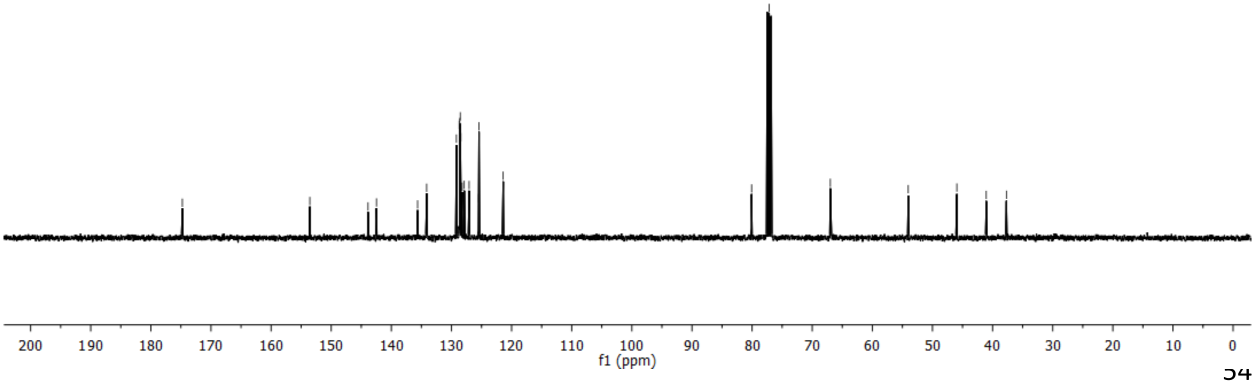

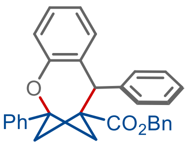

(^{13}C NMR, 100 MHz, $CDCl_3$)

19

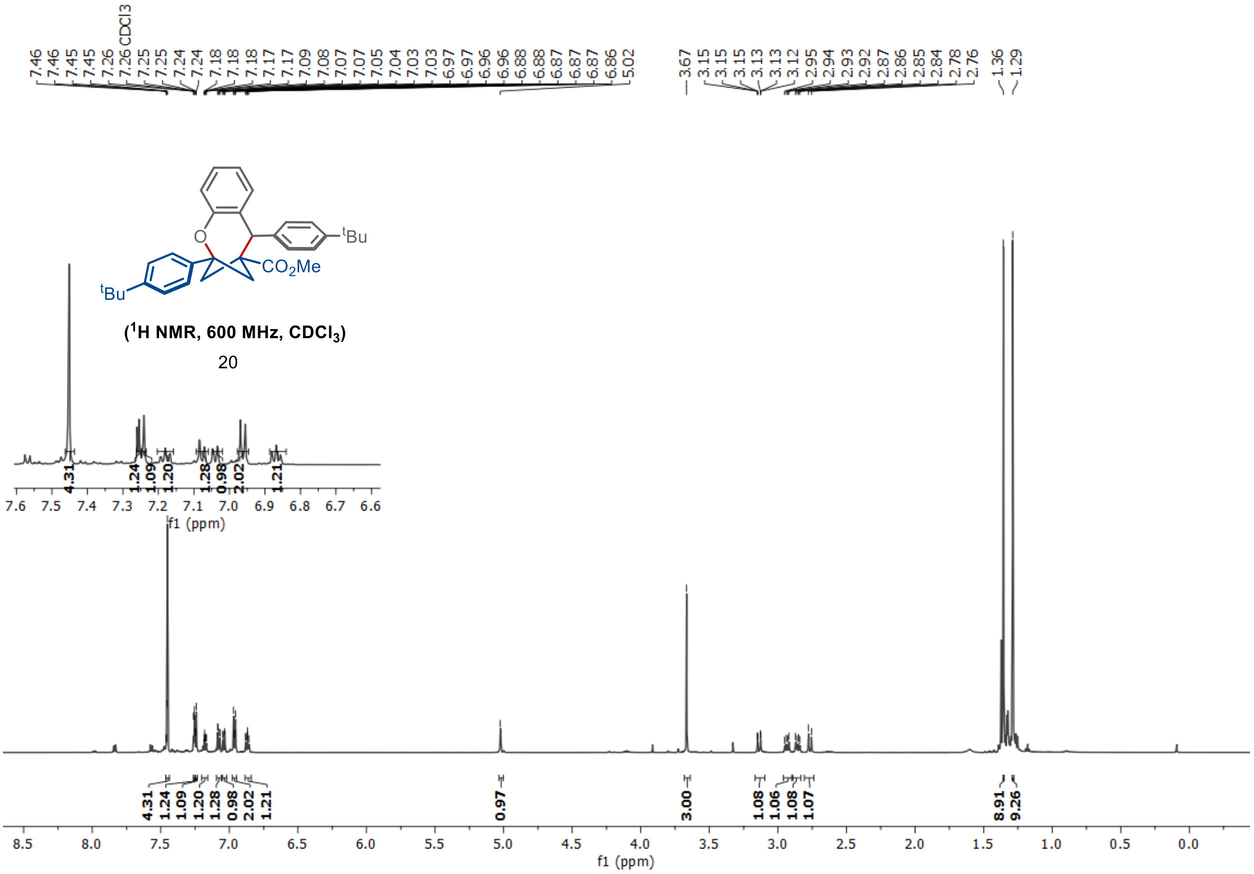
7.46 7.46 7.45 7.45 7.26 7.26 CDCl3 7.25 7.25 7.24 7.24 7.18 7.18 7.18 7.18 7.17 7.17 7.09 7.08 7.07 7.07 7.05 7.04 7.03 7.03 6.97 6.97 6.96 6.96 6.88 6.88 6.87 6.87 6.87 6.86 5.02
3.67 3.15 3.15 3.15 3.13 3.13 3.12 2.95 2.94 2.93 2.92 2.87 2.86 2.85 2.84 2.78 2.76 1.36 1.29
O
tBu
CO2Me
tBu
(1H NMR, 600 MHz, CDCl3)
20
4.31 1.24 1.09 1.20 1.28 0.98 2.02 1.21
0.97
3.00
1.08 1.06 1.08 1.07
8.91 9.26
f1 (ppm)

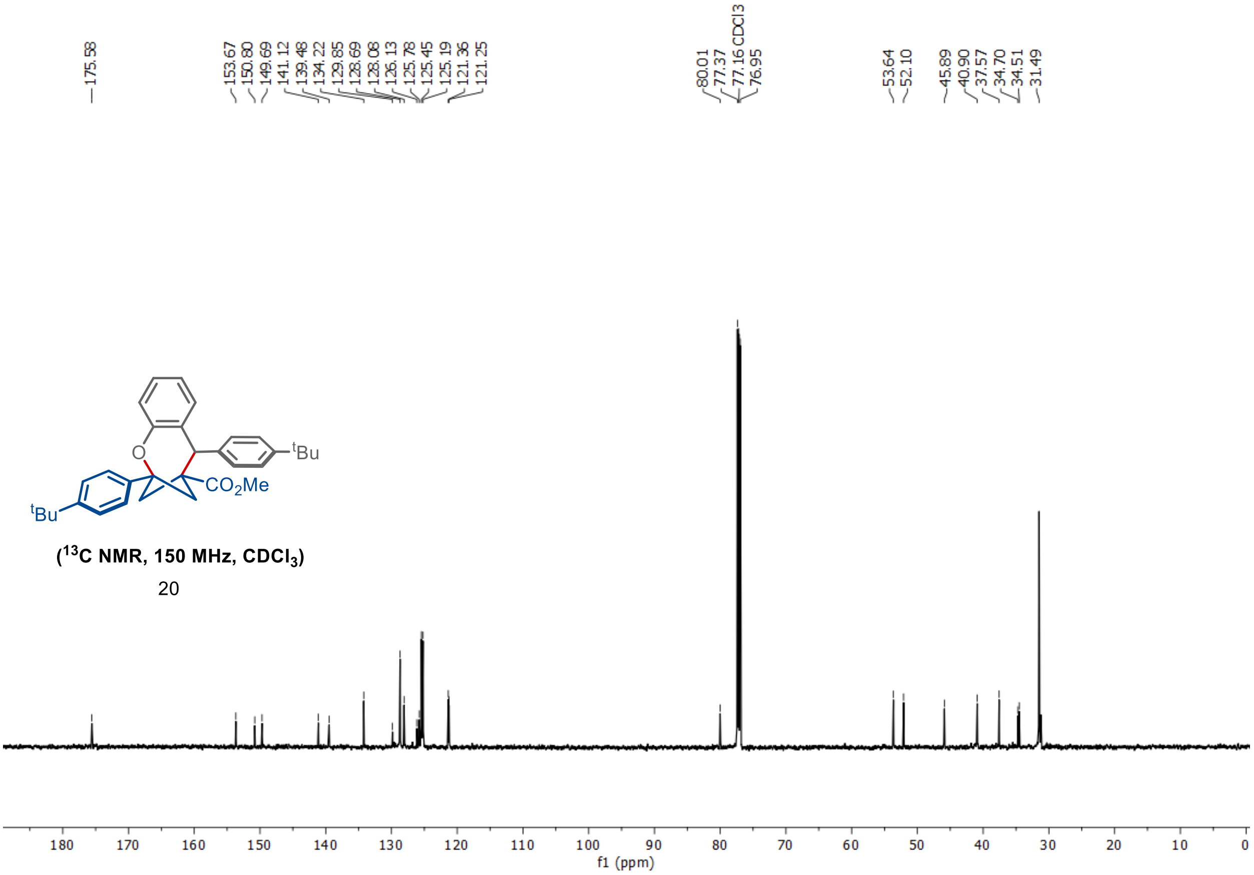
175.58
153.67 150.80 149.69 141.12 139.48 134.22 129.85 128.69 128.08 126.13 125.78 125.45 125.19 121.36 121.25
80.01 77.37 77.16 CDCl3 76.95
53.64 52.10
45.89 40.90 37.57 34.70 34.51 31.49
O
tBu
CO2Me
tBu
(13C NMR, 150 MHz, CDCl3)
20
f1 (ppm)

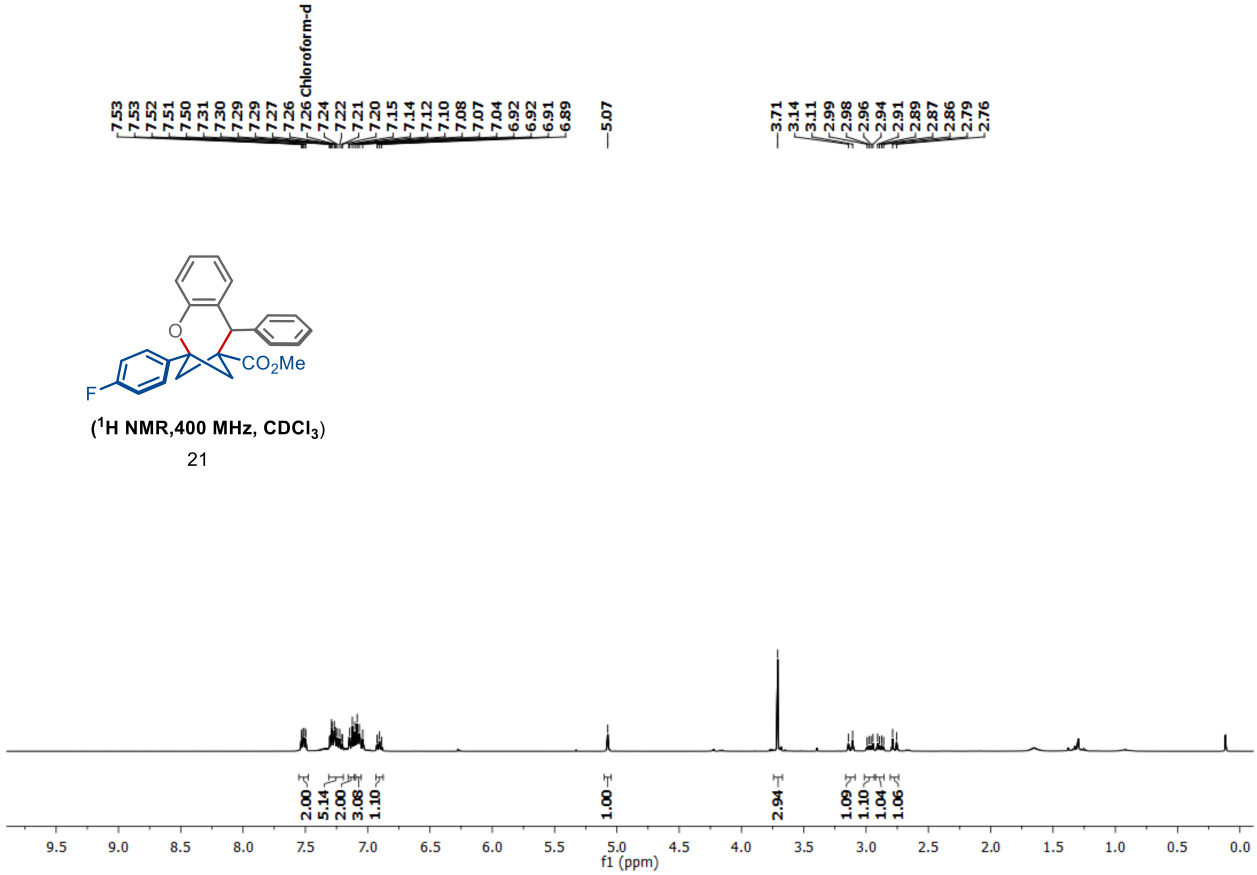
7.53 7.53 7.52 7.51 7.50 7.31 7.30 7.29 7.29 7.27 7.26 7.26 Chloroform-d 7.24 7.22 7.21 7.20 7.15 7.14 7.12 7.10 7.08 7.07 7.04 6.92 6.91 6.89
5.07
3.71 3.14 3.11 2.99 2.98 2.96 2.94 2.91 2.89 2.87 2.86 2.79 2.76
O
CO_2Me
F
(^{1}H NMR,400 MHz, $CDCl_3$)
21
2.00 5.14 2.00 3.08 1.10 1.00 2.94 1.09 1.10 1.04 1.06
f1 (ppm)

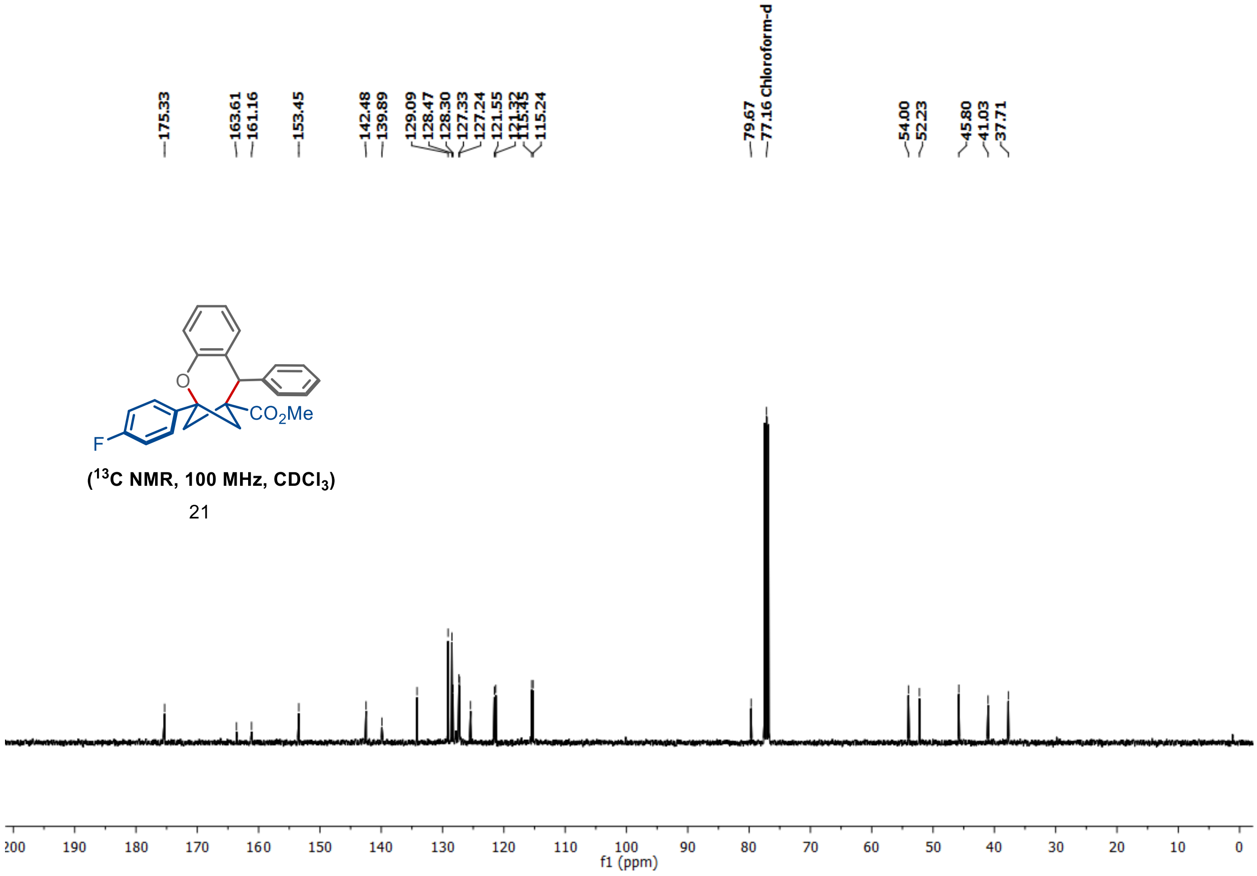
175.33
163.61 161.16
153.45
142.48 139.89
129.09 128.47 128.30 127.33 127.24 121.55 121.32 115.45 115.24
79.67 77.16 Chloroform-d
54.00 52.23
45.80 41.03 37.71
O
CO_2Me
F
(^{13}C NMR, 100 MHz, $CDCl_3$)
21
f1 (ppm)

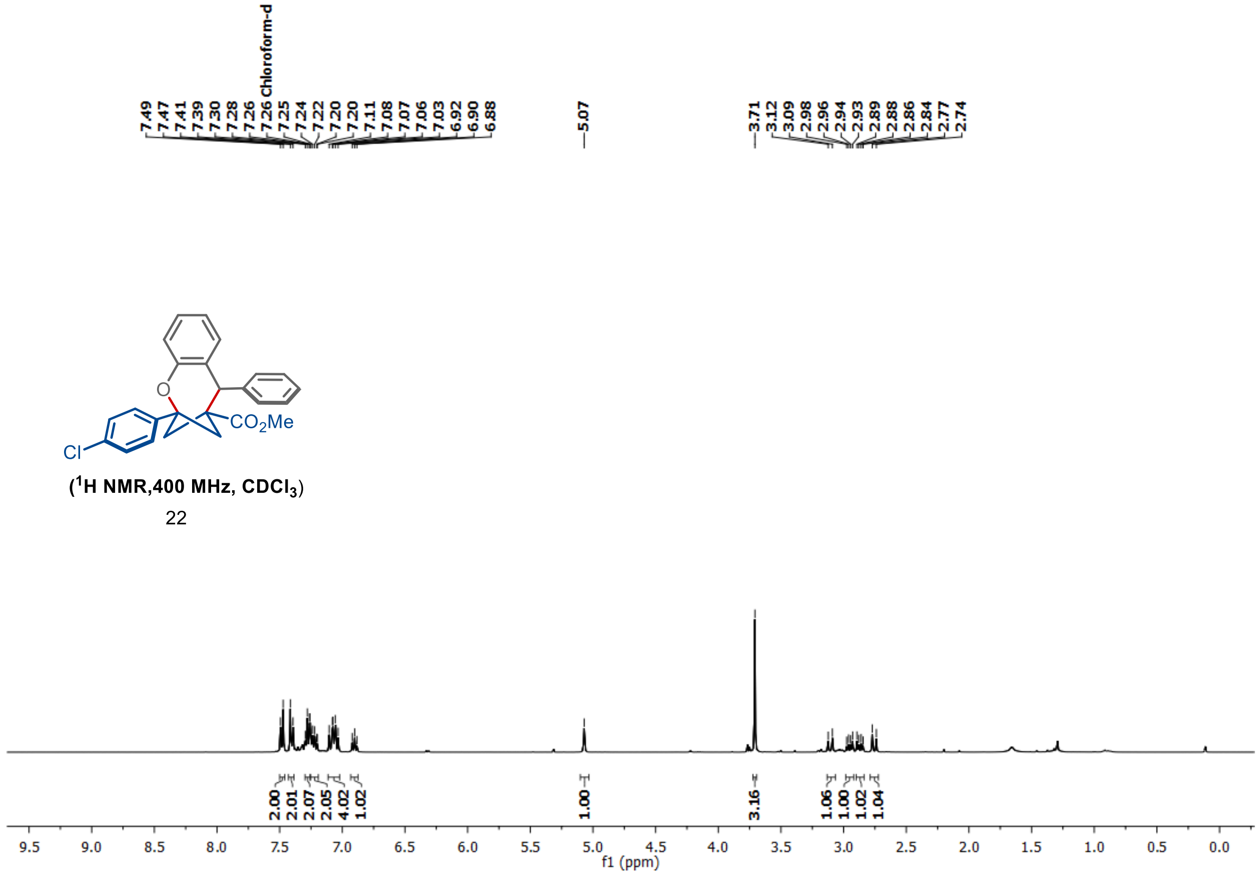

(^{1}H NMR,400 MHz, $CDCl_3$)

22

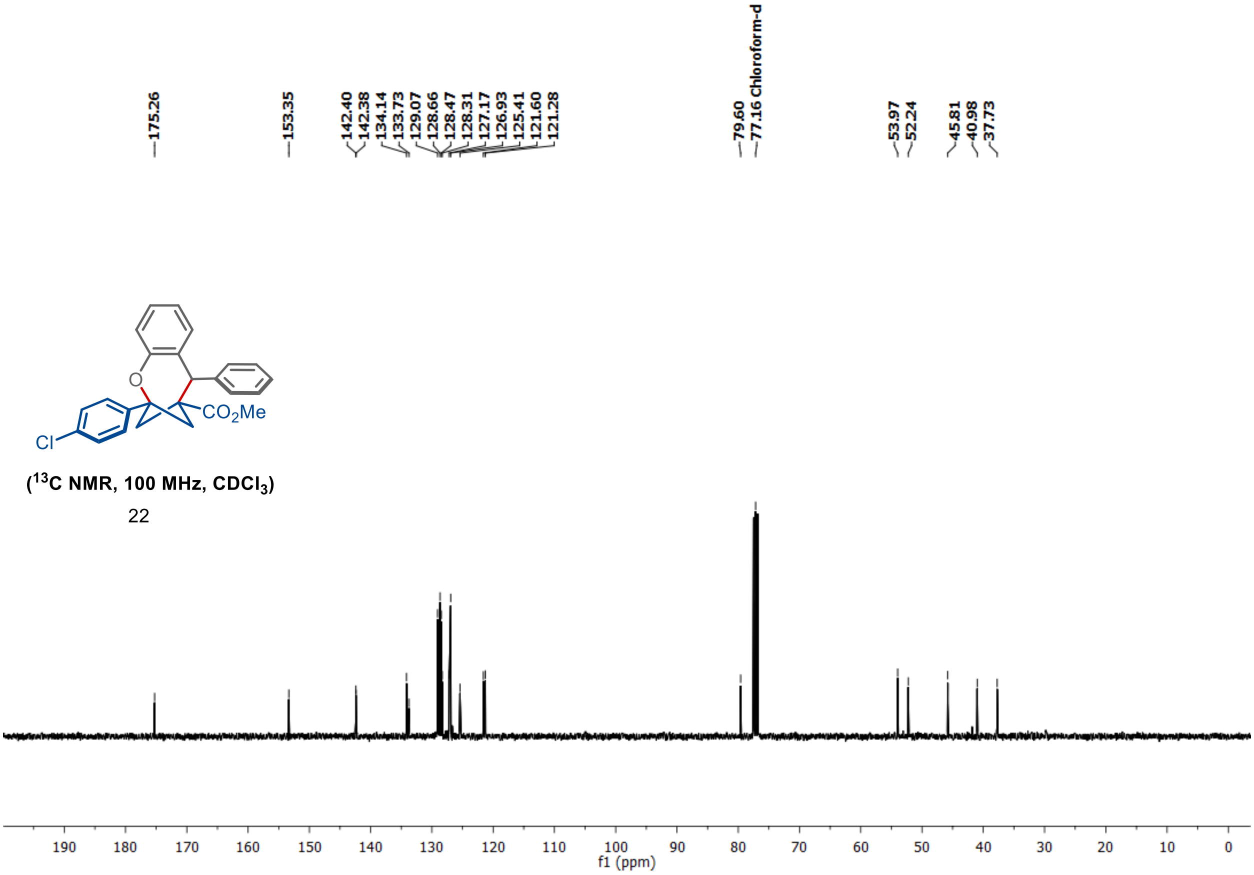

(^{13}C NMR, 100 MHz, $CDCl_3$)

22

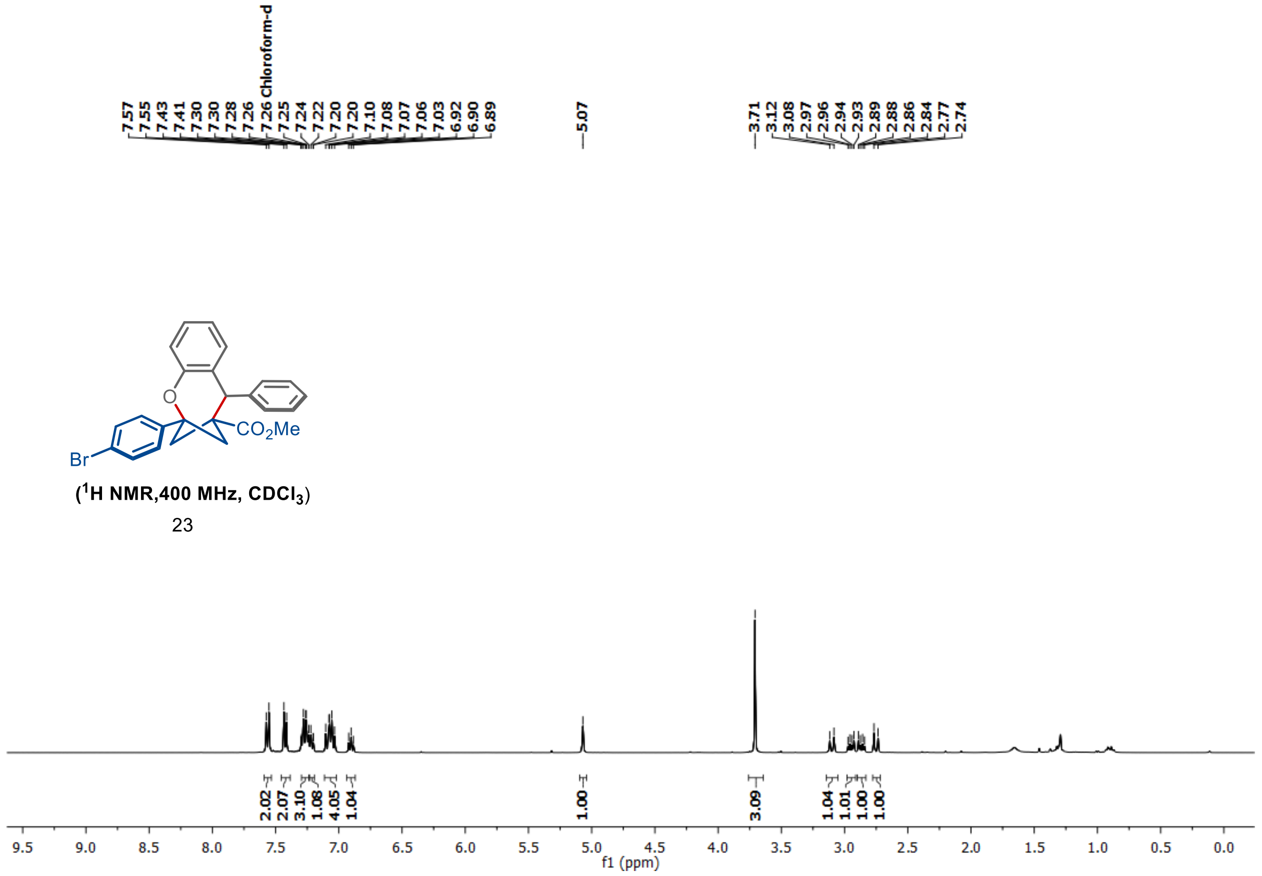

(¹H NMR,400 MHz, $CDCl_3$)

23

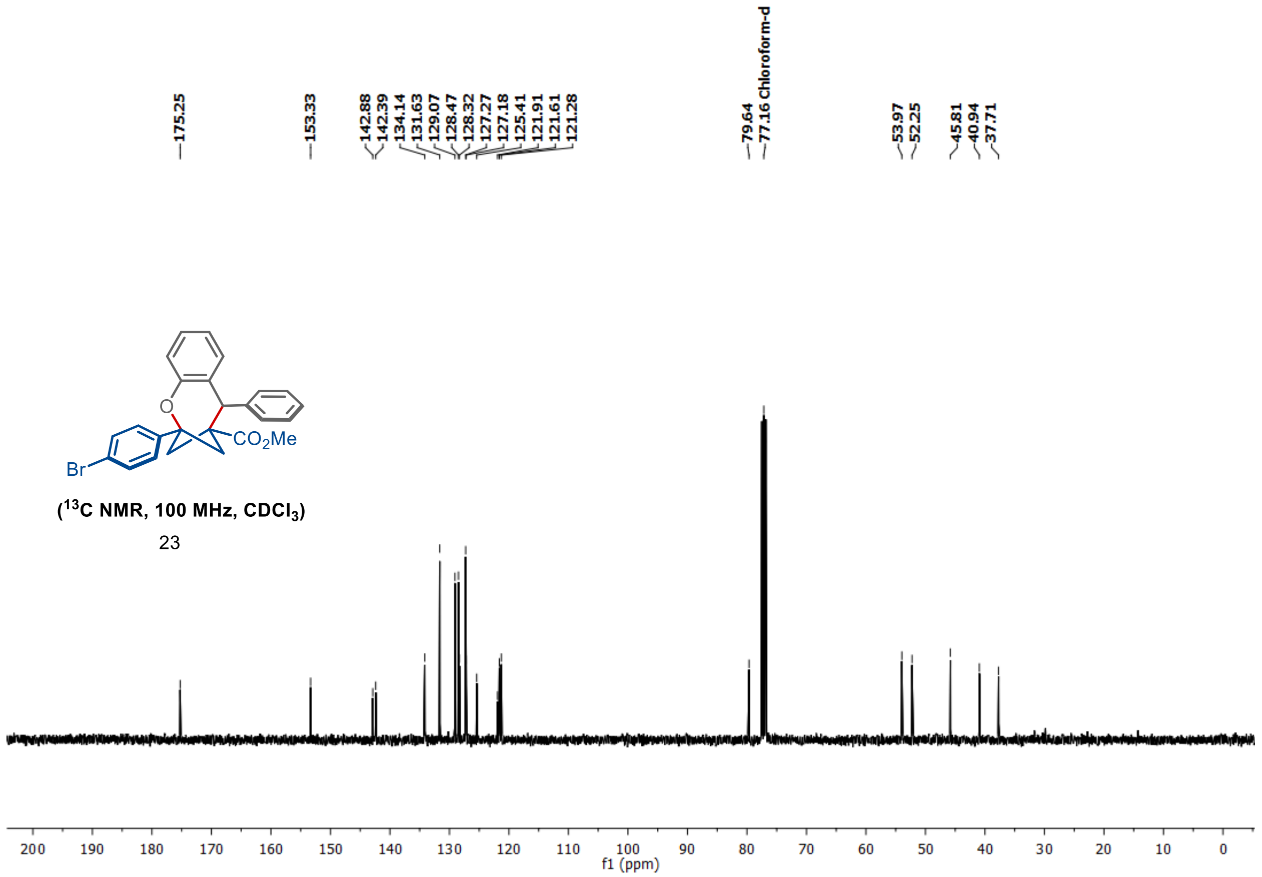

(¹³C NMR, 100 MHz, $CDCl_3$)

23

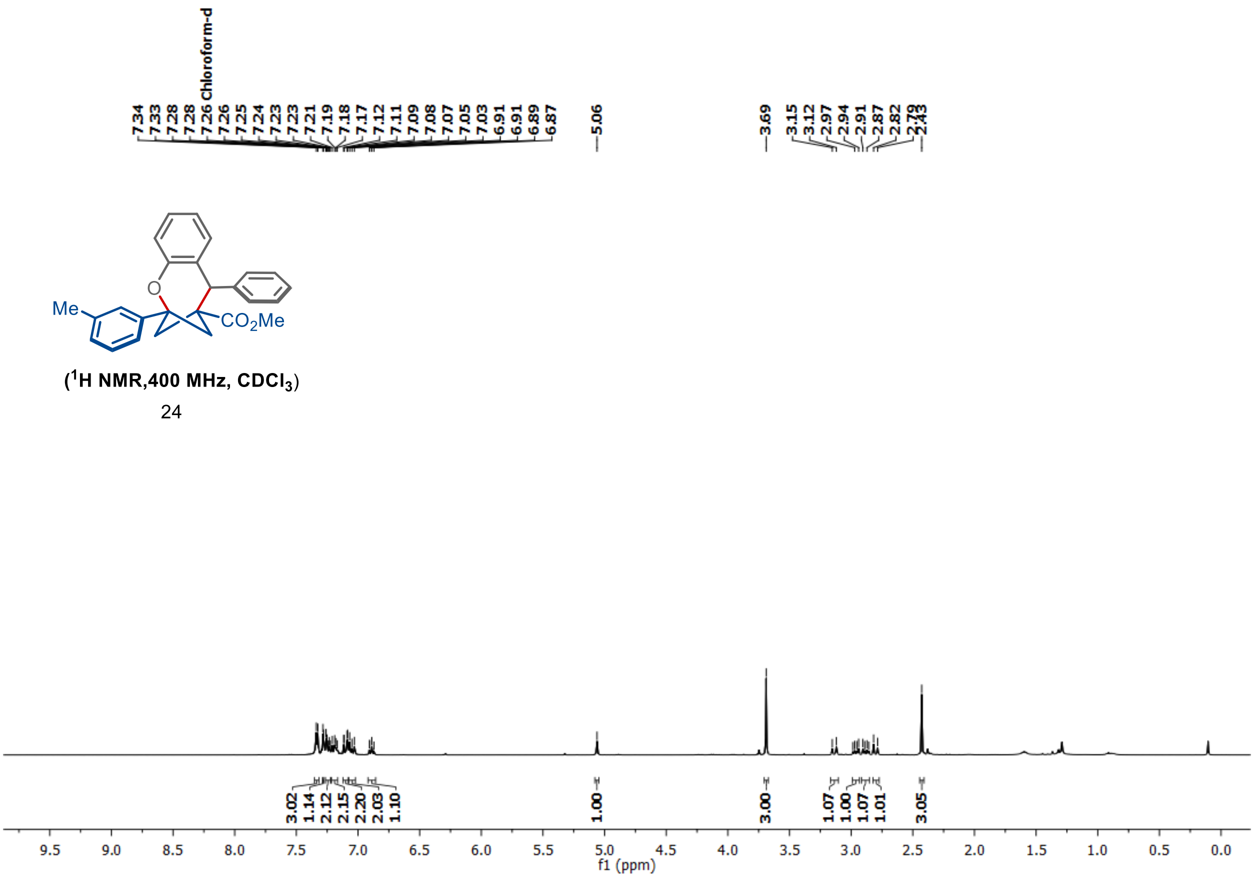

(${}^{1}H$ NMR,400 MHz, $CDCl_3$)

24

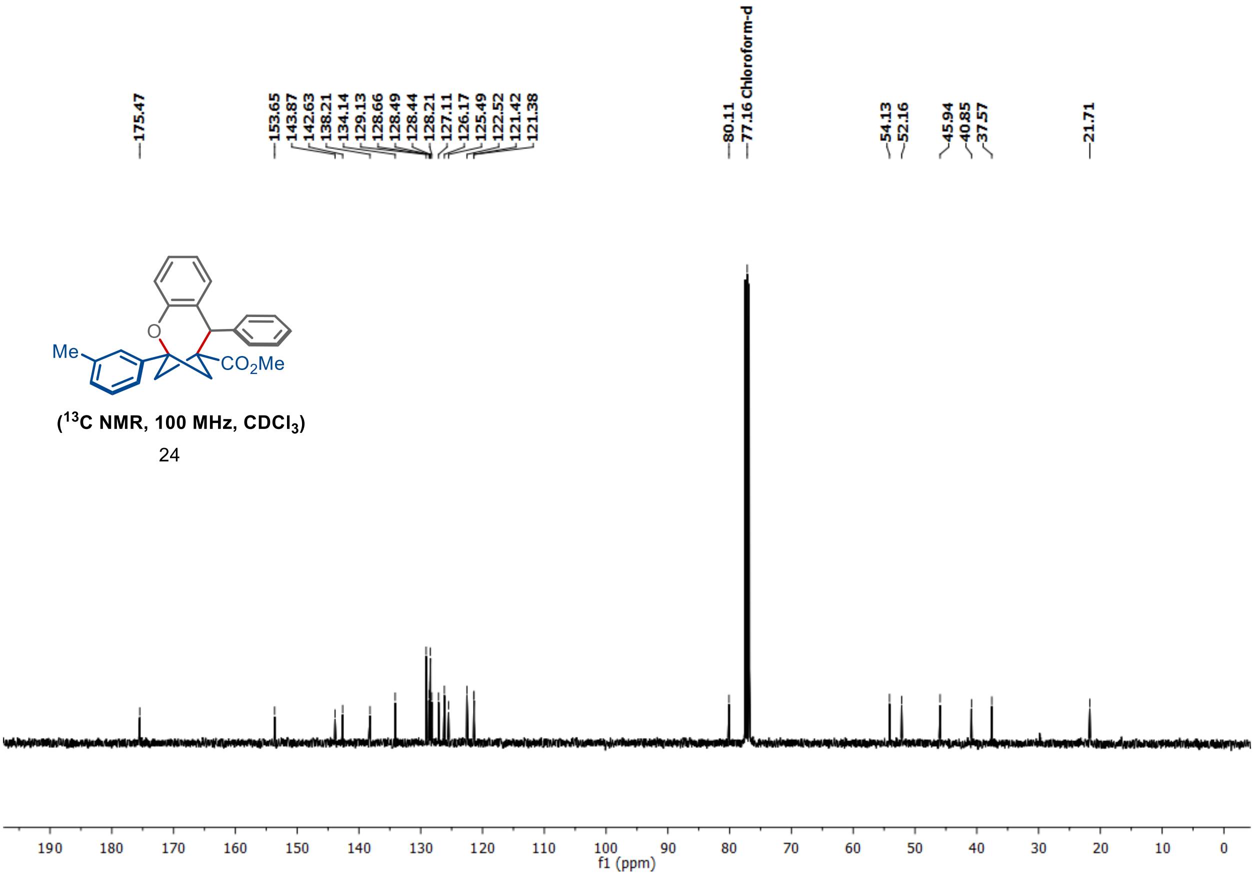

(${}^{13}C$ NMR, 100 MHz, $CDCl_3$)

24

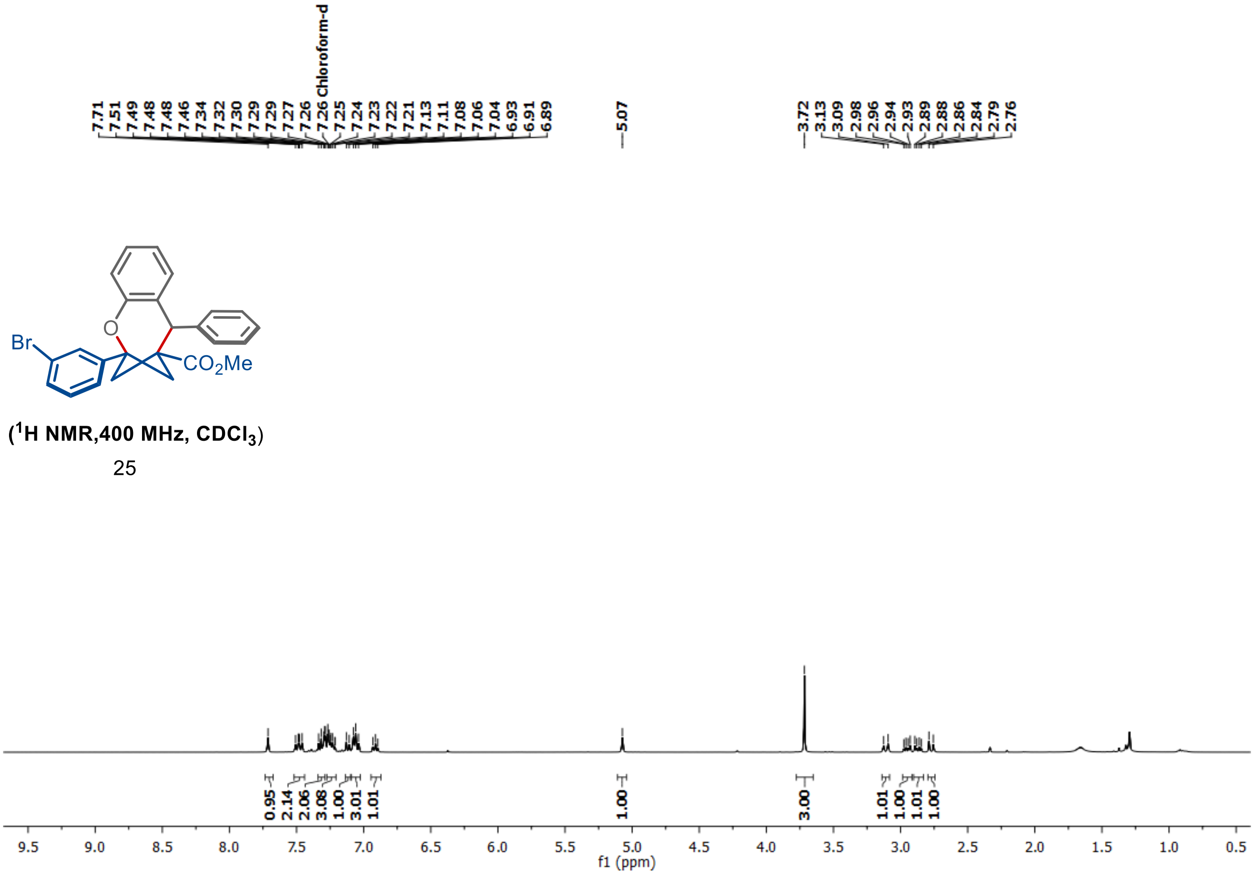

(^{1}H NMR,400 MHz, $CDCl_3$)

25

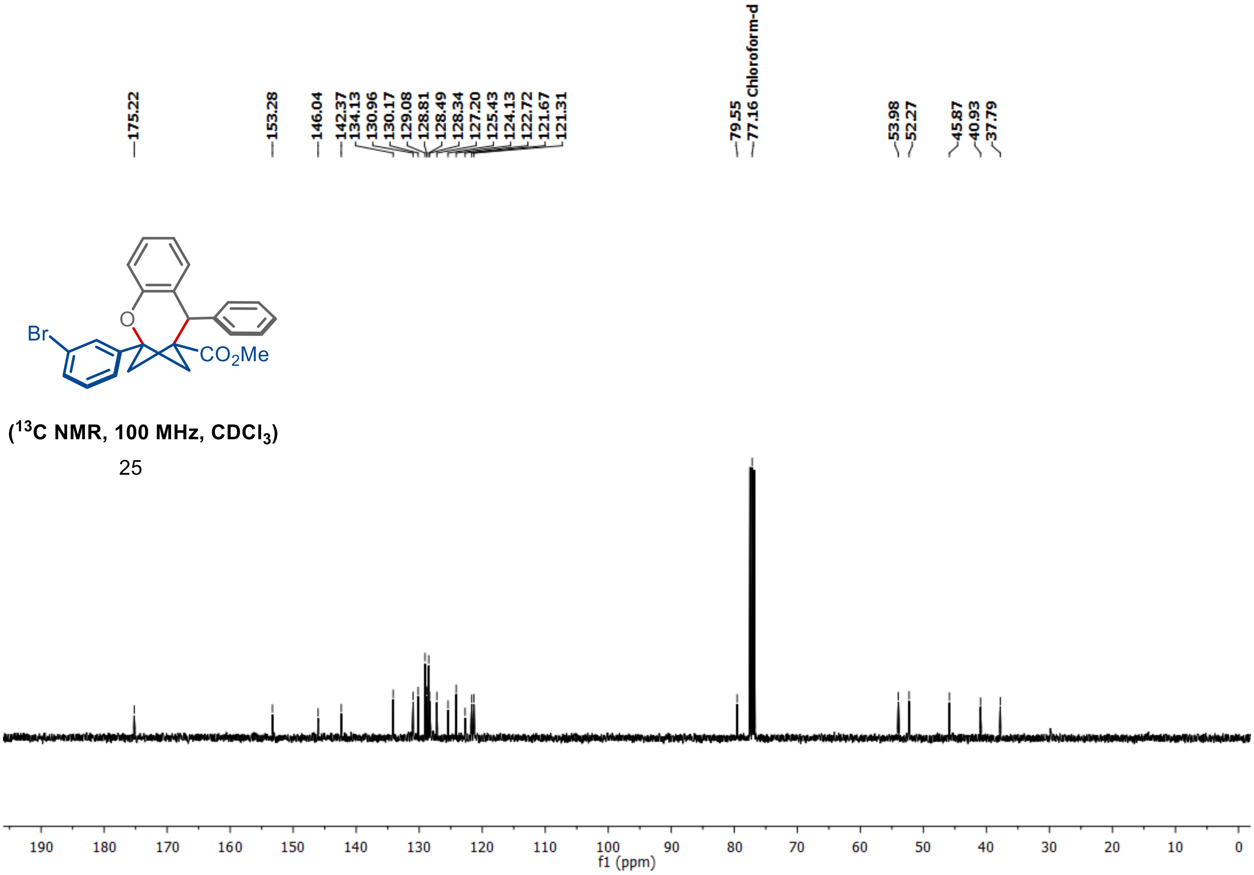

(^{13}C NMR, 100 MHz, $CDCl_3$)

25

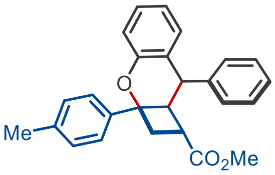

(^{1}H NMR,400 MHz, $CDCl_3$)

26

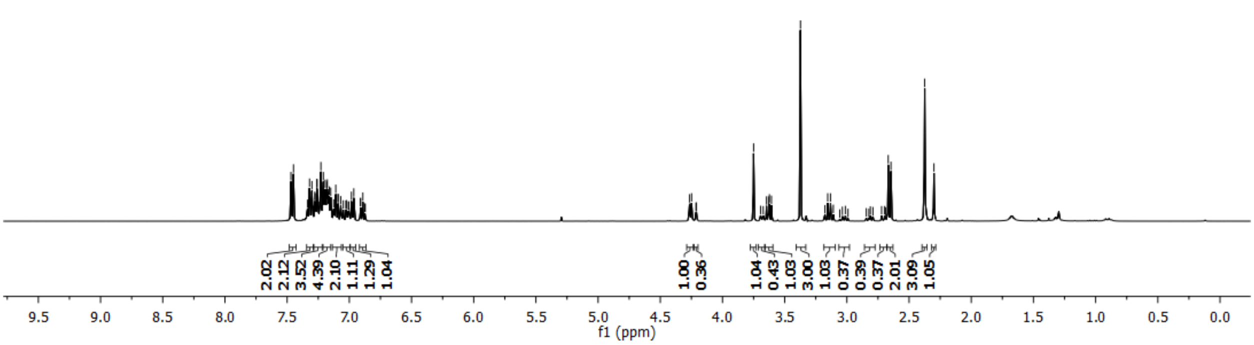

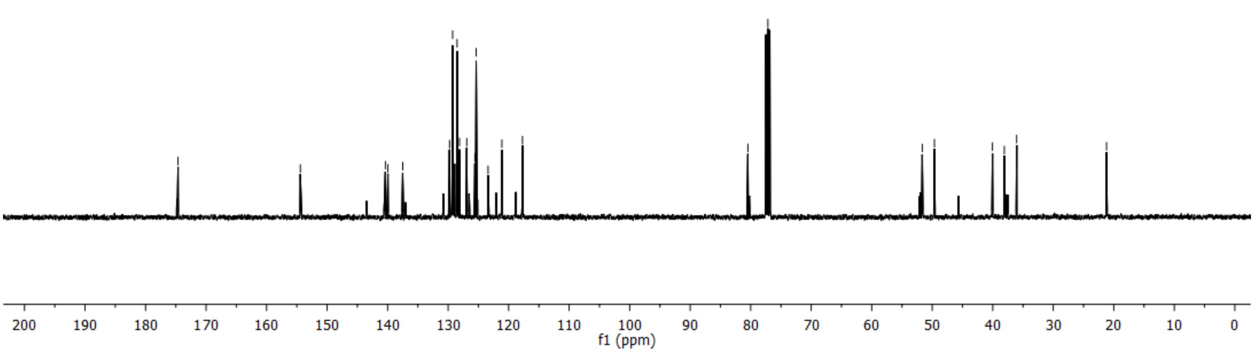

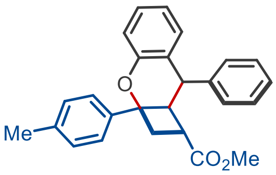

(^{13}C NMR, 100 MHz, $CDCl_3$)

26

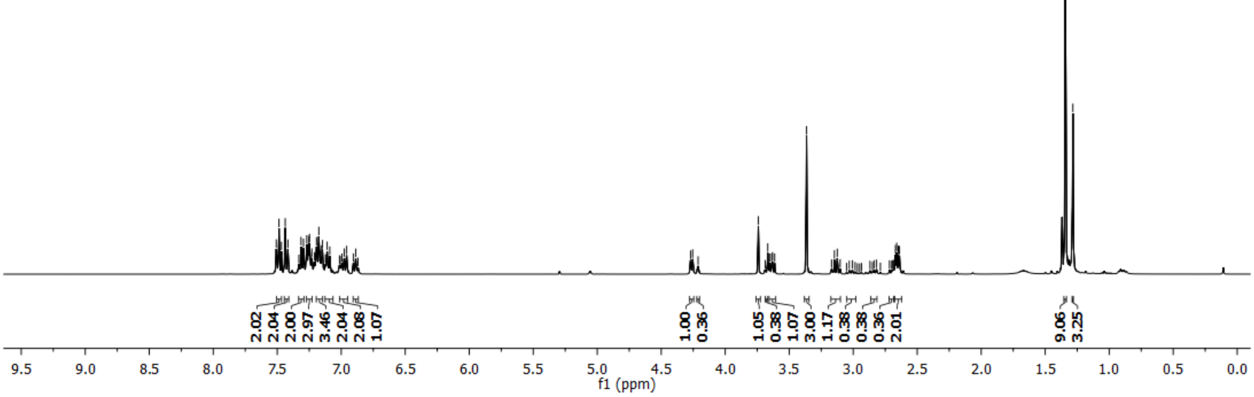

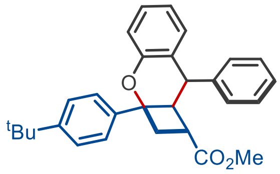

(^{1}H NMR,400 MHz, $CDCl_3$)

27

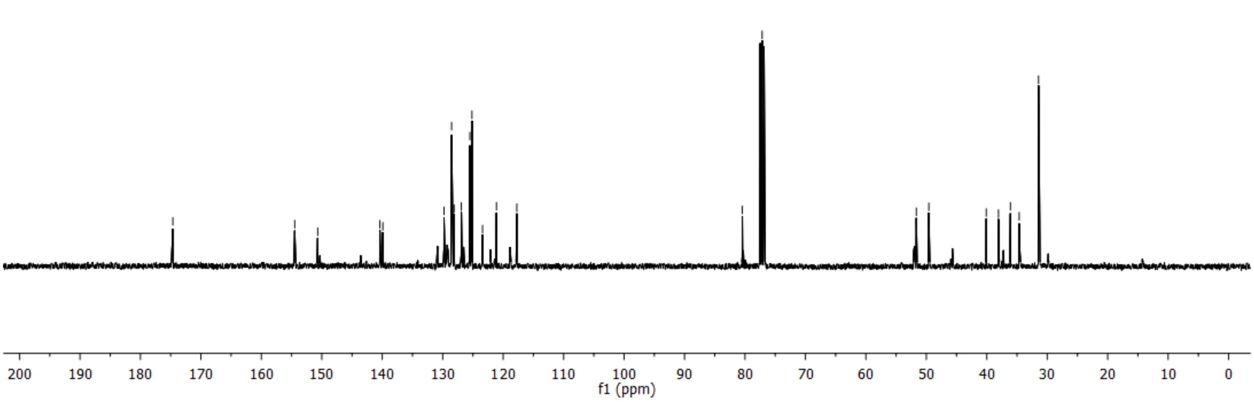

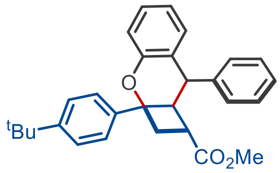

(^{13}C NMR, 100 MHz, $CDCl_3$)

27

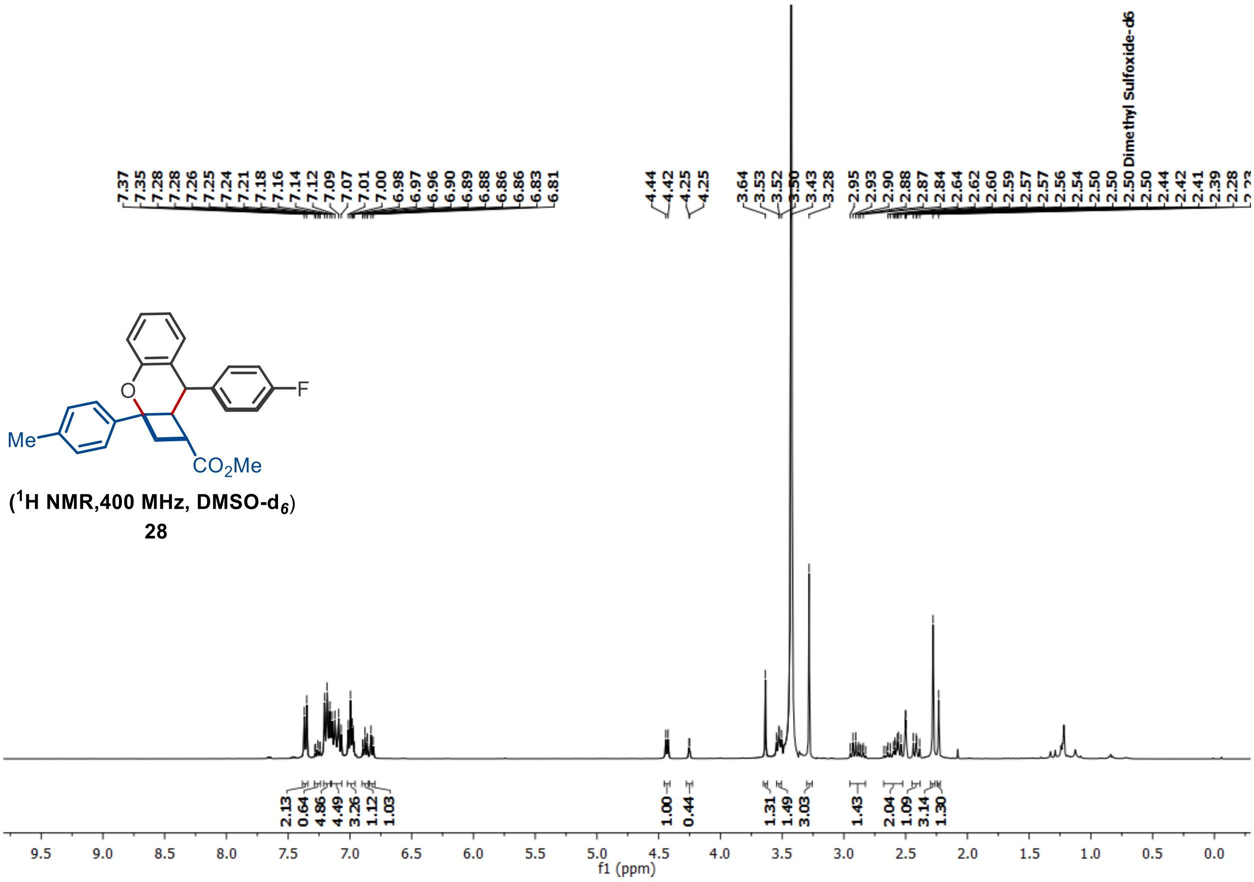
Me
O
F
CO2Me
(1H NMR,400 MHz, DMSO-d6)
28
2.50 Dimethyl Sulfoxide-d6
f1 (ppm)

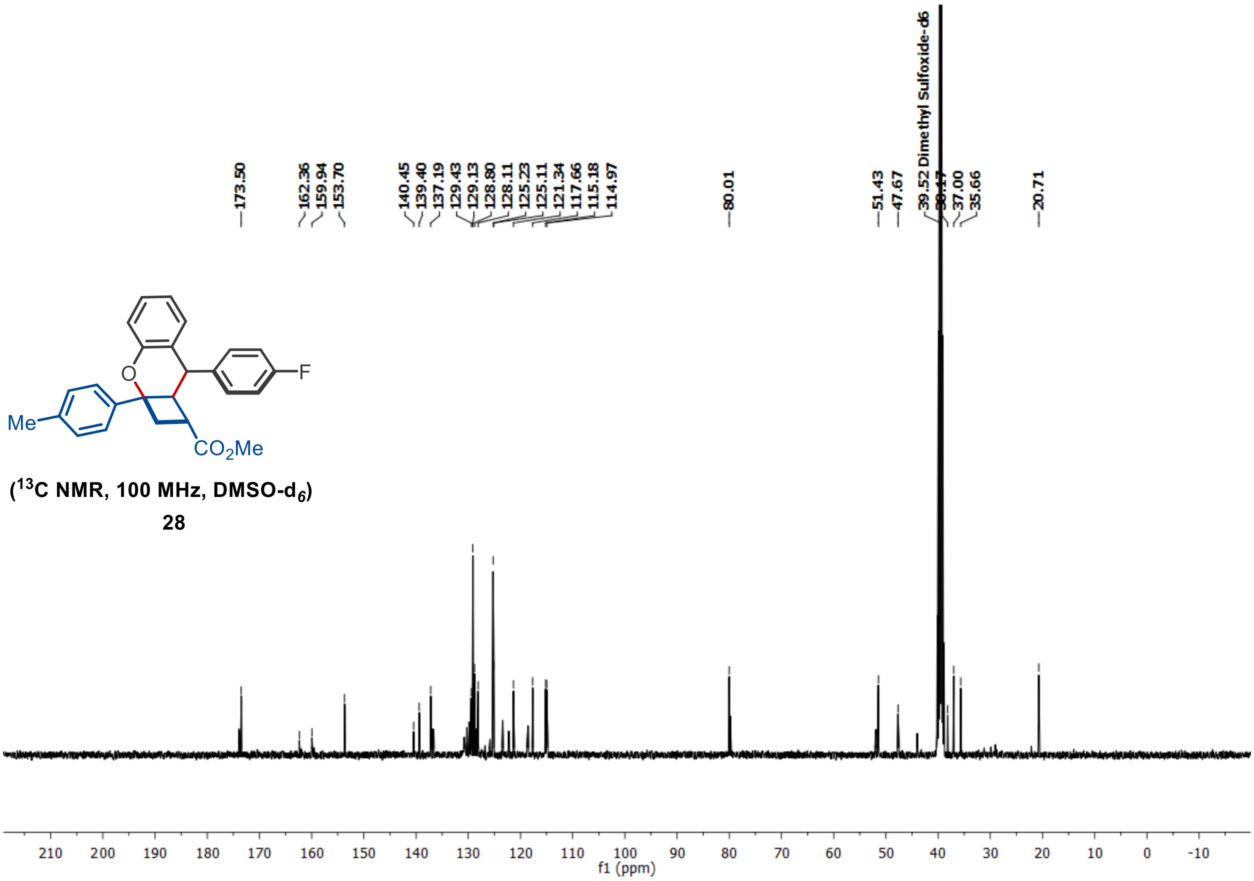
Me
O
F
CO2Me
(13C NMR, 100 MHz, DMSO-d6)
28
173.50
162.36
159.94
153.70
140.45
139.40
137.19
129.43
129.13
128.80
128.11
125.23
125.11
121.34
117.66
115.18
114.97
80.01
51.43
47.67
39.52 Dimethyl Sulfoxide-d6
37.00
35.66
20.71
f1 (ppm)

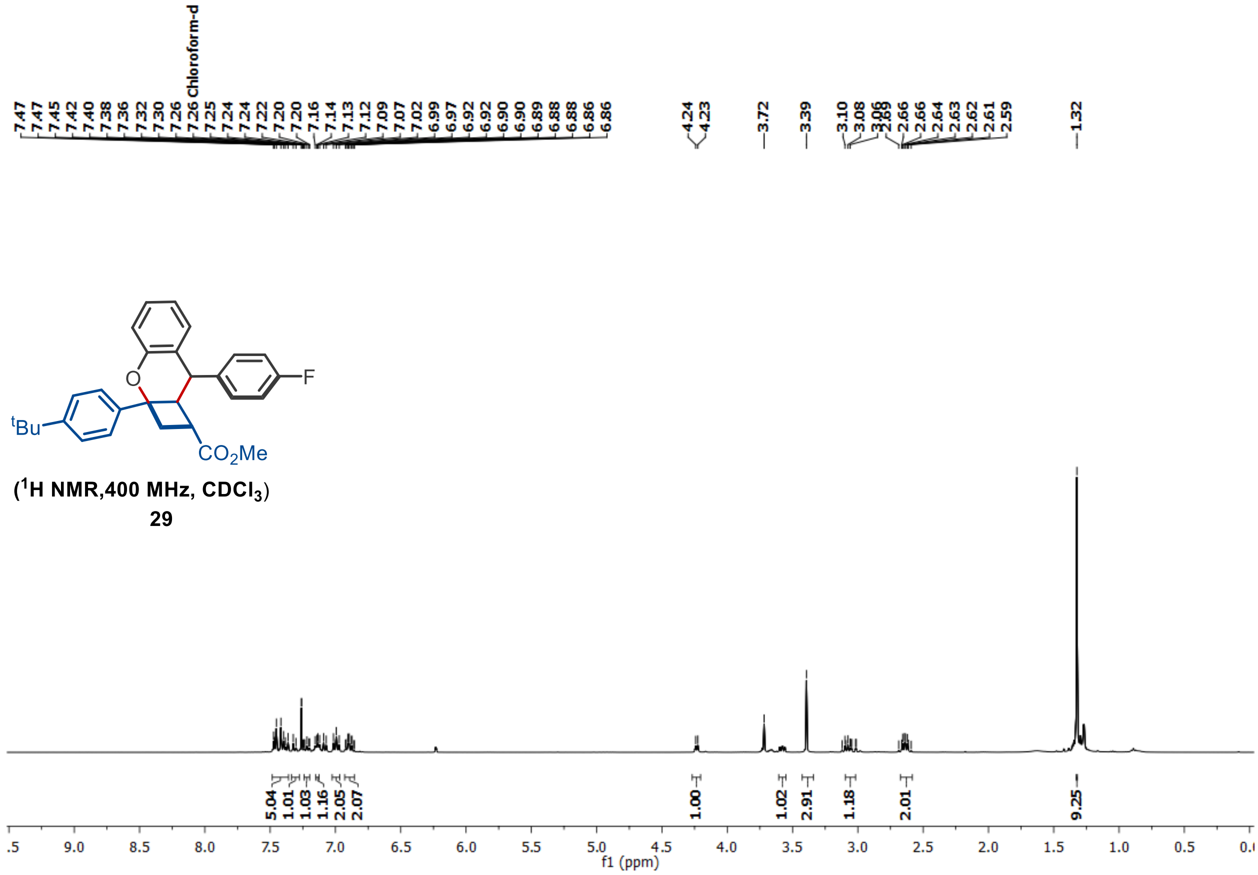

(^{1}H NMR,400 MHz, $CDCl_3$)

29

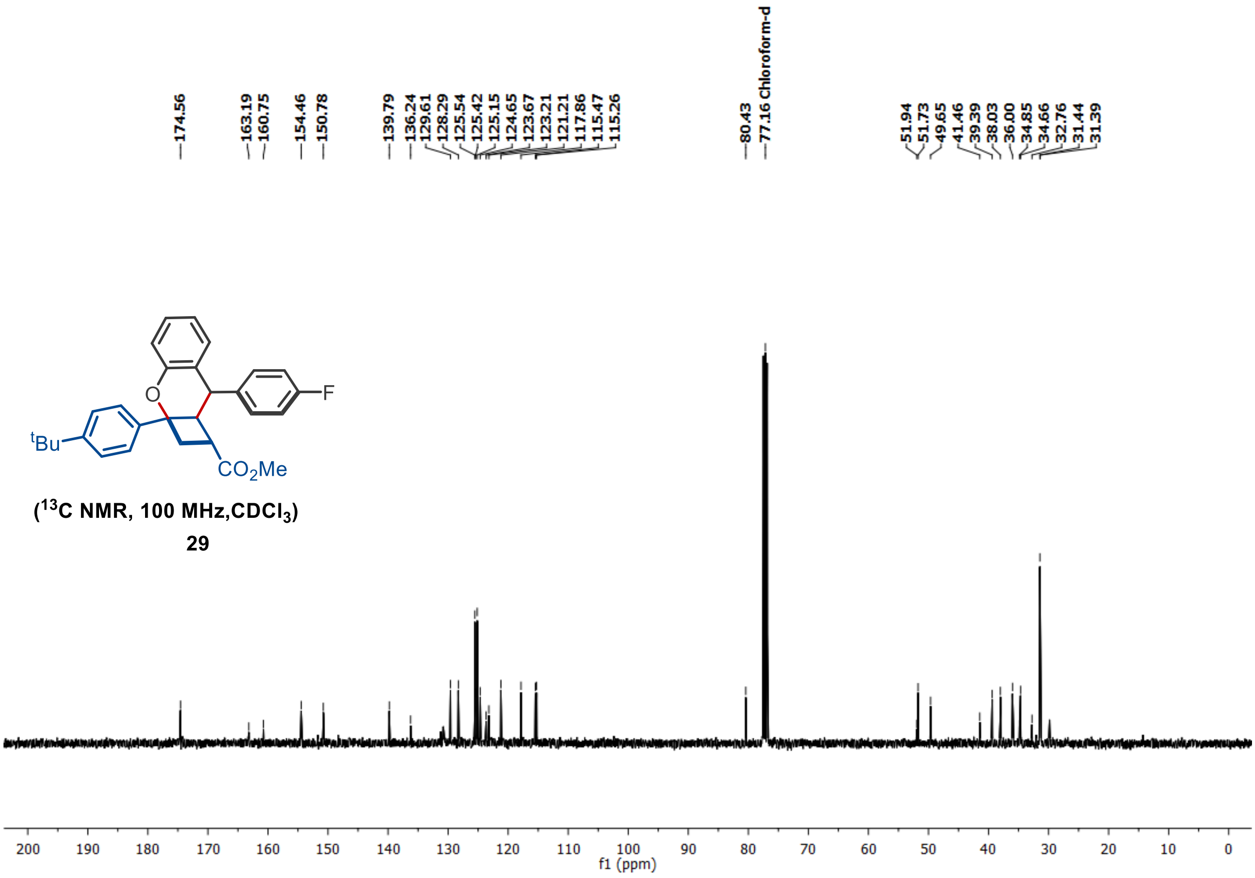

(^{13}C NMR, 100 MHz,$CDCl_3$)

29

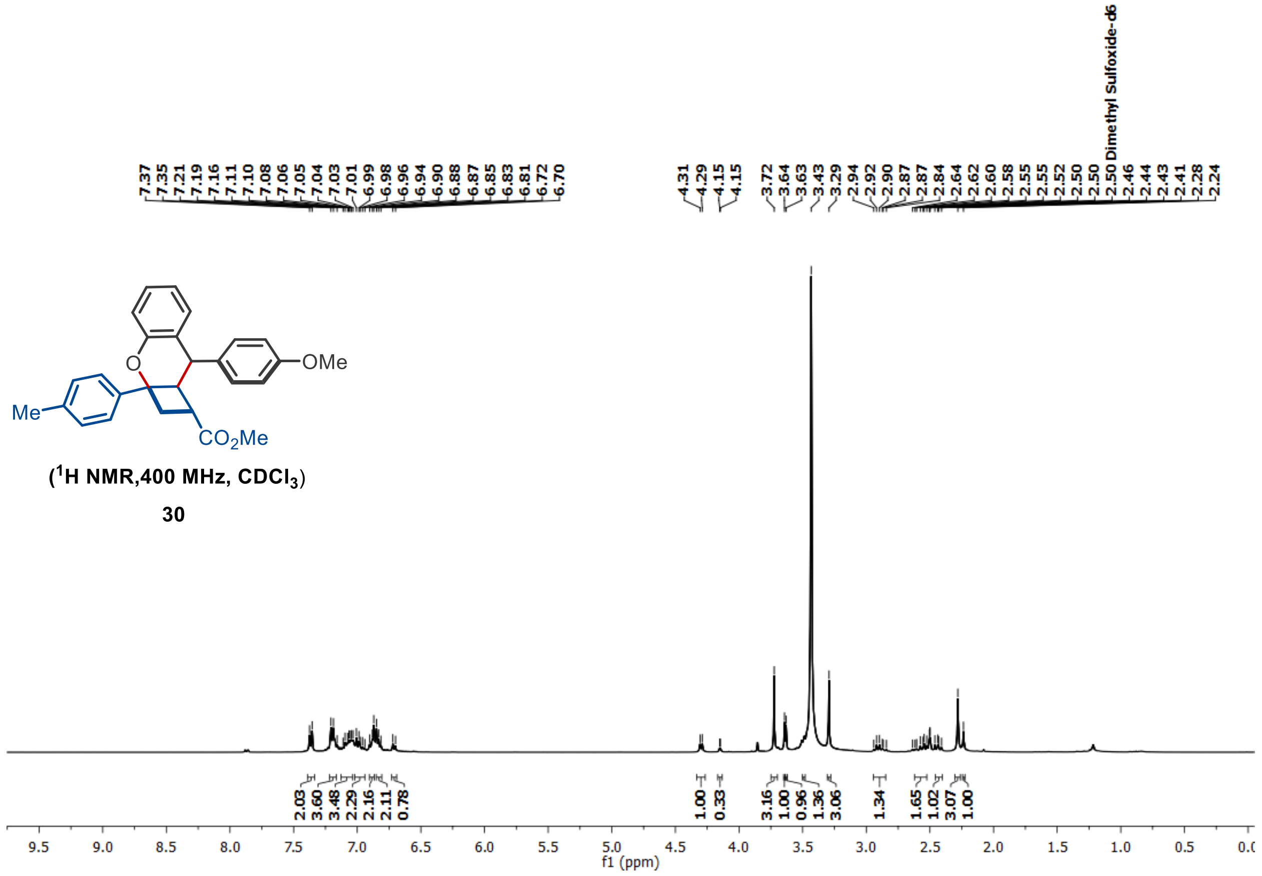
7.37 7.35 7.21 7.19 7.16 7.11 7.10 7.08 7.06 7.05 7.04 7.03 7.01 6.99 6.98 6.96 6.94 6.90 6.88 6.87 6.85 6.83 6.81 6.72 6.70
4.31 4.29 4.15 4.15
3.72 3.64 3.63 3.43 3.29 2.94 2.92 2.90 2.87 2.87 2.84 2.64 2.62 2.60 2.58 2.55 2.55 2.52 2.50 2.50 2.50 Dimethyl Sulfoxide-d6 2.46 2.44 2.43 2.41 2.28 2.24
OMe
O
Me
CO_2Me
(^{1}H NMR,400 MHz, $CDCl_3$)
30
2.03 3.60 3.48 2.29 2.16 2.11 0.78
1.00 0.33
3.16 1.00 0.96 1.36 3.06
1.34
1.65 1.02 3.07 1.00
f1 (ppm)

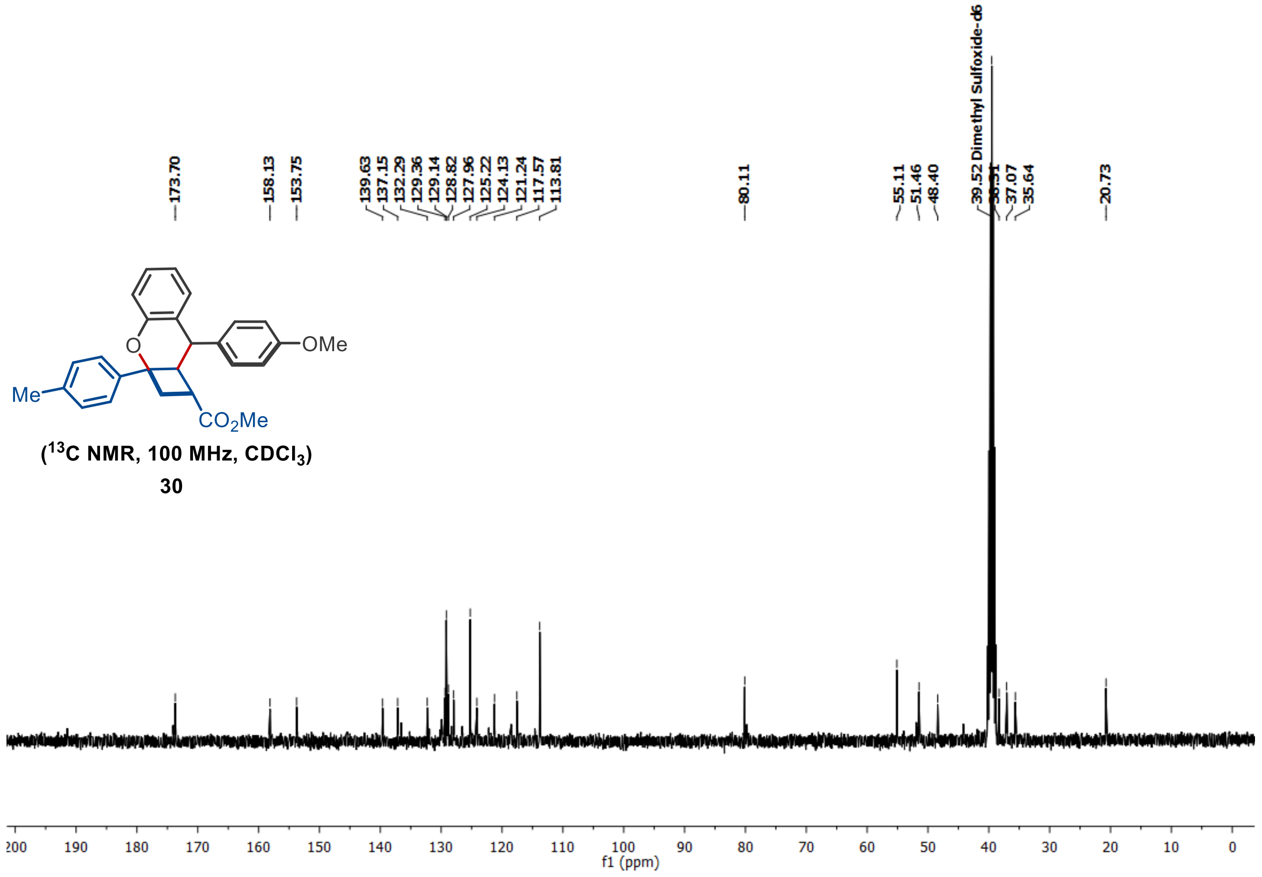
173.70
158.13 153.75
139.63 137.15 132.29 129.36 129.14 128.82 127.96 125.22 124.13 121.24 117.57 113.81
80.11
55.11 51.46 48.40
39.52 Dimethyl Sulfoxide-d6
37.07 35.64
20.73
OMe
O
Me
CO_2Me
(^{13}C NMR, 100 MHz, $CDCl_3$)
30
f1 (ppm)

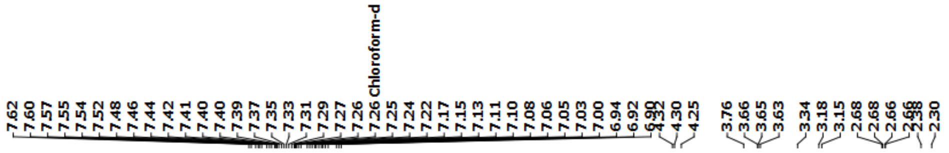

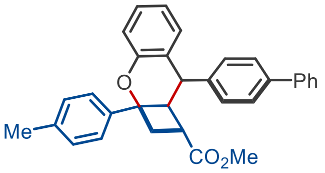

(^{1}H NMR,400 MHz, $CDCl_3$)

31

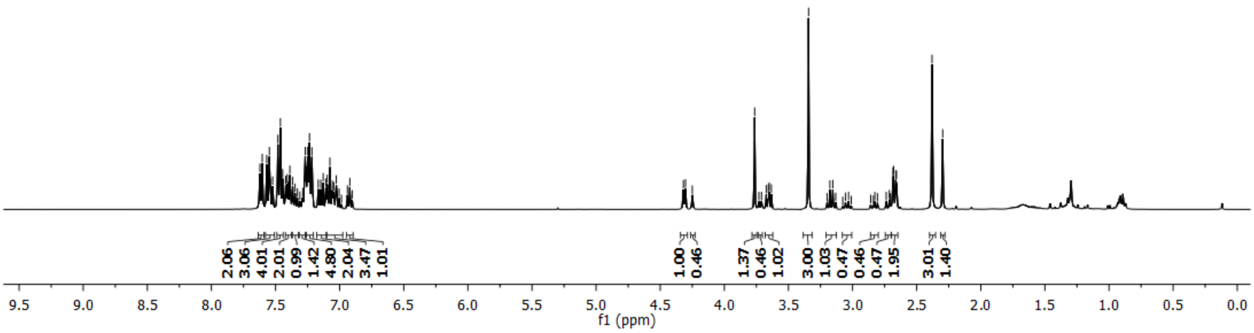

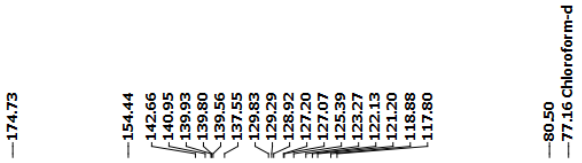

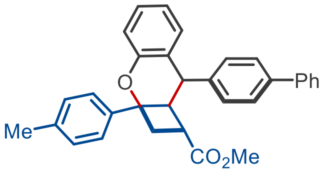

(^{13}C NMR, 100 MHz, $CDCl_3$)

31

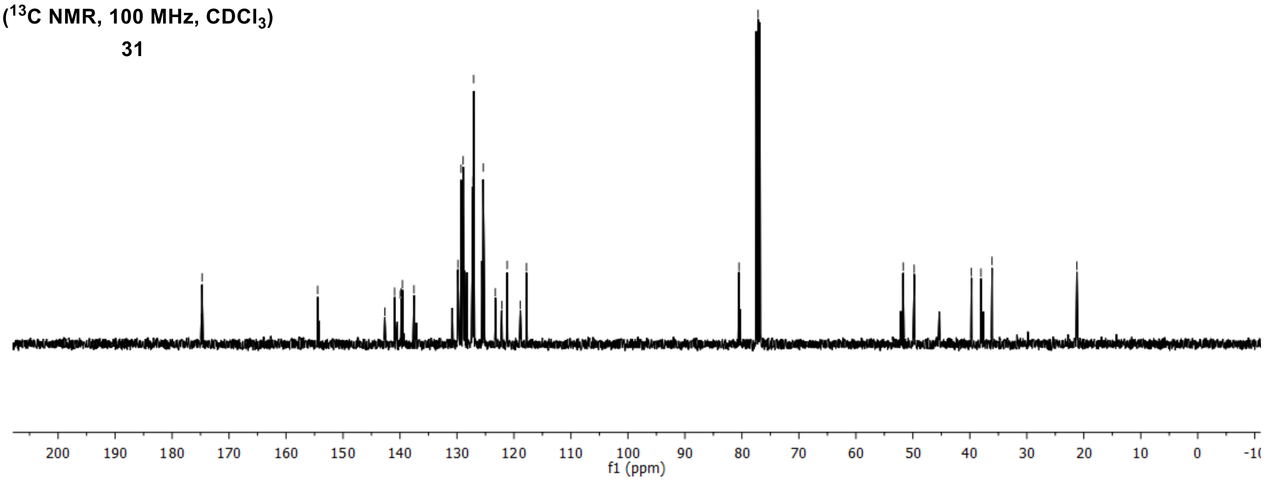

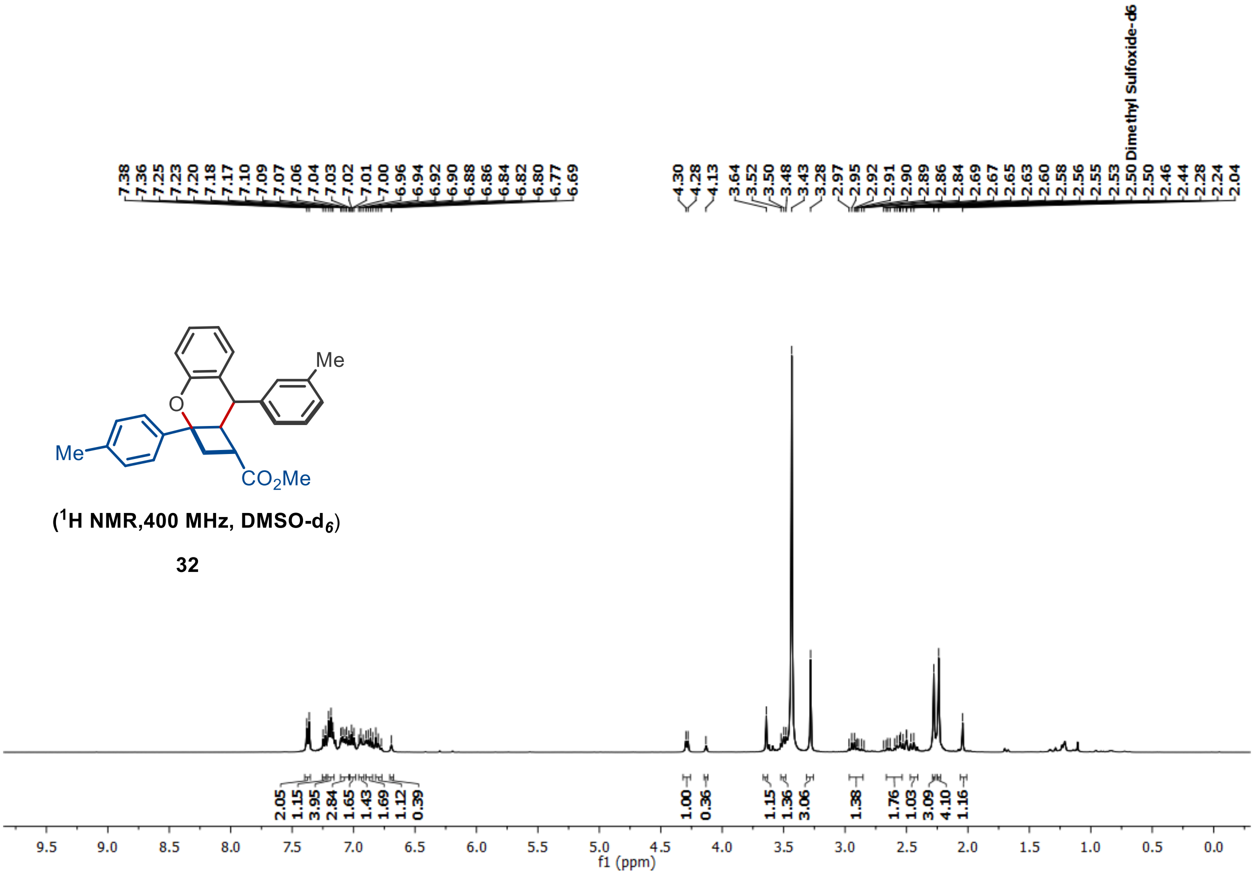

(^{1}H NMR,400 MHz, DMSO-d$_6$)

32

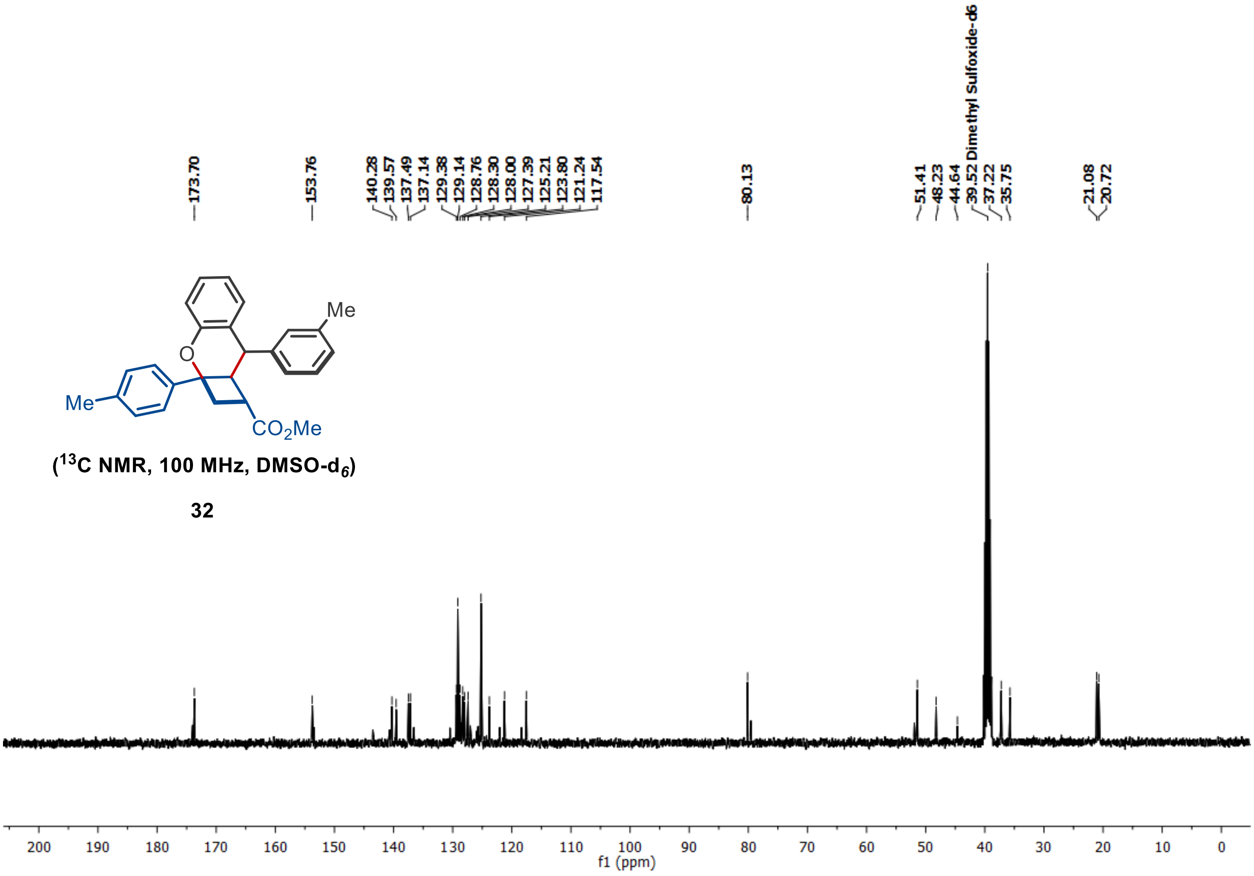

(^{13}C NMR, 100 MHz, DMSO-d$_6$)

32

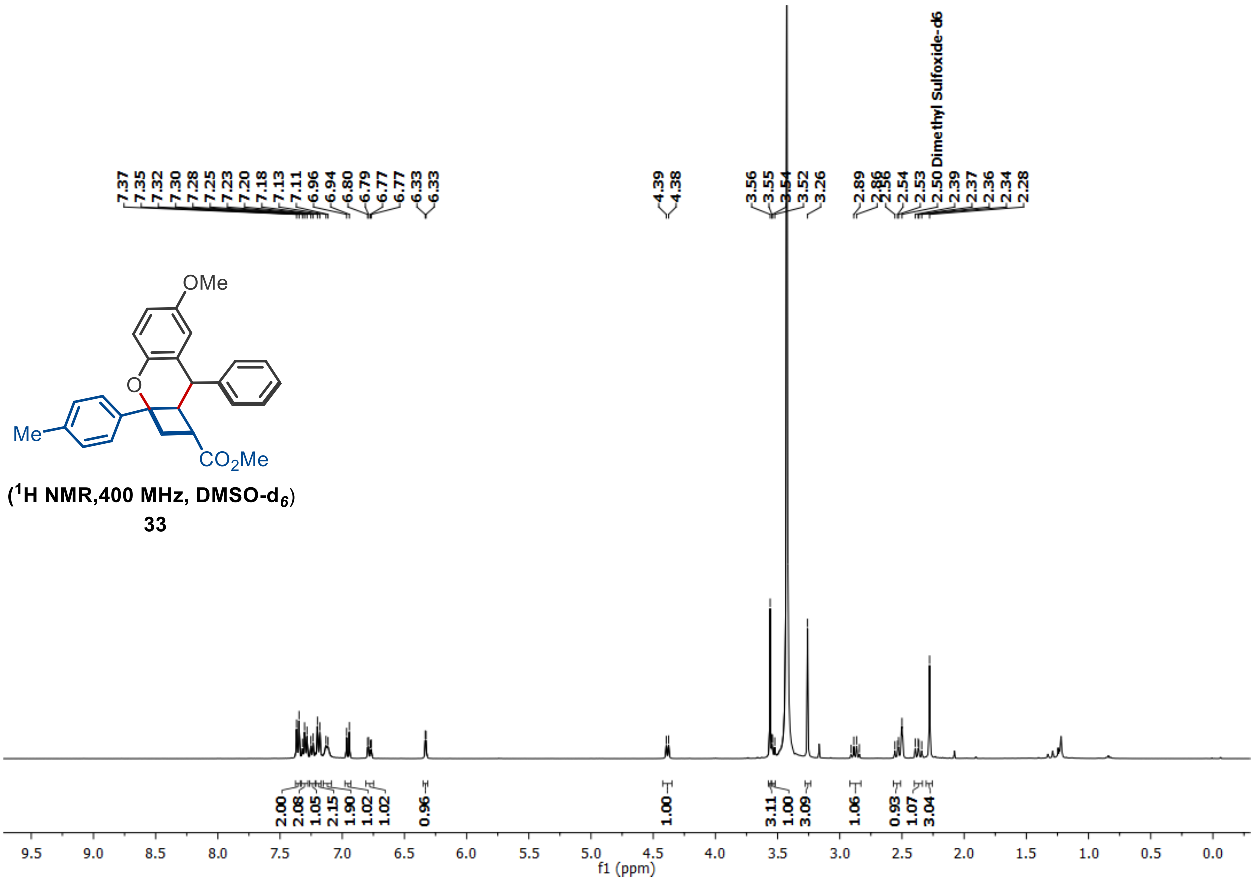

(^{1}H NMR,400 MHz, DMSO-d_6)

33

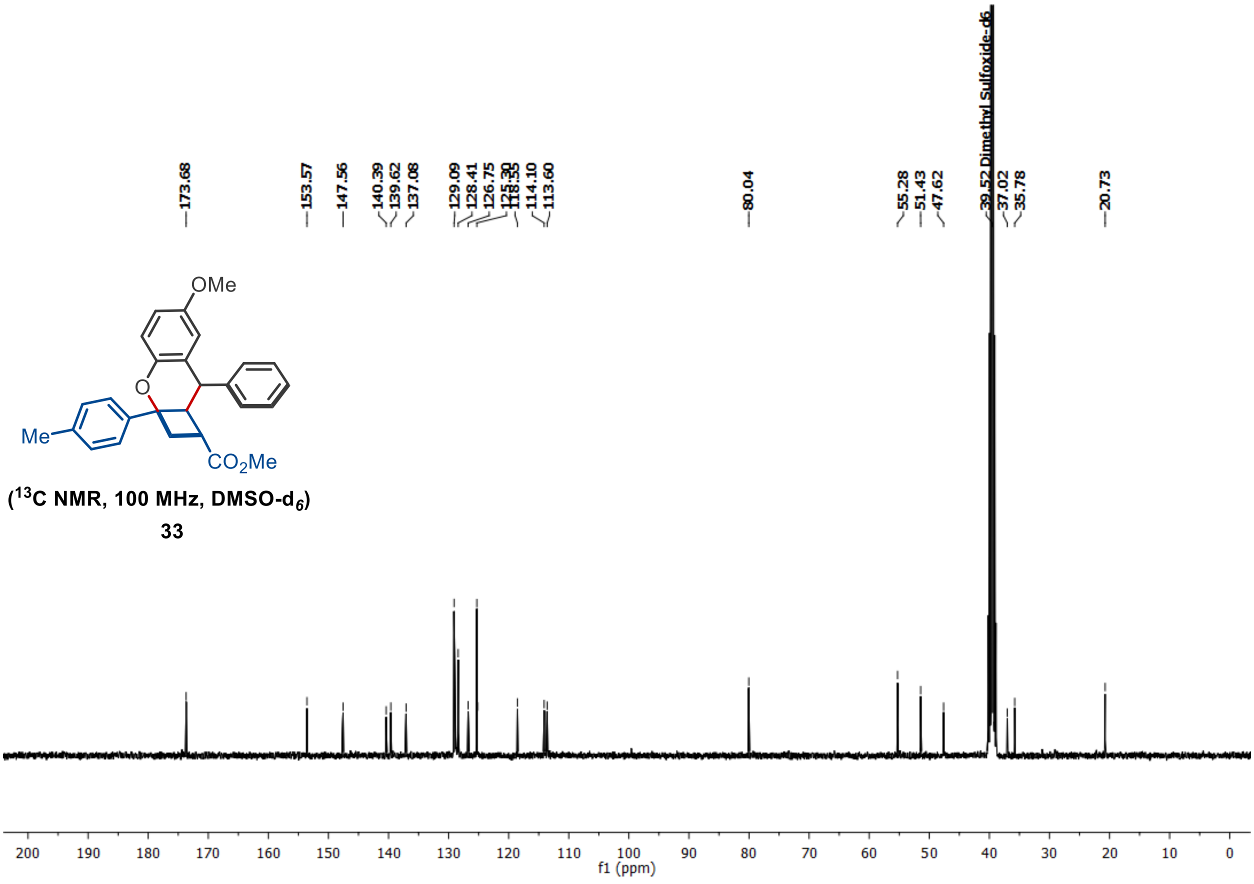

(^{13}C NMR, 100 MHz, DMSO-d_6)

33

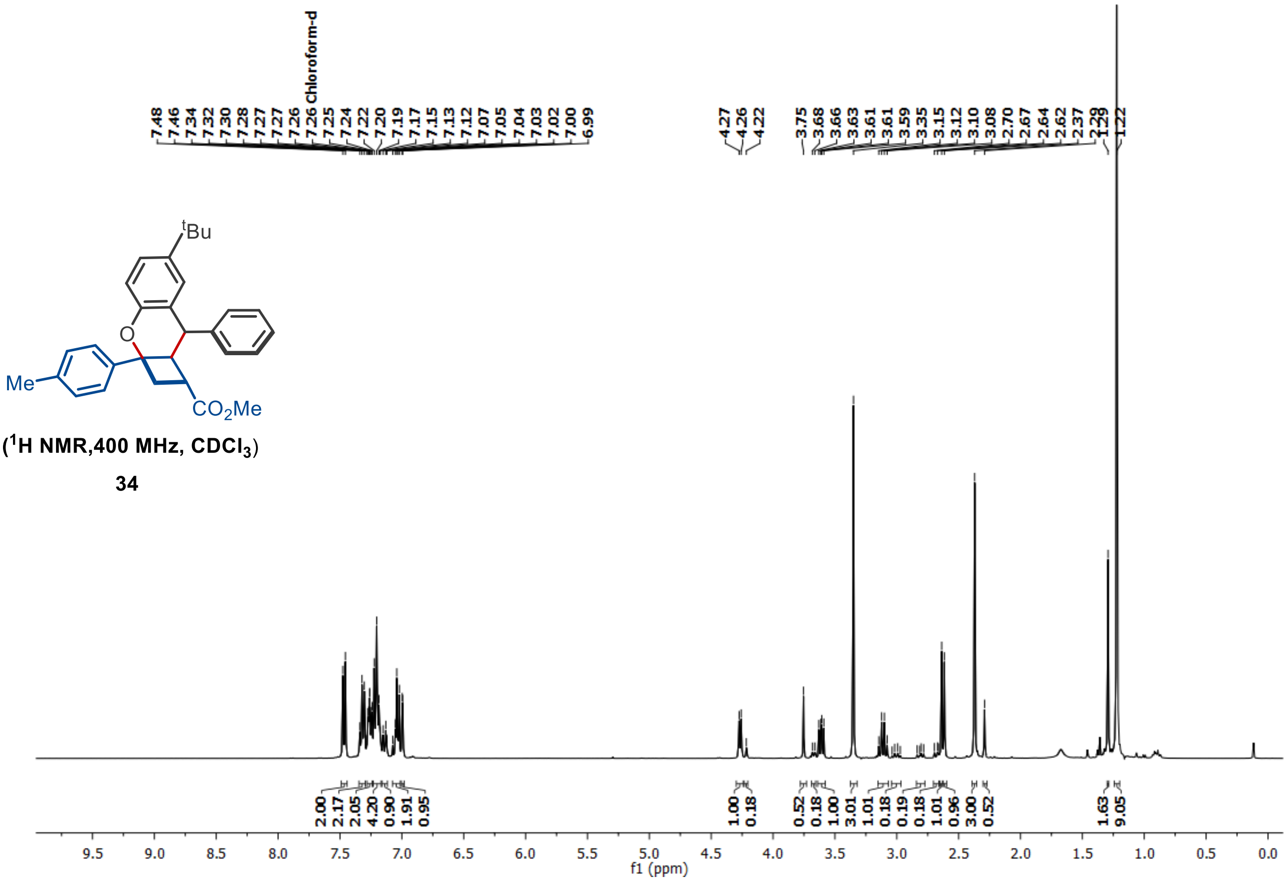

(**^{1}H NMR,400 MHz, $CDCl_3$**)

34

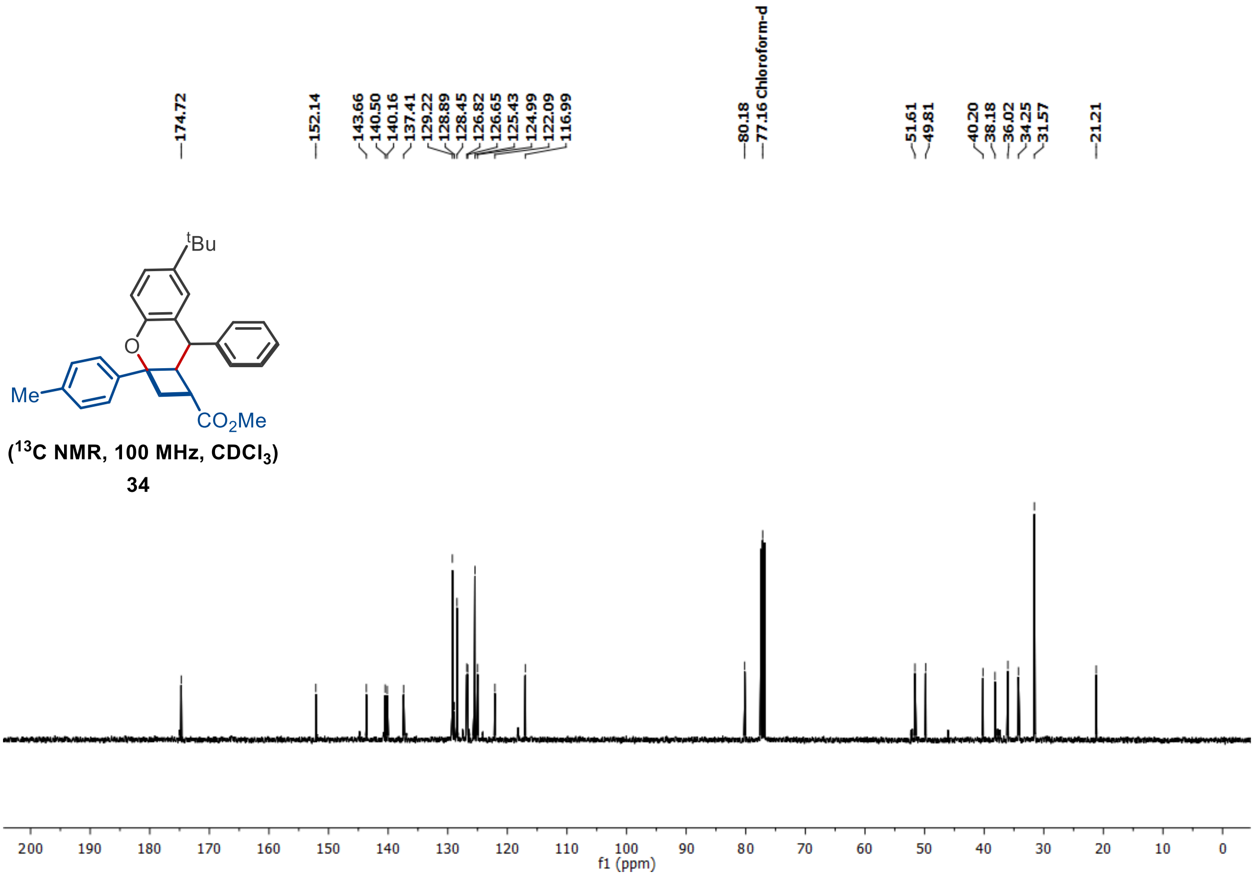

(**^{13}C NMR, 100 MHz, $CDCl_3$**)

34

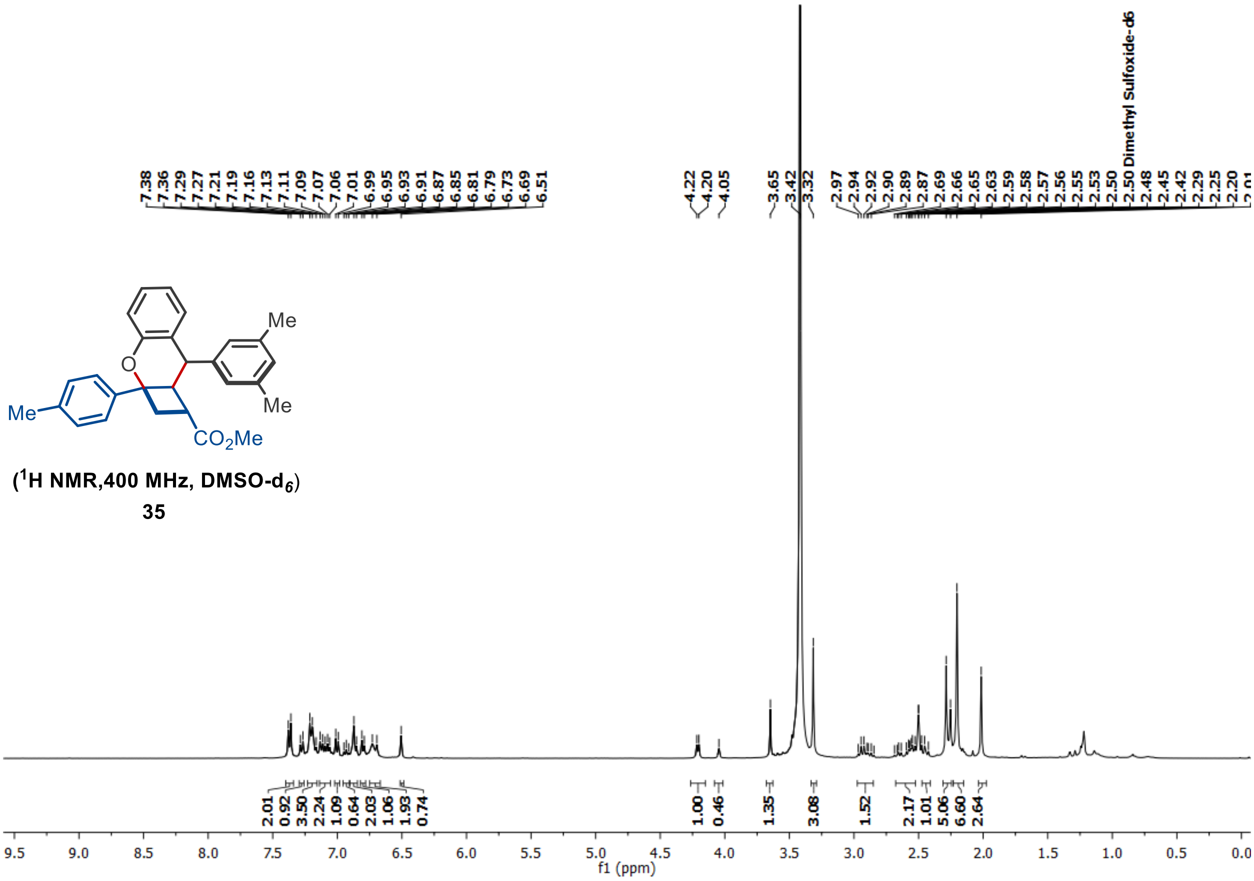

(^{1}H NMR,400 MHz, DMSO-d_6)

35

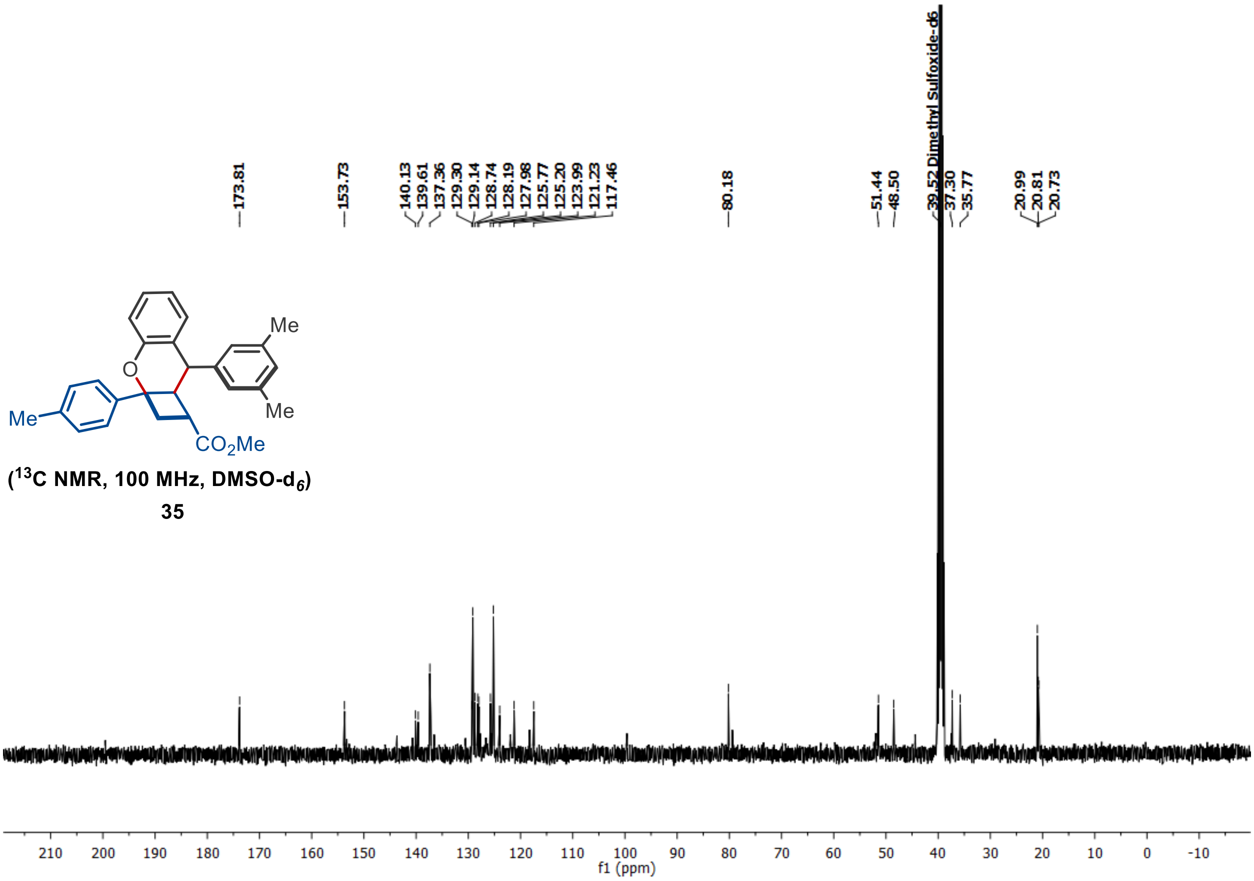

(^{13}C NMR, 100 MHz, DMSO-d_6)

35

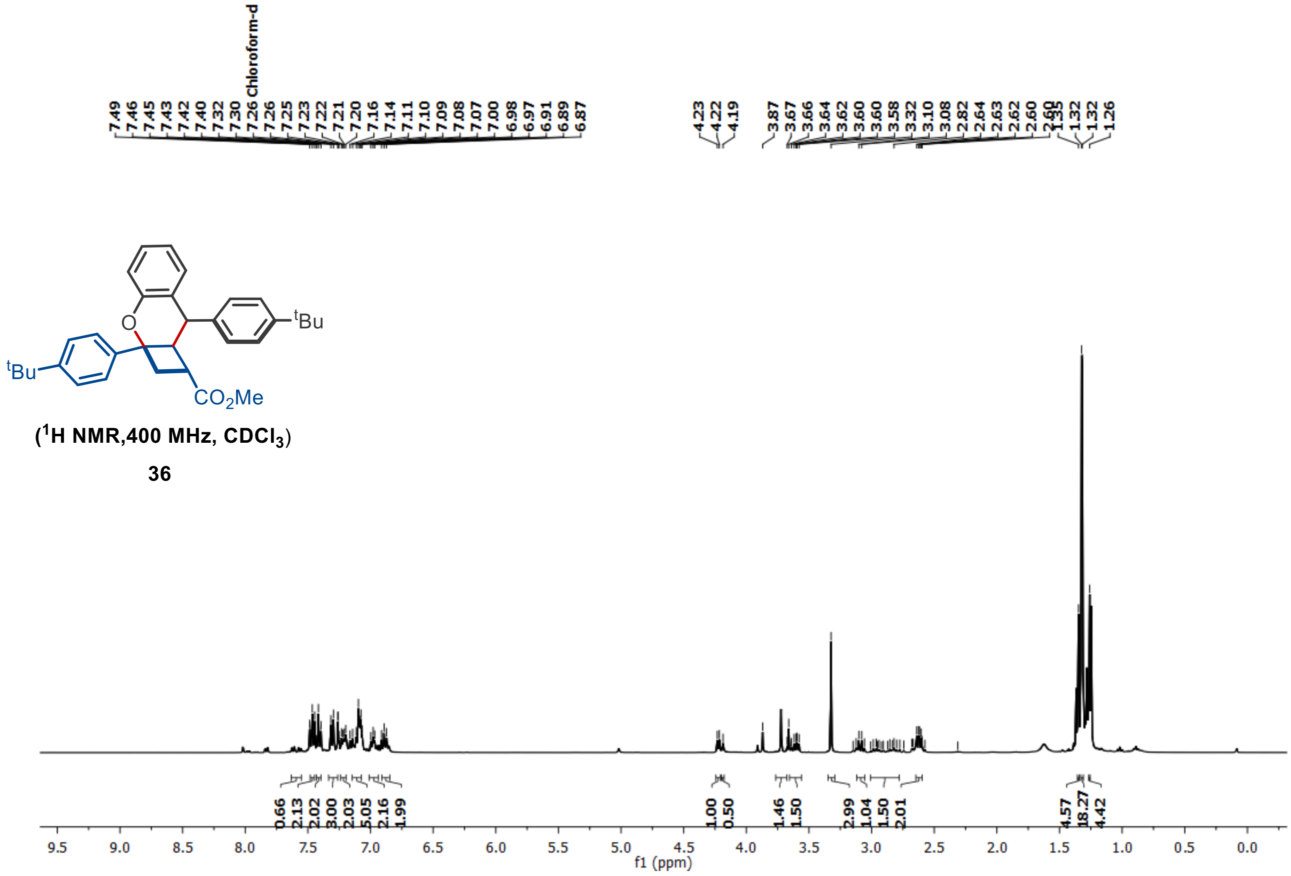

(^{1}H NMR,400 MHz, $CDCl_3$)

36

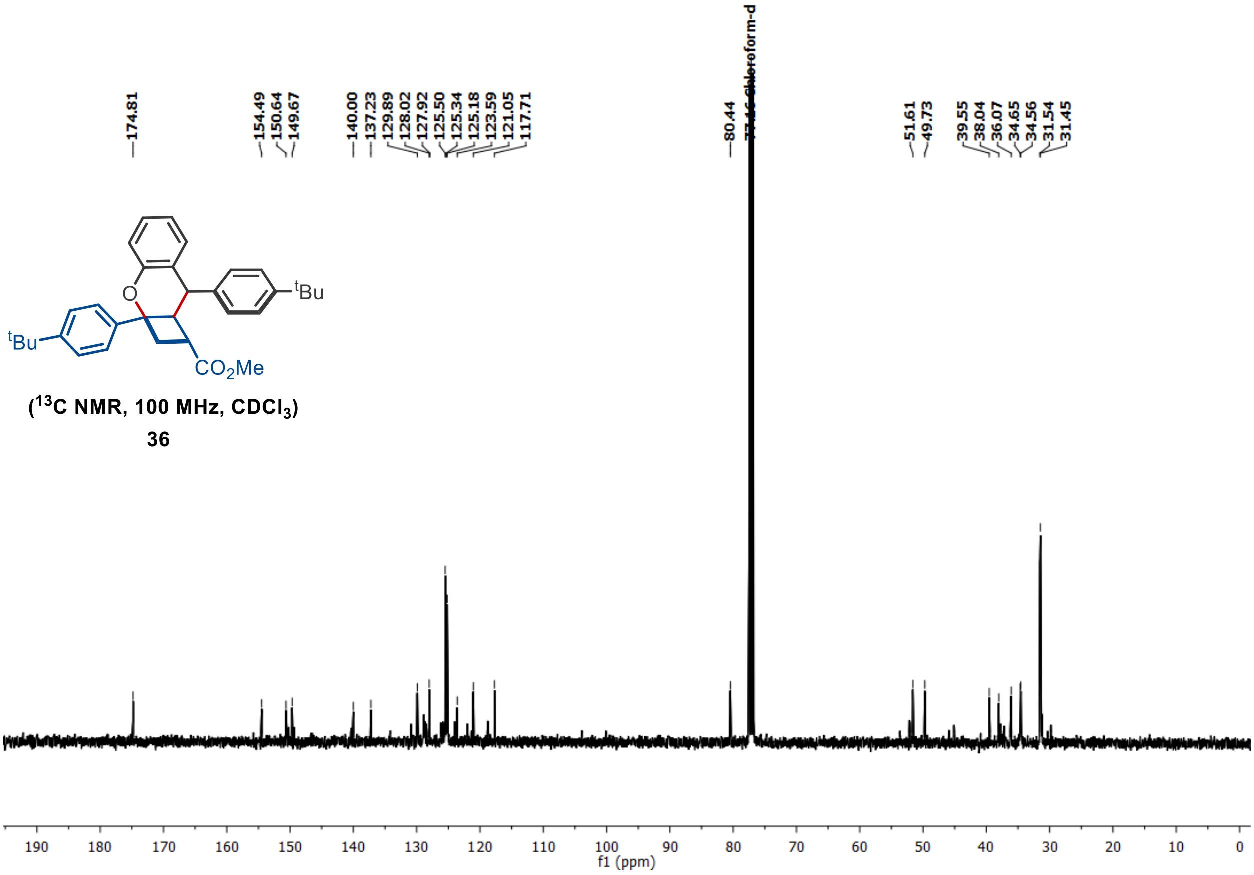

(^{13}C NMR, 100 MHz, $CDCl_3$)

36

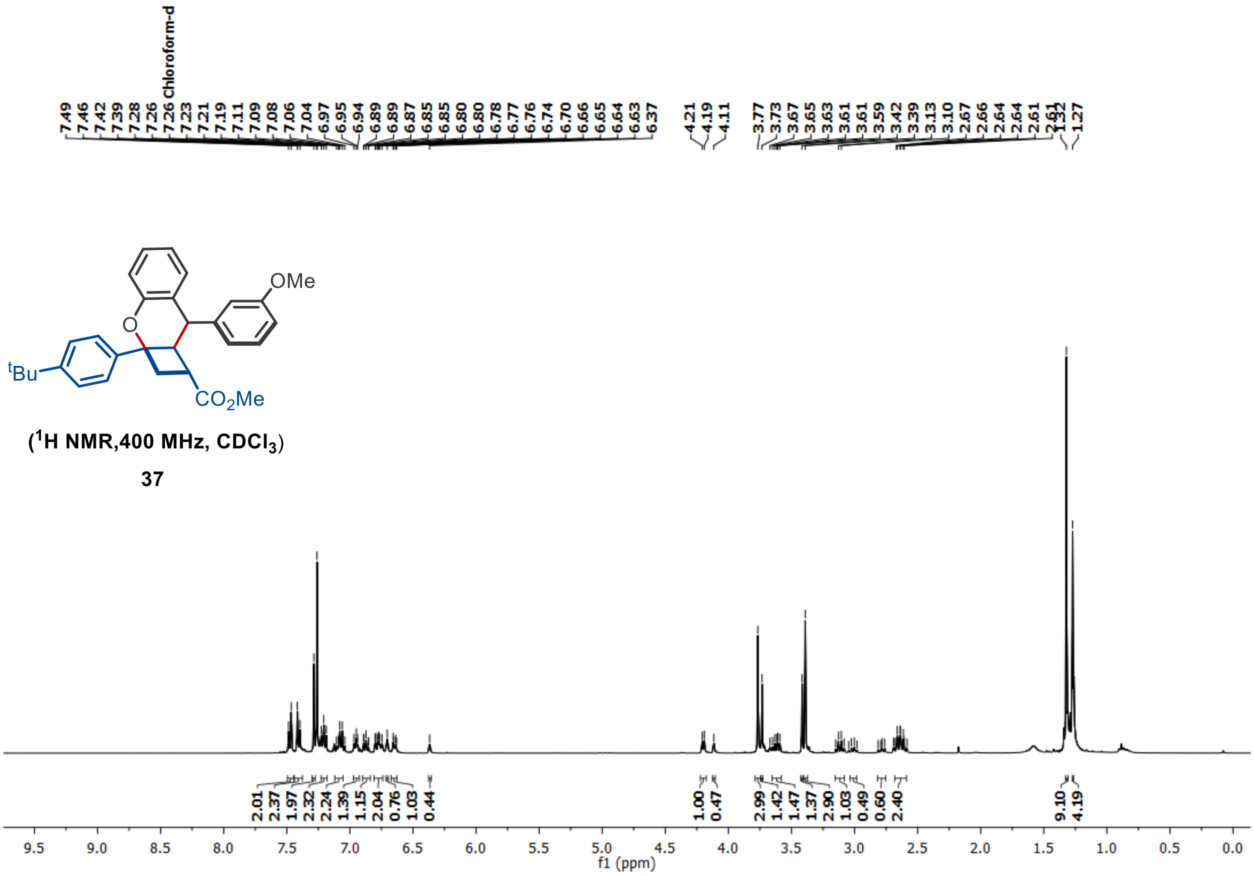

(^{1}H NMR,400 MHz, $CDCl_3$)

37

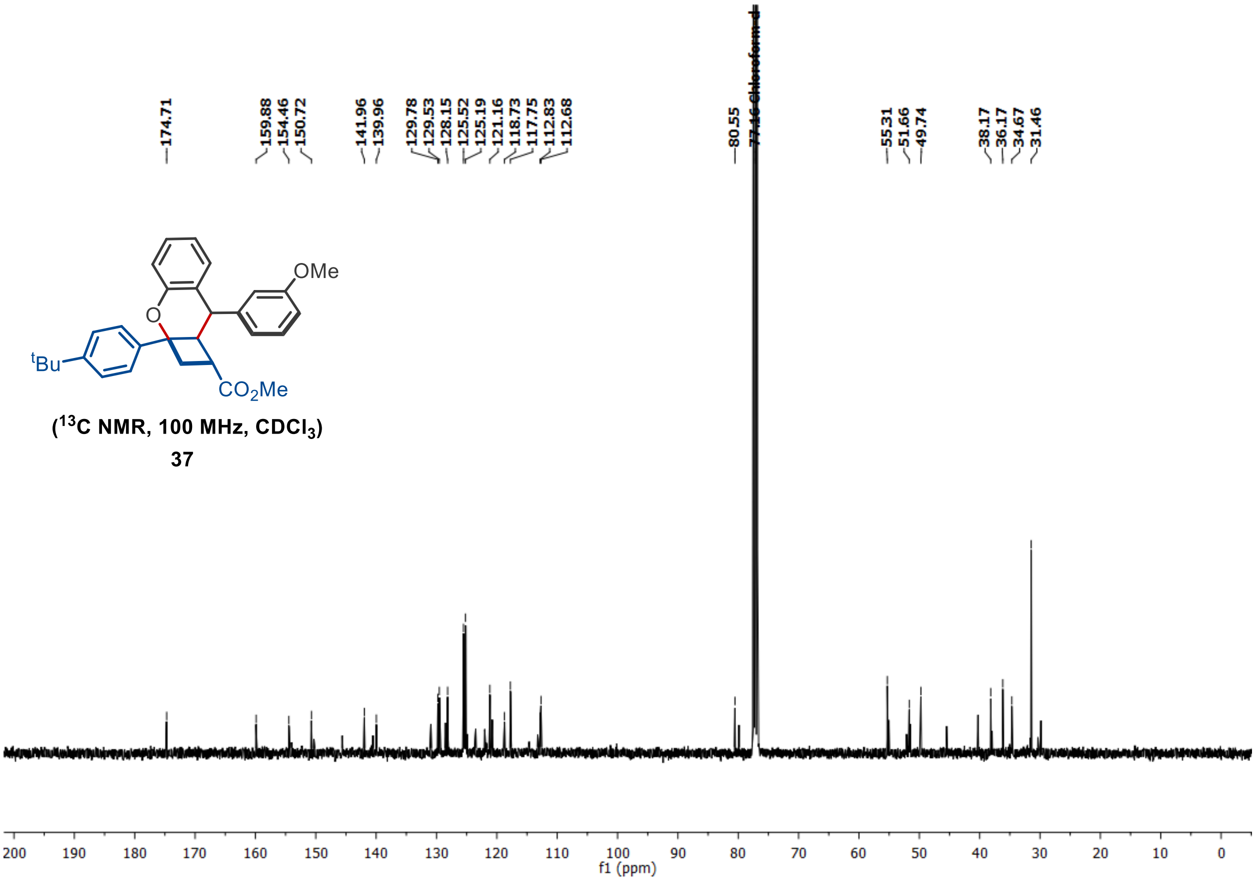

(^{13}C NMR, 100 MHz, $CDCl_3$)

37

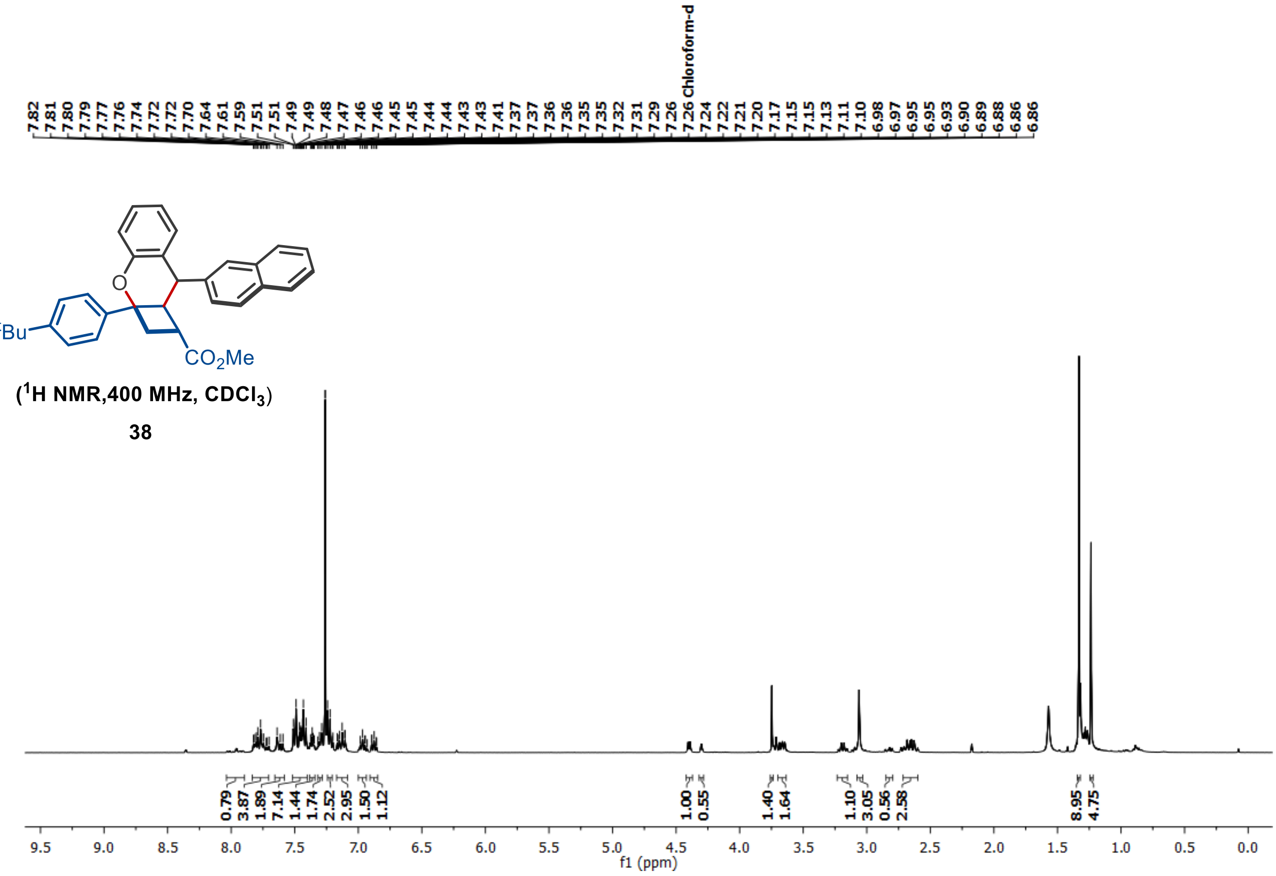

(^{1}H NMR,400 MHz, $CDCl_3$)

38

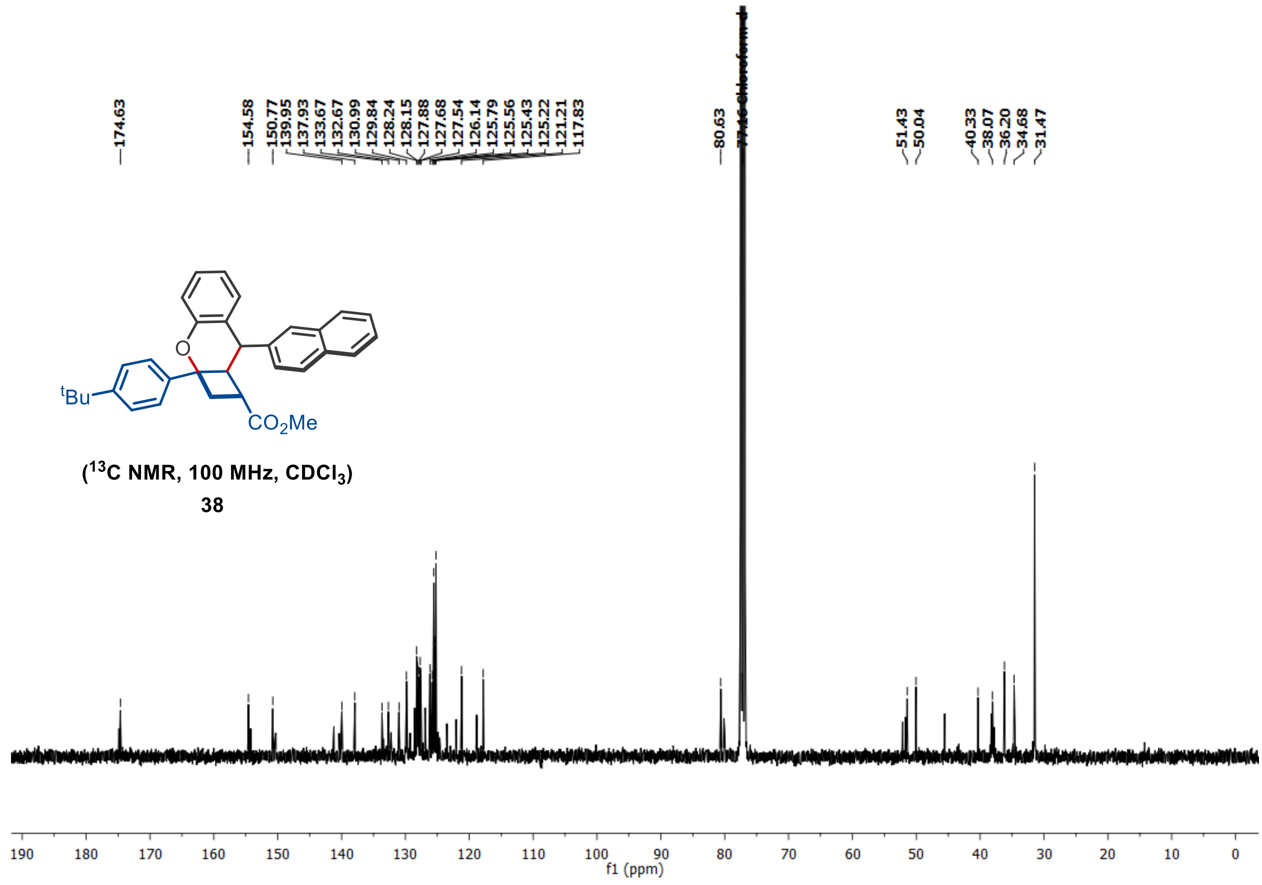

(^{13}C NMR, 100 MHz, $CDCl_3$)

38

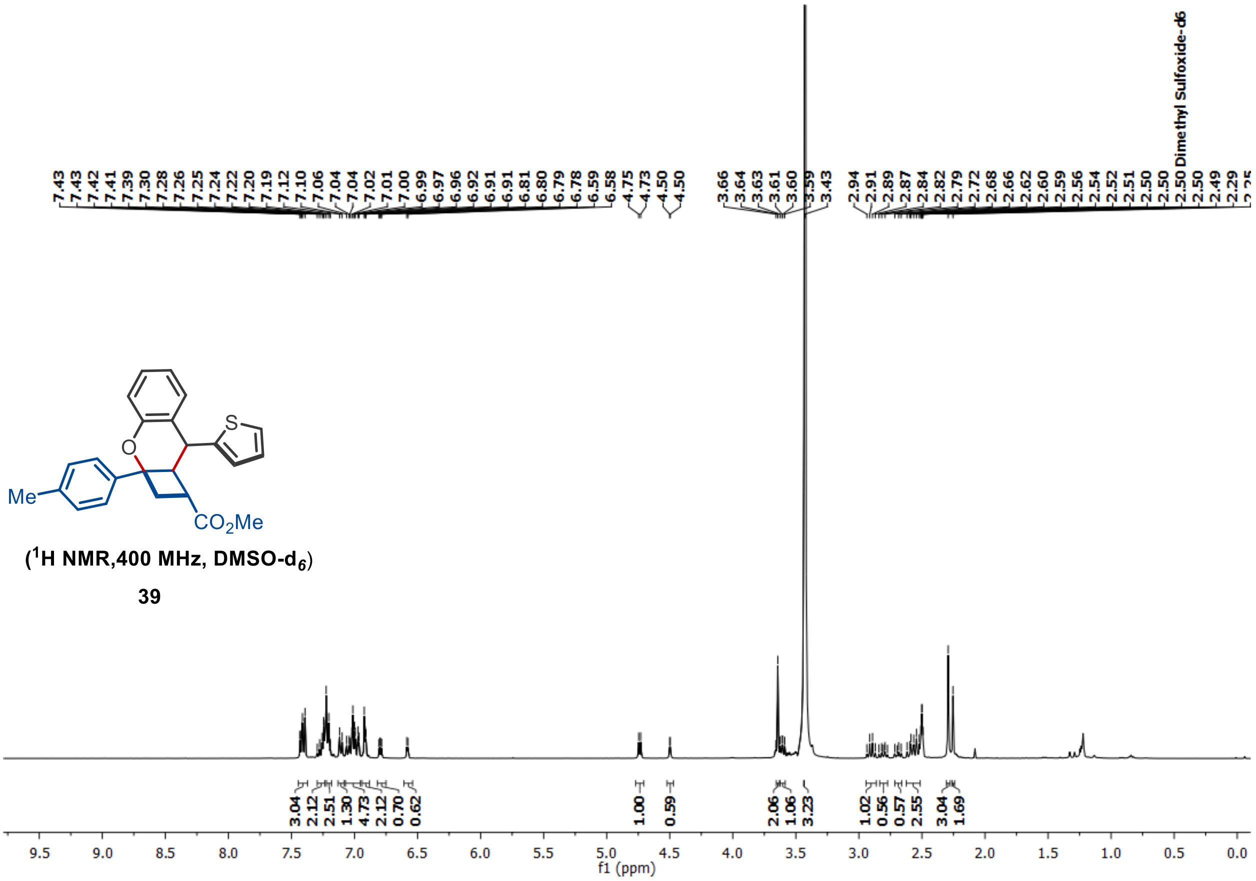

(^{1}H NMR,400 MHz, DMSO-d_6)

39

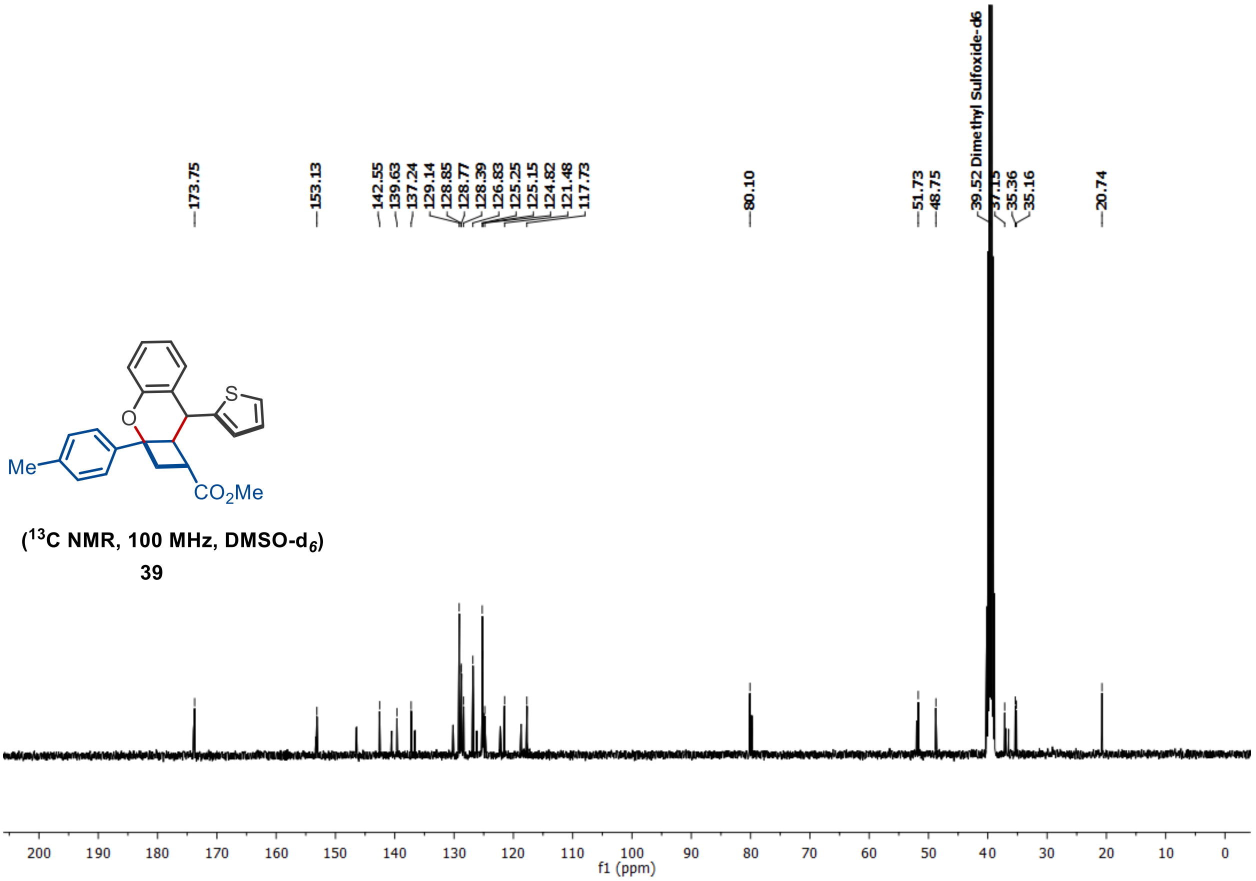

(^{13}C NMR, 100 MHz, DMSO-d_6)

39

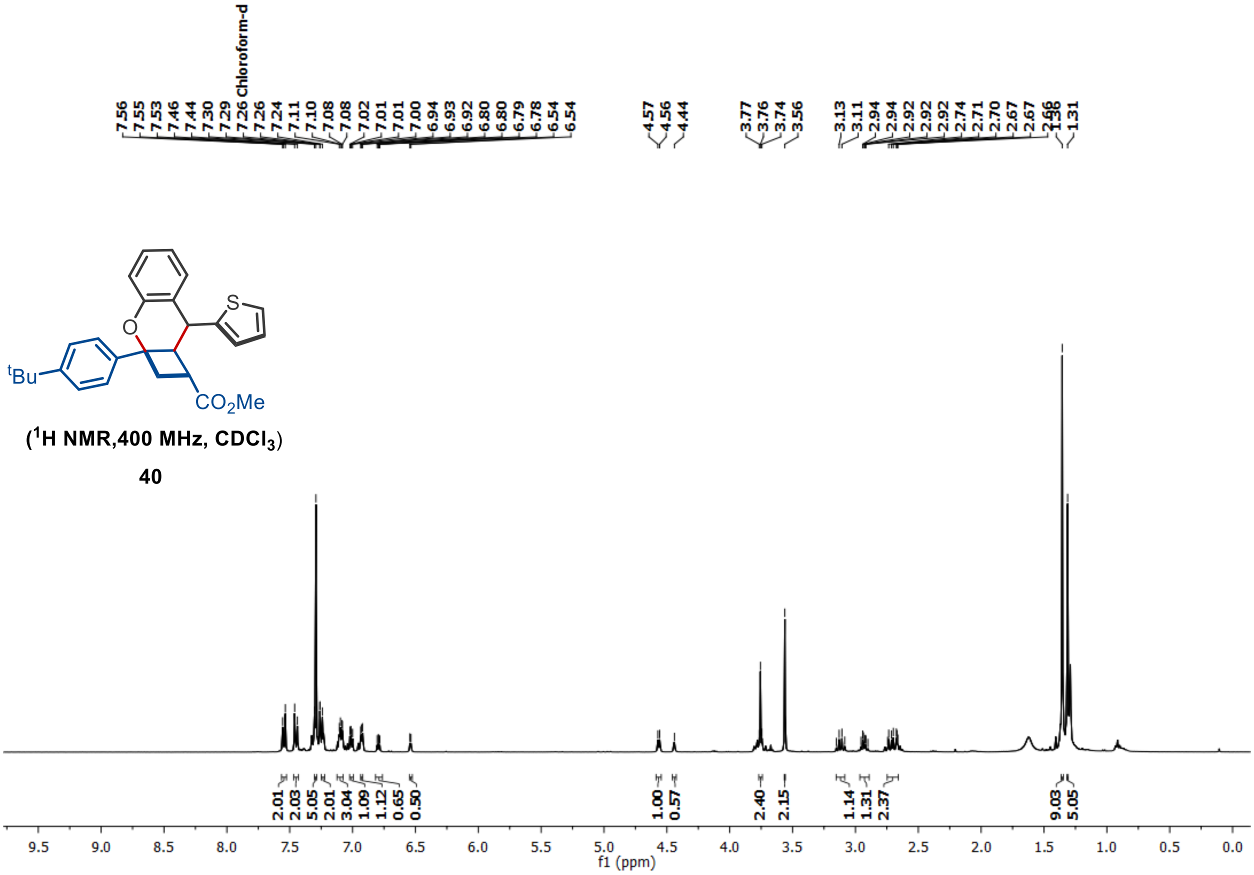
7.56 7.55 7.53 7.46 7.44 7.30 7.29 7.26 Chloroform-d 7.26 7.24 7.11 7.10 7.08 7.08 7.02 7.01 7.01 7.00 6.94 6.93 6.92 6.80 6.80 6.79 6.78 6.54 6.54
4.57 4.56 4.44
3.77 3.76 3.74 3.56
3.13 3.11 2.94 2.94 2.92 2.92 2.92 2.74 2.71 2.70 2.67 2.67 2.66 1.36 1.31
tBu
S
O
CO_2Me
(^{1}H NMR,400 MHz, $CDCl_3$)
40
2.01 2.03 5.05 2.01 3.04 1.09 1.12 0.65 0.50
1.00 0.57
2.40 2.15
1.14 1.31 2.37
9.03 5.05
9.5 9.0 8.5 8.0 7.5 7.0 6.5 6.0 5.5 5.0 4.5 4.0 3.5 3.0 2.5 2.0 1.5 1.0 0.5 0.0
f1 (ppm)

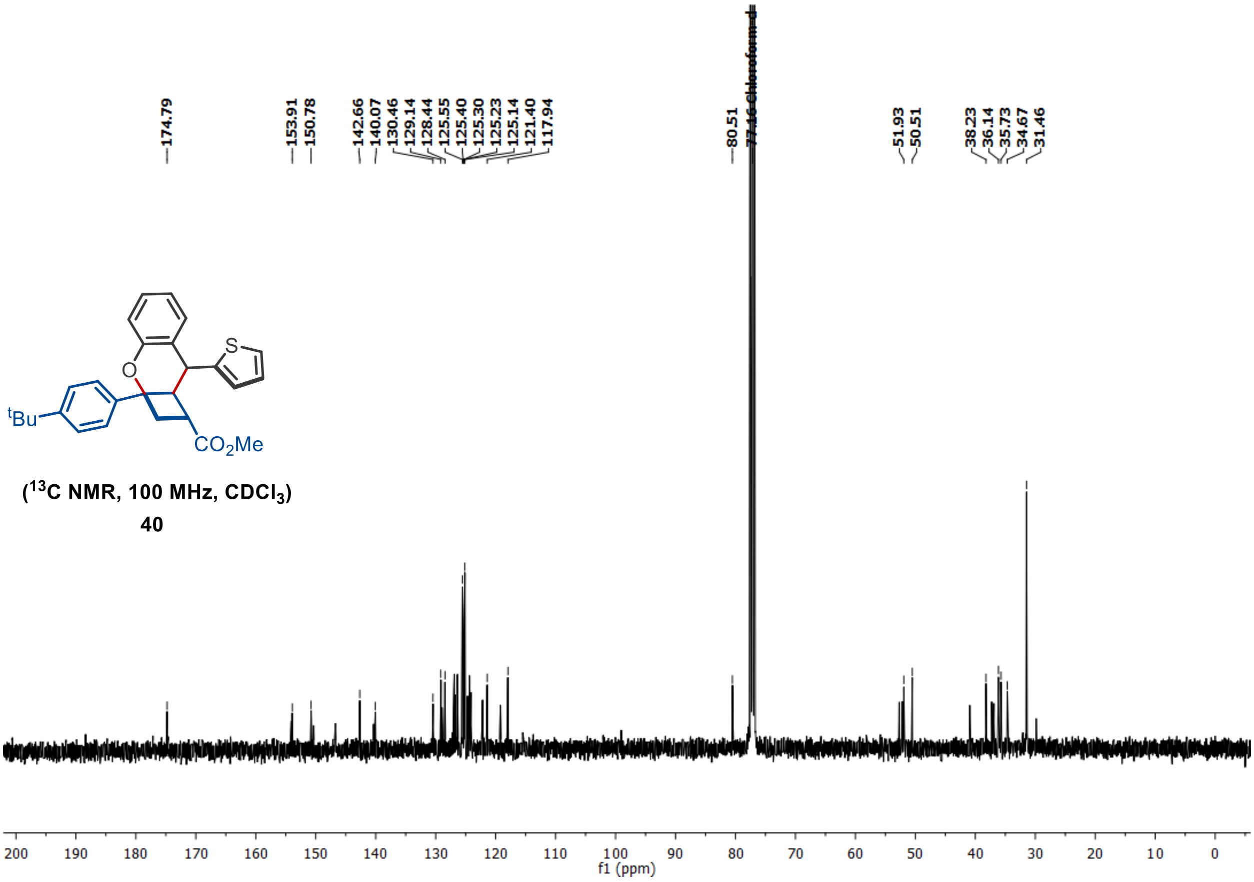
174.79
153.91 150.78
142.66 140.07 130.46 129.14 128.44 125.55 125.40 125.30 125.23 125.14 121.40 117.94
80.51
77.16 Chloroform-d
51.93 50.51
38.23 36.14 35.73 34.67 31.46
tBu
S
O
CO_2Me
(^{13}C NMR, 100 MHz, $CDCl_3$)
40
200 190 180 170 160 150 140 130 120 110 100 90 80 70 60 50 40 30 20 10 0
f1 (ppm)

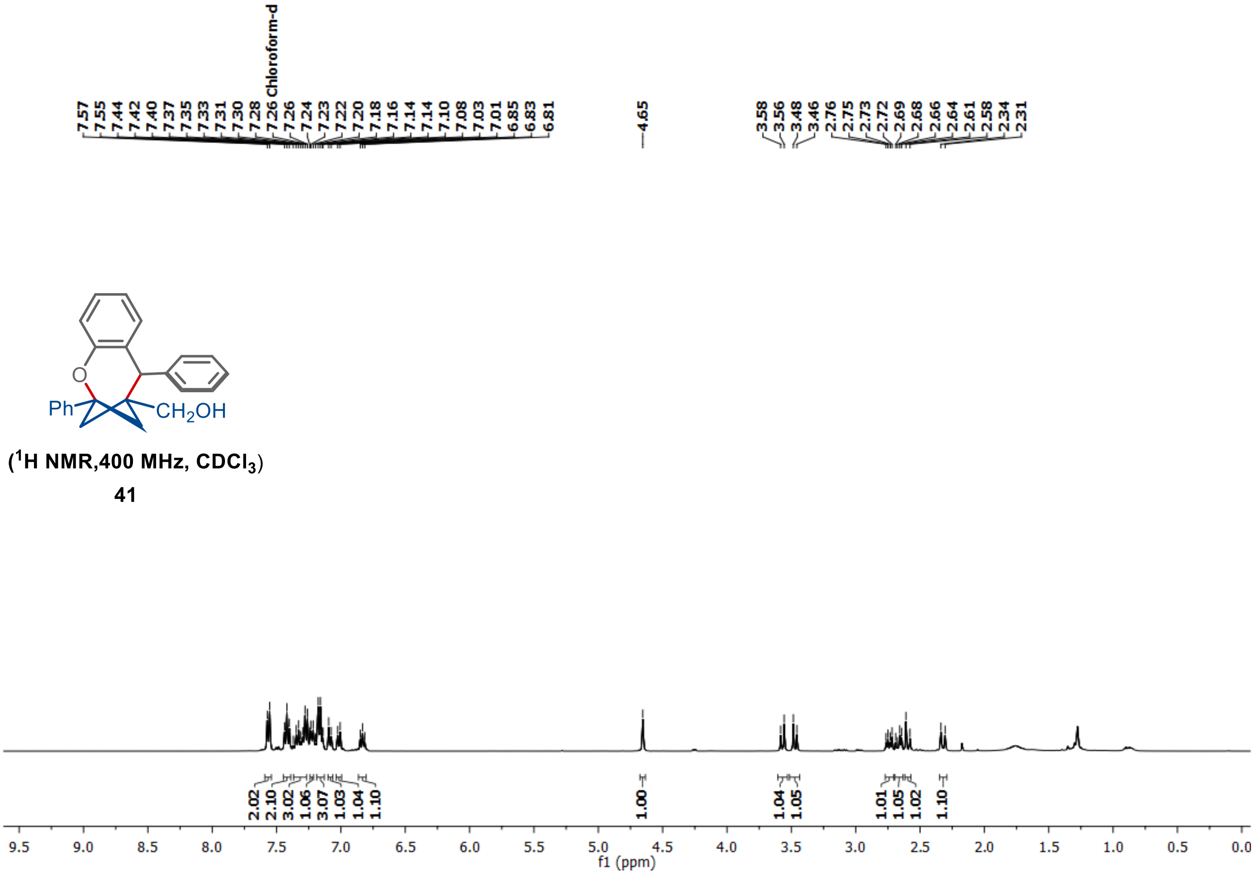

(^{1}H NMR,400 MHz, $CDCl_3$)

41

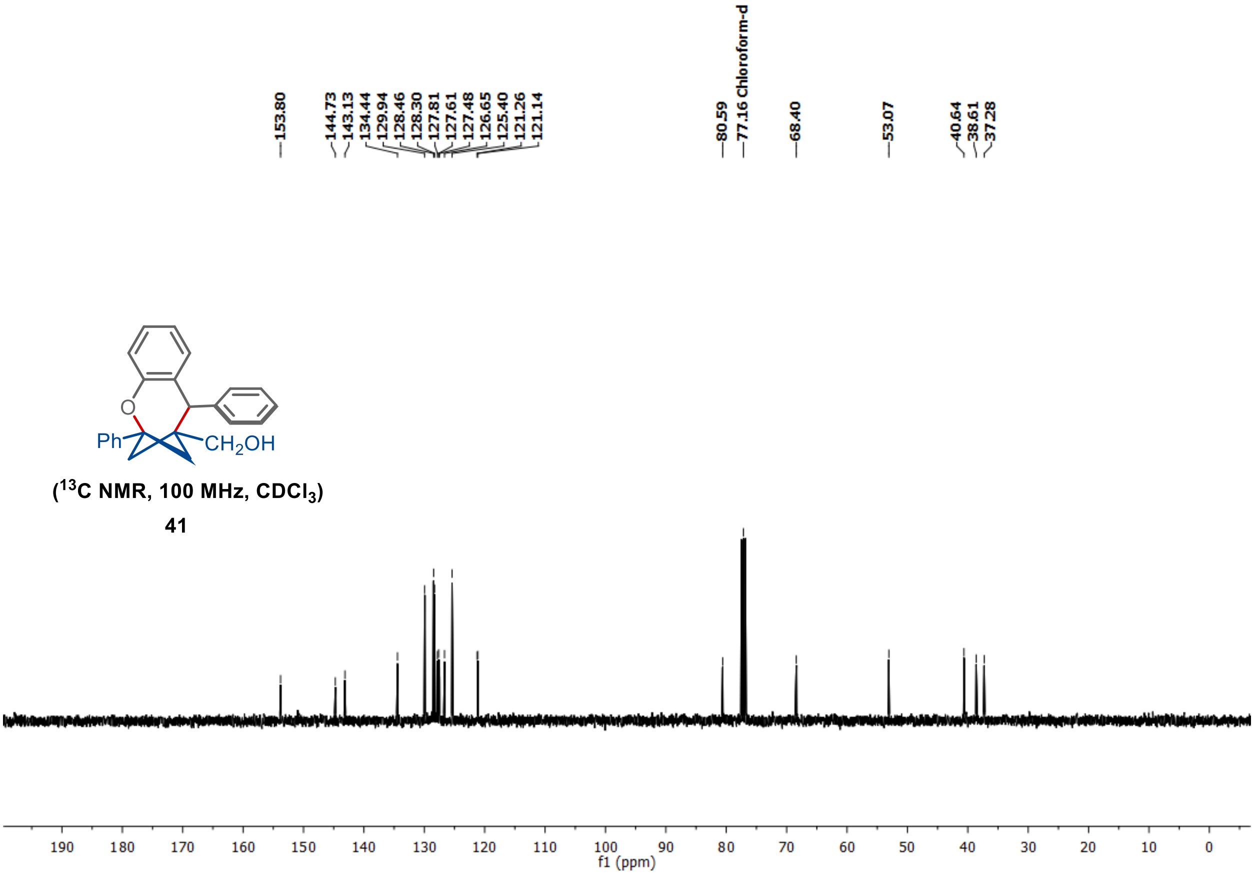

(^{13}C NMR, 100 MHz, $CDCl_3$)

41

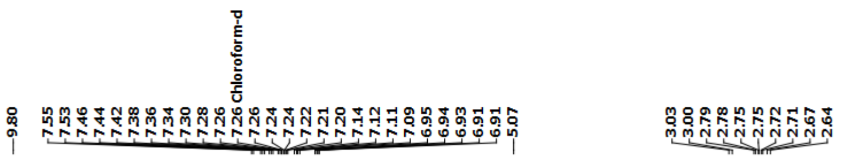

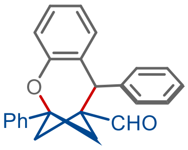

(1H NMR,400 MHz, $CDCl_3$)

42

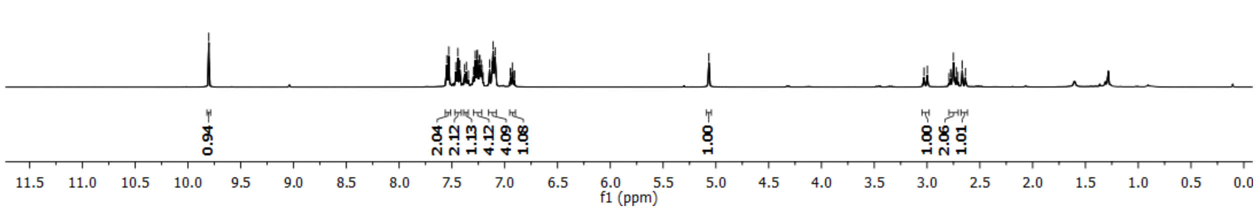

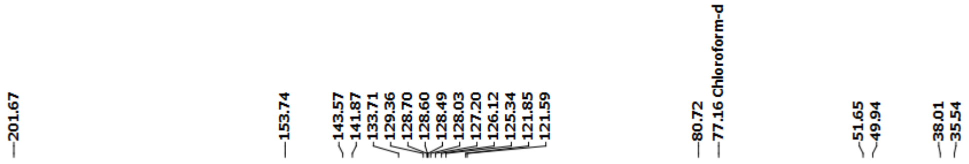

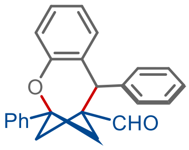

(13C NMR, 100 MHz, $CDCl_3$)

42

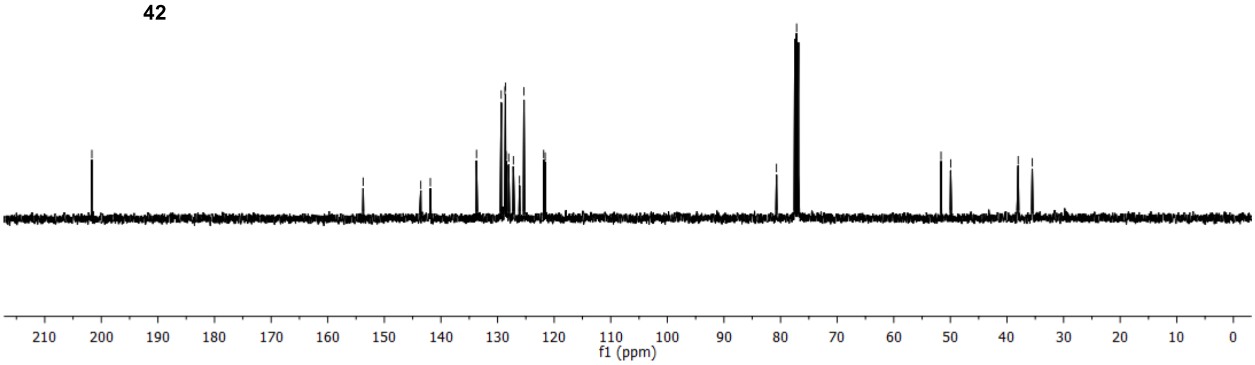

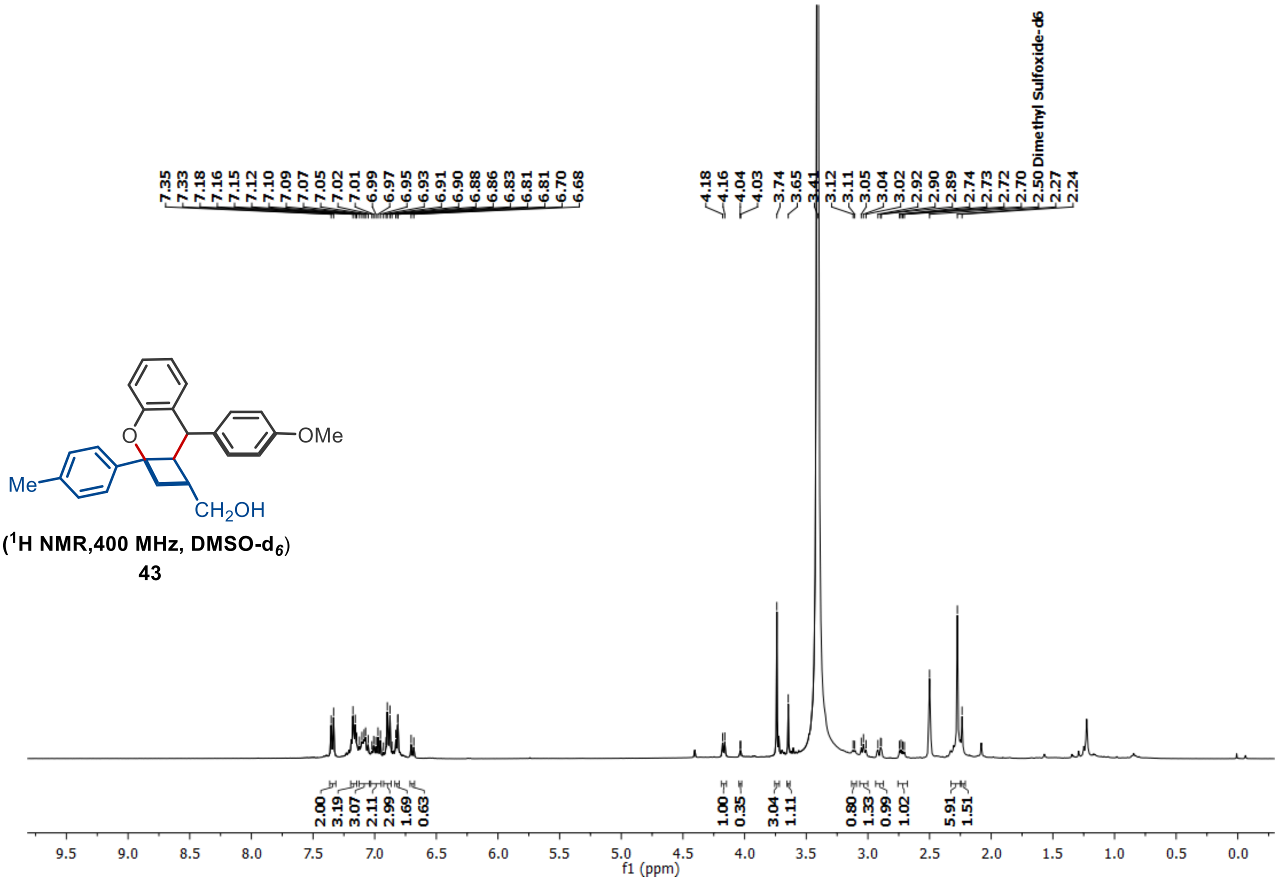

(^{1}H NMR,400 MHz, DMSO-d$_6$)

43

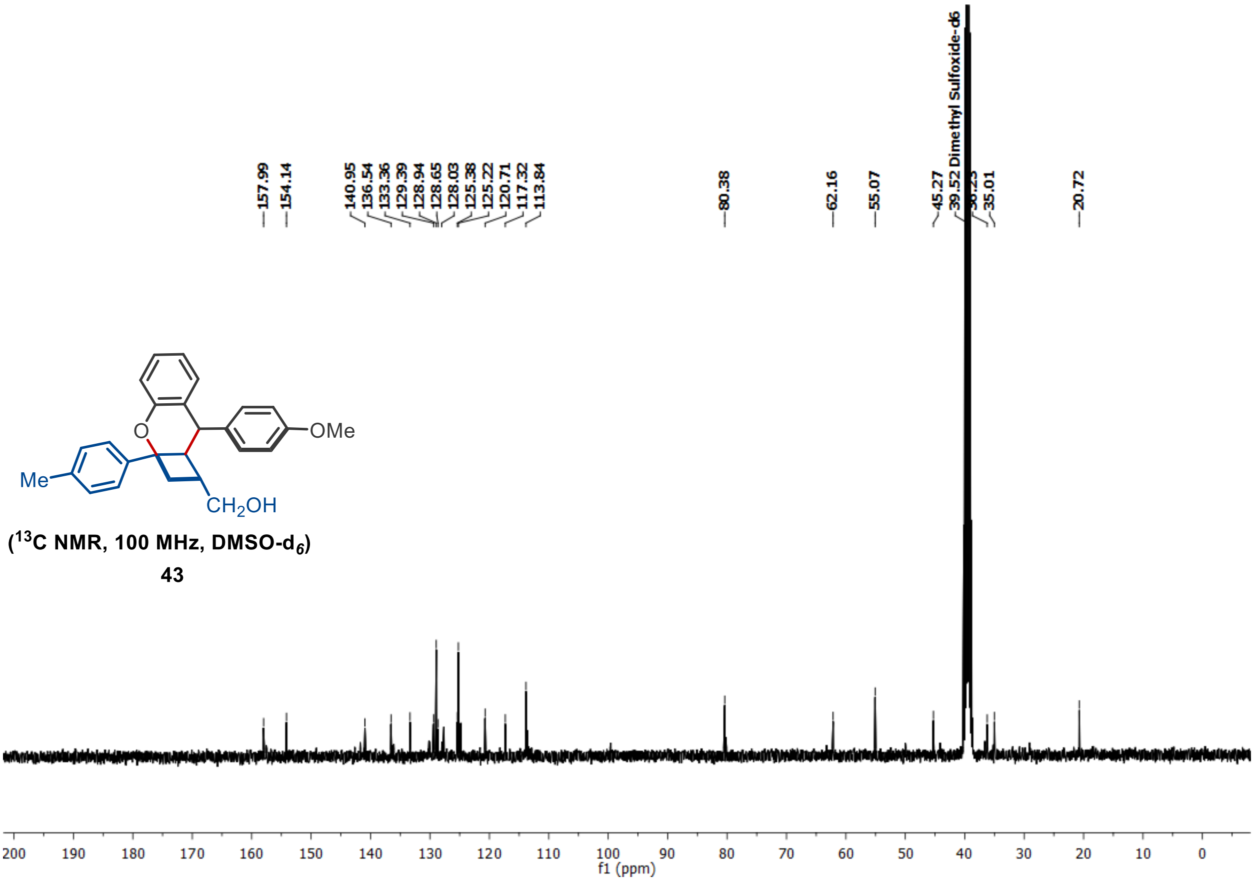

(^{13}C NMR, 100 MHz, DMSO-d$_6$)

43

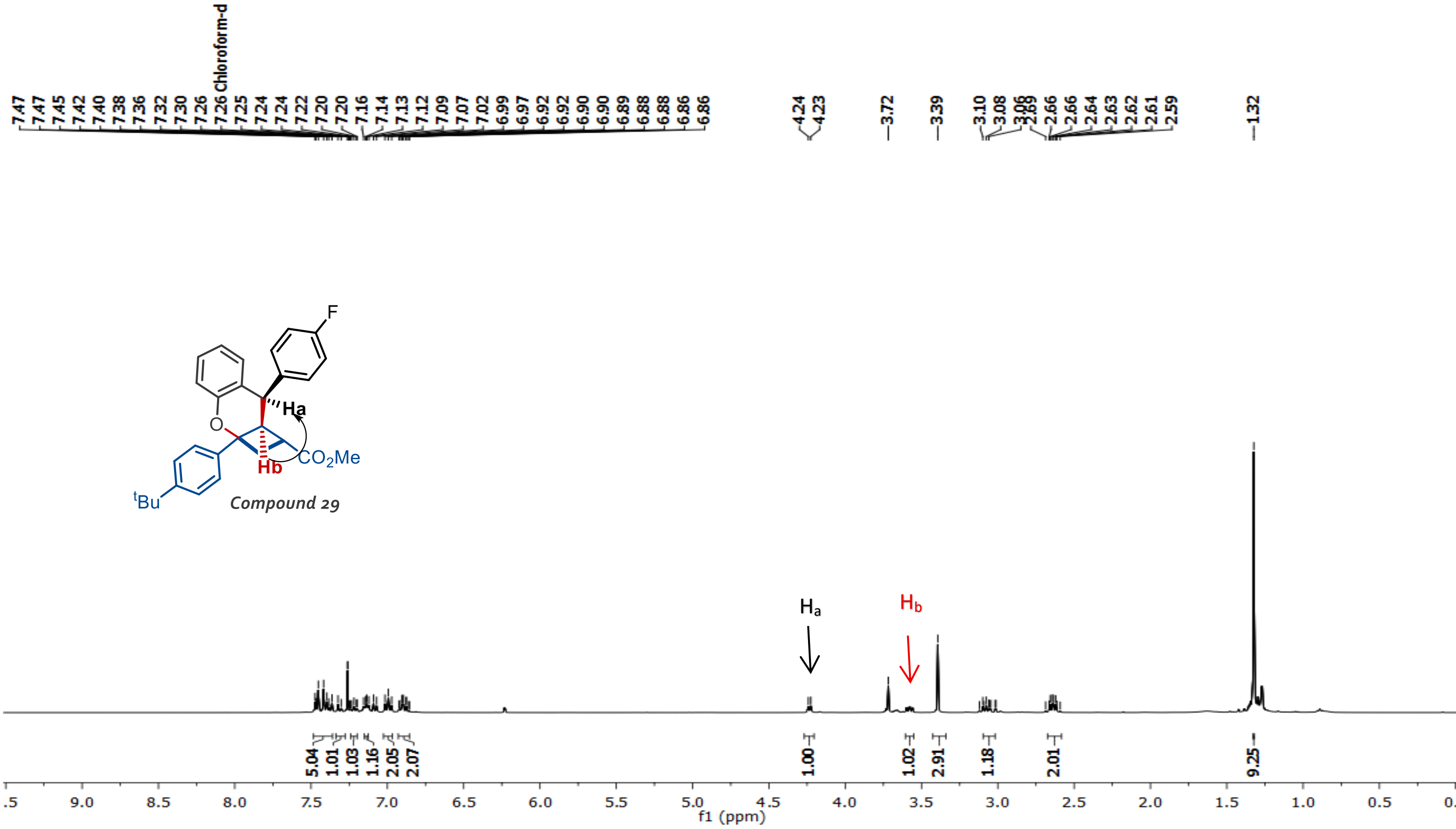

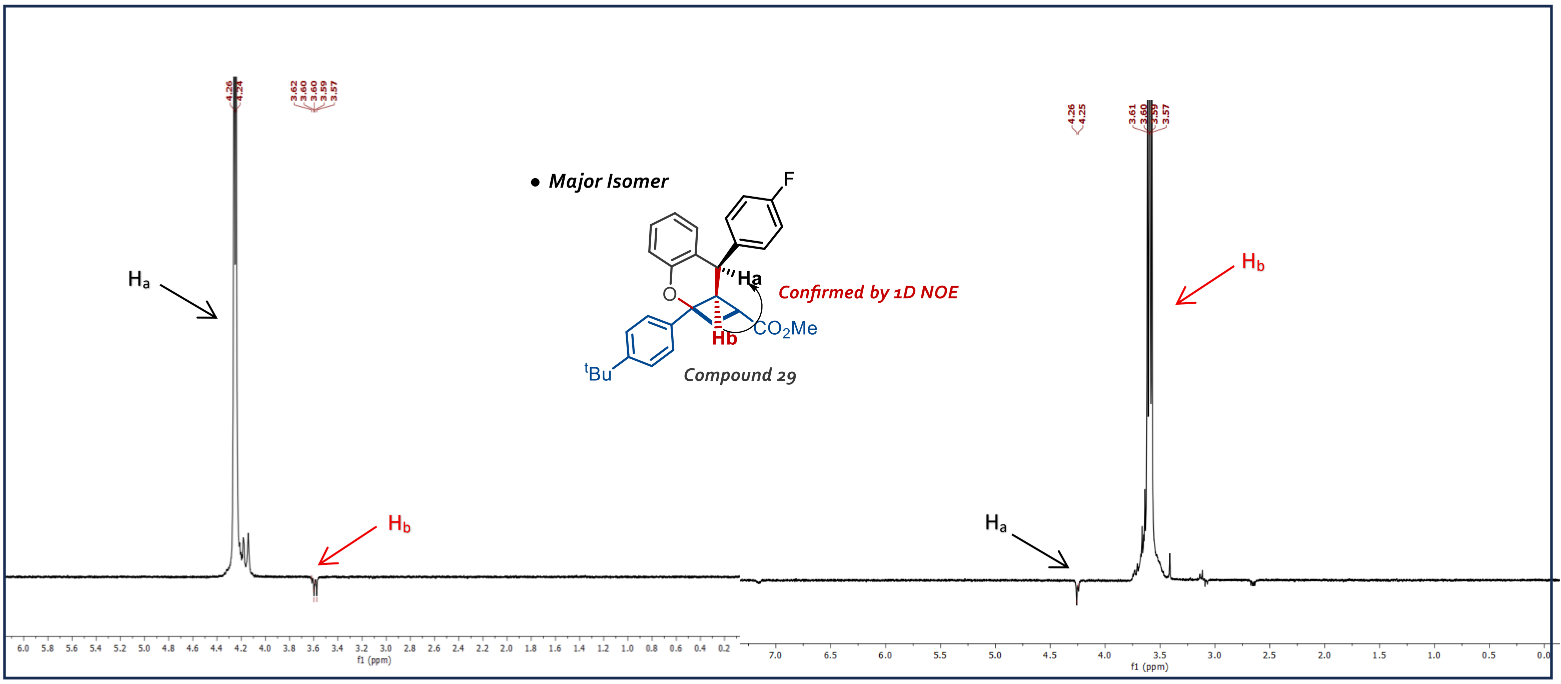

1D NOE Experiment

11. Crystallographic Data

Single crystals of compound **12** suitable for X-ray diffraction were obtained. A suitable crystal was selected and mounted on an **XtaLAB Synergy, Dualflex, HyPix3000 diffractometer**. The crystal was kept at **293 K** during data collection. The structure was solved using **SHELXT (Sheldrick, 2015)** through **Intrinsic Phasing** and refined by **full-matrix least-squares on F^2** using **SHELXL (Sheldrick, 2015)** within the **Olex2** interface. All non-hydrogen atoms in the compounds were refined anisotropically and hydrogen atoms were placed at calculated positions using riding models.

Crystallization information: Solvent system: Ethylacetate-Hexane mixture Method: Slow diffusion technique.

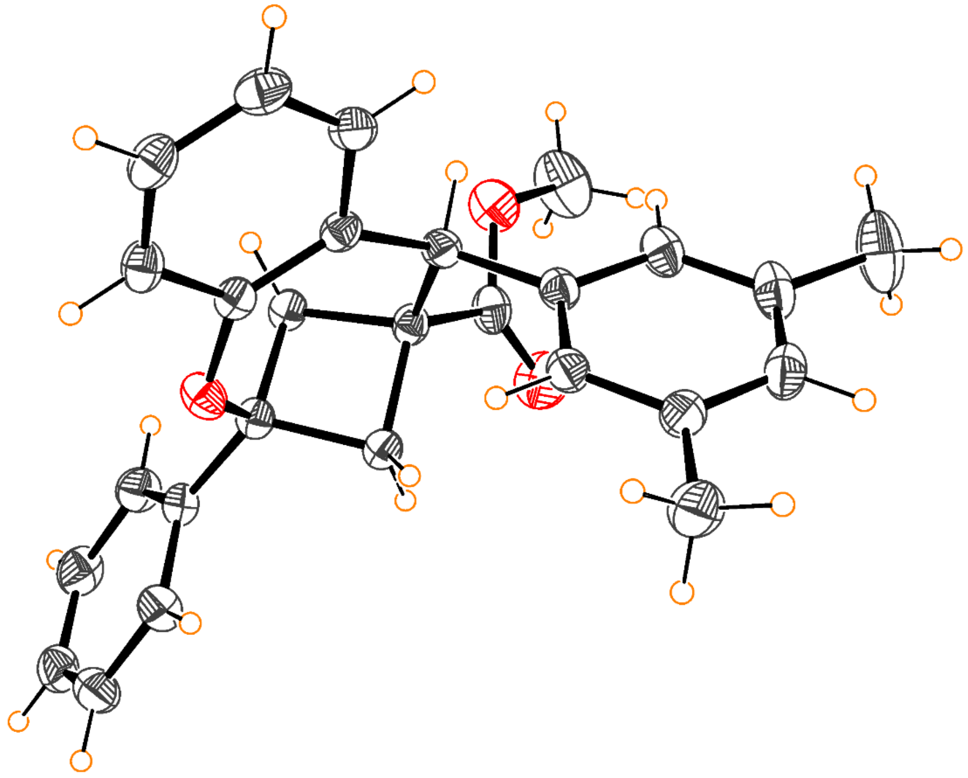

Figure S4. ORTEP diagram of compound 12 (CCDC No 2489656): Atoms are shown with 50% probability of thermal ellipsoids

Table S2. Crystal data and refinement parameters

Identification code	Compound 12
Empirical formula	$C_{27}H_{26}O_3$
Formula weight	398.48
Temperature/K	293
Crystal system	monoclinic
Space group	$P2_1/n$
a/Å	13.7834(2)
b/Å	10.8302(1)
c/Å	14.5879(2)
α/°	90
β/°	98.798(1)
γ/°	90
Volume/Å^3	2152.02(5)
Z	4
ρ_{calc}g/cm^3	1.230
μ/mm^{-1}	0.624
F(000)	848.0
Reflections collected	3916
Independent reflections	3900 (R_{int} = 0.0420, 3407 with $I \geq 2\sigma(I)$)
Data/restraints/parameters	3900/0/ 274
Goodness-of-fit on F^2	1.060
Final R indexes [I>=2σ (I)]	R1 = 0.0420, wR2 = 0.1180
Final R indexes [all data]	R1 = 0.0420, wR2 = 0.1180

12. References:

1.(a) J. Zhou, W. J. Huang, G. F. Jiang. *Org. Lett*. **2018**, 20, 1158-1161. (b) C.-Y. Wang, J.-B. Han, L. Wang, X.-Y. Tang, *J. Org. Chem.* **2019,** 84, 14258–14269.

2. (a) Dhake, K.; Woelk, K. J.; Becica, J.; Un, A.; Jenny, S. E.; Leitch, D. C. *Angew. Chem., Int. Ed*. **2022**, 61, e202204719. (b) Bychek, R. M.; Hutskalova, V.; Bas, Y. P.; Zaporozhets, O. A.; Zozulya, S.; Levterov, V. V.; Mykhailiuk, P. K. *J. Org. Chem*. **2019**, 84, 15106. (c) Guo, R.; Chang, Y.-C.; Herter, L.; Salome, C.; Braley, S. E.; Fessard, T. C.; Brown, M. K. *J. Am. Chem. Soc.* **2022**, 144, 7988.
